# New Archaeological Evidence for an Early Human Presence at Monte Verde, Chile

**DOI:** 10.1371/journal.pone.0141923

**Published:** 2015-11-18

**Authors:** Tom D. Dillehay, Carlos Ocampo, José Saavedra, Andre Oliveira Sawakuchi, Rodrigo M. Vega, Mario Pino, Michael B. Collins, Linda Scott Cummings, Iván Arregui, Ximena S. Villagran, Gelvam A. Hartmann, Mauricio Mella, Andrea González, George Dix

**Affiliations:** 1 Department of Anthropology, Vanderbilt University, Nashville, Tennessee, United States of America; 2 Universidad Austral de Chile, Valdivia, Chile; 3 Universidad Catolica de Temuco, Chile; 4 Fundación Wulaia y Sociedad Chilena de Arqueología, Santiago, Chile; 5 Departamento de Geologia Sedimentar e Ambiental, Instituto de Geociências, Universidade de São Paulo, São Paulo, Brasil; 6 Instituto de Ciencias de la Tierra, Universidad Austral de Chile, Valdivia, Chile; 7 Texas State University, San Marcos, Texas, United States of America; 8 PaleoResearch Institute, Inc., Golden, Colorado, United States of America; 9 Museu de Arqueologia e Etnologia, Universidade de São Paulo, São Paulo, Brasil; 10 Observatório Nacional, Rio de Janeiro, RJ, Brazil; 11 Departamento de Geofísica, Instituto de Astronomia, Geofísica e Ciências Atmosféricas, Universidade de São Paulo, São Paulo, Brasil; 12 Oficina Técnica, Servicio Nacional de Geologia y Mineria, Puerto Montt, Chile; 13 Facultad de Estudios del Patrimonio Cultural y Educación, Universidad SEKA, Santiago, Chile; 14 Department of Earth Sciences, Carleton University, Ottawa, Ontario, Canada; New York State Museum, UNITED STATES

## Abstract

Questions surrounding the chronology, place, and character of the initial human colonization of the Americas are a long-standing focus of debate. Interdisciplinary debate continues over the timing of entry, the rapidity and direction of dispersion, the variety of human responses to diverse habitats, the criteria for evaluating the validity of early sites, and the differences and similarities between colonization in North and South America. Despite recent advances in our understanding of these issues, archaeology still faces challenges in defining interdisciplinary research problems, assessing the reliability of the data, and applying new interpretative models. As the debates and challenges continue, new studies take place and previous research reexamined. Here we discuss recent exploratory excavation at and interdisciplinary data from the Monte Verde area in Chile to further our understanding of the first peopling of the Americas. New evidence of stone artifacts, faunal remains, and burned areas suggests discrete horizons of ephemeral human activity in a sandur plain setting radiocarbon and luminescence dated between at least ~18,500 and 14,500 cal BP. Based on multiple lines of evidence, including sedimentary proxies and artifact analysis, we present the probable anthropogenic origins and wider implications of this evidence. In a non-glacial cold climate environment of the south-central Andes, which is challenging for human occupation and for the preservation of hunter-gatherer sites, these horizons provide insight into an earlier context of late Pleistocene human behavior in northern Patagonia.

## Introduction

The initial peopling of the Americas is a long-standing topic of much interdisciplinary debate. Most of this debate has centered on the timing and place of initial human arrival, the number of migrations, the character and integrity of the archaeological evidence, and the extent to which North American technologies and economies spread southward, affecting the first peopling of South America [1–7]. In recent years, there is an emerging consensus that people arrived in North America at or before 15,000 years ago, as suggested by several site discoveries over the past few decades [8–16]. There also is general agreement that people migrated from Asia to North America across Beringia, and then dispersed to Central and South America and that the old Clovis-first model of human entry around 13,000 years ago no longer explains the peopling of the New World. With the recent demise of this model, new questions are being addressed and new models proposed: such as whether there were multiple migrations by different peoples from different places [4–5,17–20], whether they arrived by land, along coastlines or both, and whether the first inhabitants of the Americas were the same people whose descendants inhabited the hemisphere upon the first arrival of Europeans in the 1500s.

Human genetic and skeletal studies provide different types and scales of information and varying opinions on the origin and diffusion of early South Americans [17–19]. Archaeologists generally disagree about the origin of South American material culture. Two different perspectives have been proposed to explain the earliest known stone tool technologies, each with varying implications for the interpretation of early sites. The first is that Clovis bifacial technologies reached South America ~13,000 cal BP in the form of Fishtail projectile points [3,6]. This model is based on diffusion and comparative morphological analyses of fluted point styles and leaves little room for independent technological development in South America. The second is that North and South American tool assemblages, including both bifacial and unifacial industries, are different adaptations to different environmental and cultural conditions, yet both derived from an earlier currently undefined technology somewhere in East Asia [1,4,5]. The arguments advanced in support of the second model at present hinge on evidence recovered from sites such as Monte Verde II in south-central Chile [13,14], Gault and Friedkin sites in Texas [9,10], Cactus Hill in Virginia [11], Paisley Cave in Oregon [8], and possibly other sites [12,15,16,18,20–22], all of which contain varying types of bifacial and unifacial assemblages dating ~14,000 cal BP or earlier. There is no doubt that some lingering influences and contacts existed between North and South America, but some of these appear to have taken place after the initial colonization of the southern hemisphere and, in some later cases, may even represent reverse migrations from south to north [7].

**Fig 1:**
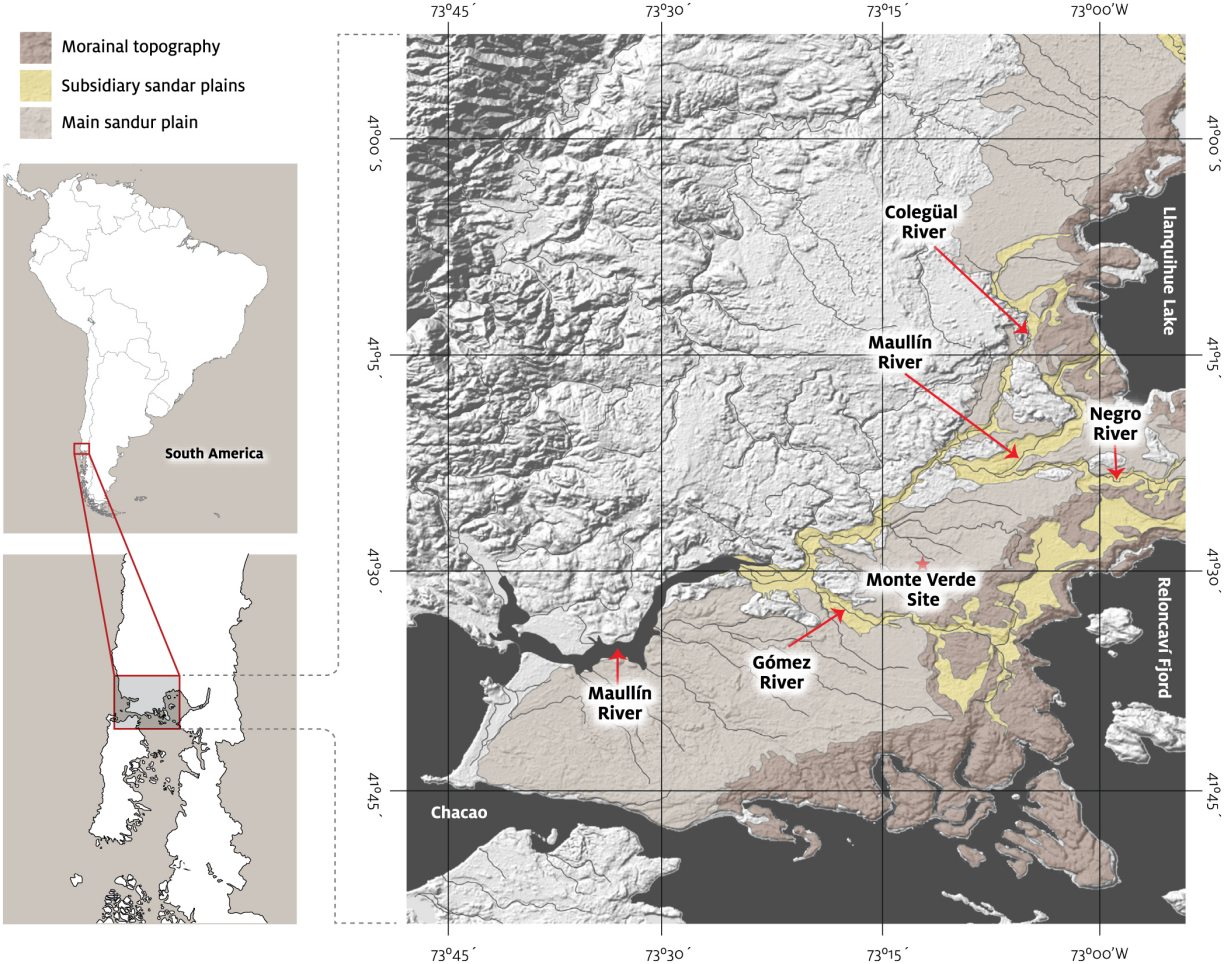
Geomorphological context of the Monte Verde site within the wider context of the Llanquihue drift. During the last glacial period, piedmont glaciers descended from Andean ice fields to the east occupying and sculpting what today are the Llanquihue Lake and Reloncavi fjord basin. The moraines are complex features representing more than one glacial advance in this terminal position. The primary sandur plains drained glacier discharge during successive advances until ~22,000 cal BP. The latest glacier advance ~17,600 cal BP culminated in the inner ridges, near the present-day lake and sea shorelines of the Llanquihue Lake and Reloncavi basin, respectively, draining through the subsidiary sandur plains of the Río Maullin, Río Negro and Río Gomez [31].

For almost four decades the late Pleistocene site of Monte Verde II (MV-II; Fig 1) has played an important role in interdisciplinary research on the dating and nature of the initial peopling of South America [13,14, 23,24,25]. Recent excavations at the site and the nearby locality of Chinchihuapi have revealed new cultural evidence that strengthens the possibility of an earlier human presence on the continent. The new evidence is multiple, spatially discontinuous, low-density occurrences of stratigraphic *in situ* stone artifacts, faunal remains, and burned areas that suggests discrete horizons of ephemeral human activity radiocarbon dated between ~14,500 and possibly as early as 19,000 cal BP. The character of some of these horizons may not meet the traditional criteria used by some archaeologists to define valid early sites, such as spatially continuous and multiple activity areas with numerous features, artifact clusters, and diagnostic bifacial stone tool assemblages [6,26,27]. In recent years, however, these expectations have been challenged by the discovery of new evidence suggestive of a wider diversity of tool types and of more ephemeral human behavior, landscape use, and site size and structure [8,12,15,16,22,28–30]. These data suggest that people might have been in South America before 15,000 years ago, were highly mobile, and seasonally adapted to a wide variety of environments, including cold non-glacial environments.

**Fig 2:**
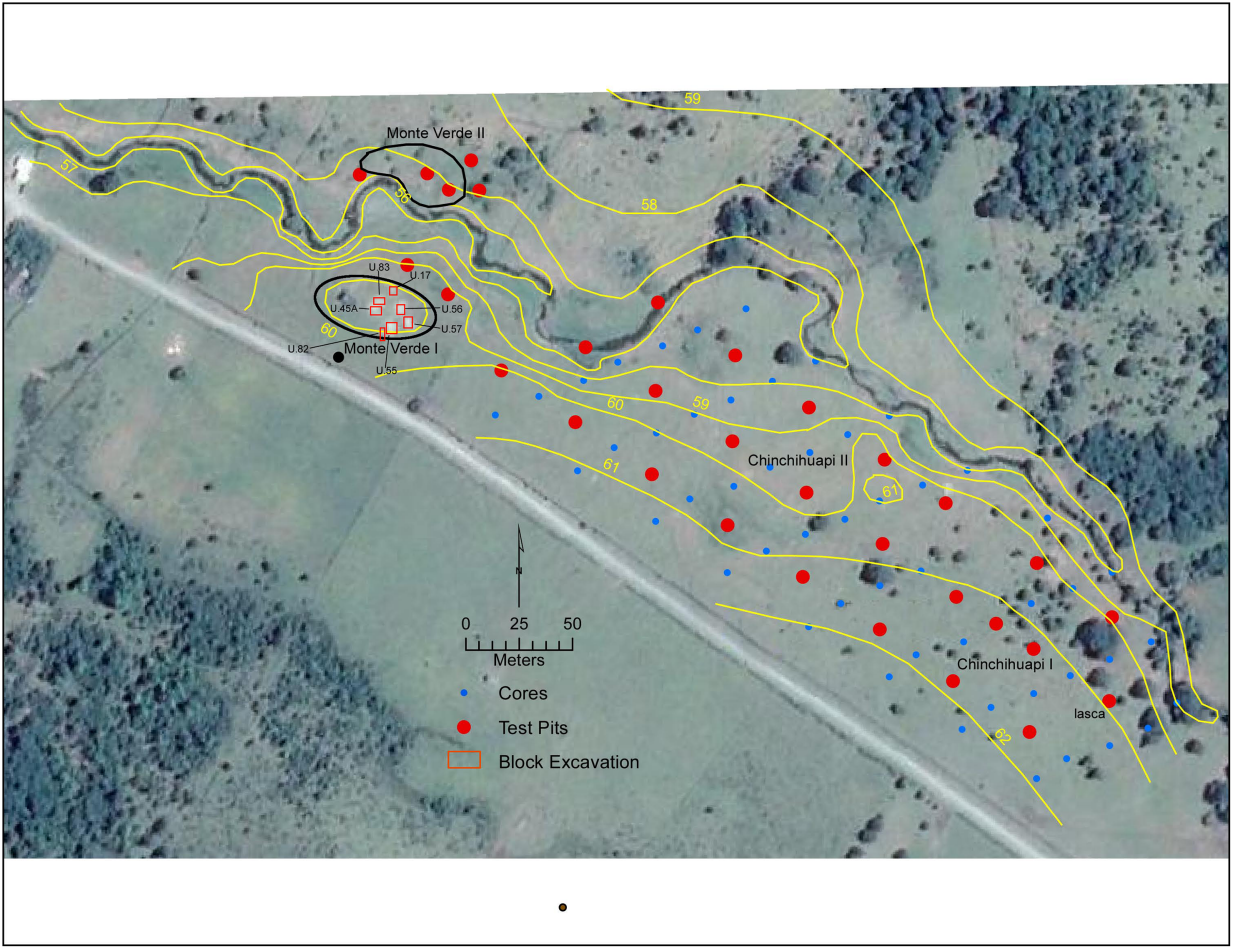
Map of the Monte Verde and Chinchihuapi sites showing the different site sectors, block excavation, test pits and cores carried out during the 2013 excavation season.

Previous work at Monte Verde revealed one valid human site (MV-II) dated ~14,500 cal BP and adapted to a cool, temperate rainforest and an older possible cultural horizon (MV-I) associated with a cold, non-glacial environment (Fig 2) [13,14]. MV-II is a campsite buried in the north terrace of Chinchihuapi Creek, which formed around 15,000 cal BP, and associated with the remains of a long tent-like dwelling, the foundation of another structure, hearths, human footprints, economic plants, and wood, reed, bone, and stone artifacts. Although bifacial projectile points, flaked debitage, and grinding stones were recovered, most lithic tools were edge-trimmed pebble flakes and sling and grooved *bola* stones. Also investigated previously was the Chinchihuapi site, represented by two distinct localities (CH-I and CH-II) located on the south side of the present-day creek ~500 m upstream from MV-II. Although only preliminarily investigated, it also dated ~14,500 cal BP and yielded a few burned areas and fragments of scorched animal bone directly associated with a few pebble flakes similar to those recovered at MV-II. MV-I dated ~33,000 BP and initially defined by scattered occurrences of three clay-lined, possible culturally-produced burned areas and twenty-six stones, at least six of which suggest modification by humans. This prior archaeological evidence from MV-I was too meager and too laterally discontinuous to falsify or verify its archaeological validity [14,30]. MV-I is buried in a sandur plain, the Salto Chico Formation (SCH-Fm), which formed as part of the Llanquihue drift during the last glaciation [31] by meltwater from glaciers located 7.5 km to the southeast (Fig 1). Previous research did not record any cultural material in strata spanning the multi-millennia time period between the MV-I and MV-II sites [14].

**Fig 3:**
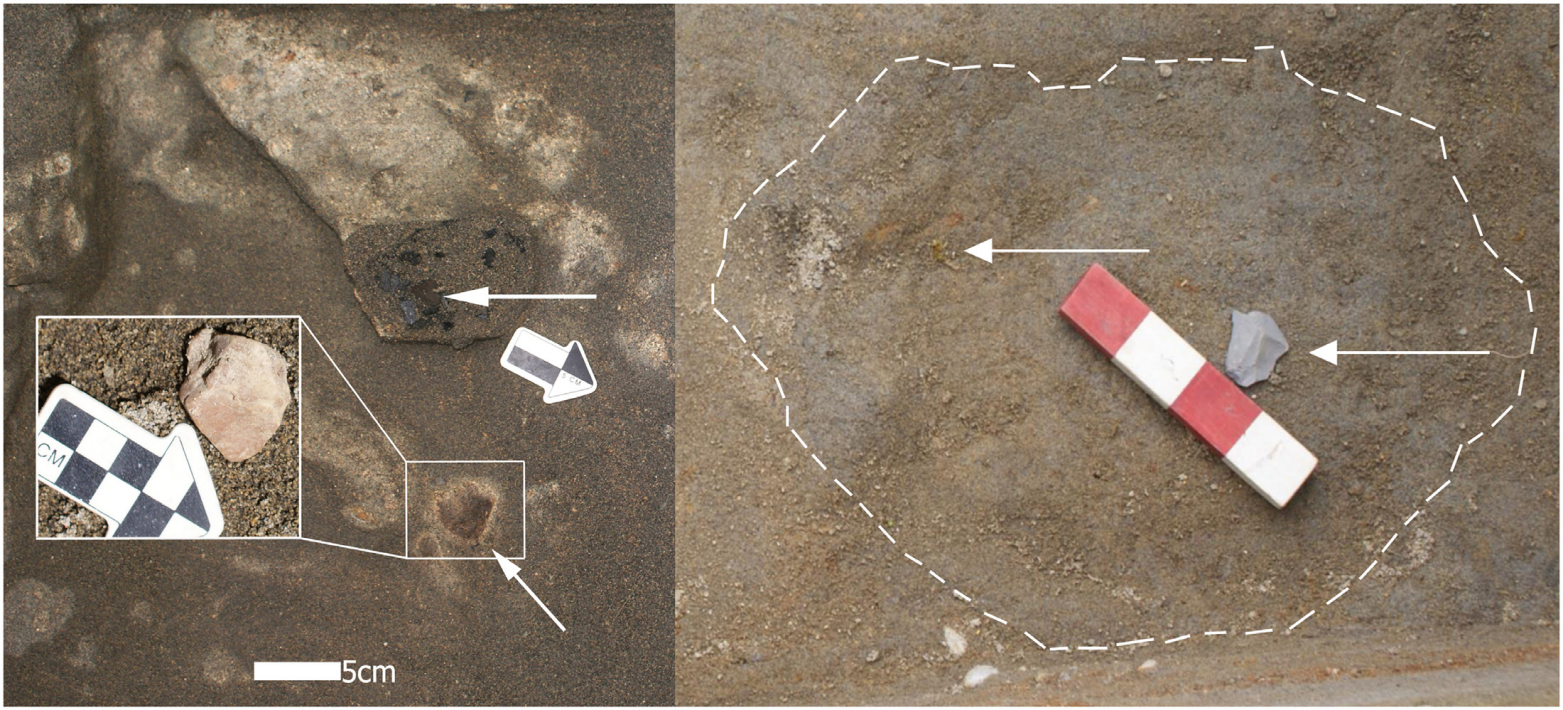
Excavated archaeological features. a: *in situ* burned feature (No. 3) with charcoal, burned bone fragment (top arrow), and ash associated with an embedded, knapped flake of unsourced limestone (bottom arrow) in Unit 56, site MV-I. The thin, light yellowish orange (10YR 8/3) tephra lens has been removed in and around portions of the feature to expose the underlying gray sand. Inset shows a close-up of the flake after the overlying lens was brushed from its upper surface; b: *in situ* scorched bone fragment (left arrow) and a percussion flake of unsourced limestone (see S7f Fig) directly associated with a burned feature (No. 6, dotted line defines dark ashy areas) in Unit 56, site MV-I.

The principal goal in undertaking new research at Monte Verde was to conduct a preliminary, discontinuous geo-archaeological reconnaissance of the Monte Verde area to determine whether we had previously defined the wider horizontal and deeper vertical extent of the site for the National Council of Monuments in Chile (CMN). It was not intended to be a full-scale excavation to resolve previous research questions [14]. The subsurface testing and excavation plan thus was largely designed by the CMN. Within this plan, we developed three specific objectives: 1) to explore previously unknown geo-archaeological deposits in sites MV-I, MV-II, CH-I and CH-II; 2) to investigate the long time span between sites MV-I and MV-II; and 3) to further assess the geological setting of the sites by applying sedimentological, microstratigraphic, magnetic, optically stimulated luminescence dating (OSL), and macro- and micro-botanical analyses. Based on our previous findings at MV-I, which revealed possible cultural evidence laterally dispersed in deeper, sandy levels of the sandur plain, our recent work centered on spatially intermittent excavations and core drillings across a 500 m area between the MV-I and Chinchihuapi sites in search of additional scattered remains down to and below these levels (Fig 2). The result was the discovery of twelve small, discrete burned features directly associated with fragments of burned and unburned faunal remains, spherical and manuport stones, and human-knapped flakes dated by ^14^C and OSL means between at least `18,500 and 14,500 cal BP (Fig 3, S4 and S5 Figs). The features and associated lithics and bones are spatially limited within discrete lenses, averaging ~33 by 42 cm in spatial extent and ~1.0 to 2.8 cm in thickness. Only one excavated unit, measuring 5 by 5 m in size, contained more than one feature, indicating their widespread and intermittent dispersion across the study area. Although both horizontally and vertically discontinuous, these remains appear to represent ephemeral seasonal activities laterally spread across uneroded, slightly elevated surfaces (~0.5–0.8 m high) between small, narrow and shallow channels of a braided drainage system buried in the SCH-Fm (Fig 2 and S1 Fig).

## Materials and Methods

During two field seasons in 2013, fifty-four archaeological cores between ~1.5 to 2 m in depth were placed systematically across the study area to define the presence or absence of archaeological remains on the south side of the present-day Chinchuapi Creek (Fig 2). Twenty-five systematically placed test pits measuring 1 by 2 m to depths from 1.2 to 2.1 m also were excavated. Considering the previously defined dispersed nature of possible archaeological deposits in the MV-I site [13,14], the distance between test units, which were distributed along three parallel transects, was 25 m from each other. The cores and test pits covered a total area of approximately 20,000 m^2^ between the MV-I and CH-I sites. In addition, ten excavation blocks (Units 17, 45(A), 55, 56, 57, 81 and 82 in MV-I, Units 5 and 6 in CH-I, and Unit 83 in CH-II, see Fig 2) measuring between 2 by 4 m and 5 by 6 m were excavated to depths of 2.1 to 2.7 m. Five test pits of 1 by 2 m or 2 by 2 m were placed in the MV-II site to a depth of ~1.5–1.7 m. Geological coring and trenching was opportunistic across the sites to open additional sediments for OSL dating and sediment sampling for micromorphologic, magnetic and other stratigraphic analyses. In total, 39 new archaeological lithics and twelve burned features, concentrated primarily within a 30 by 35 m section of the MV-I site and secondarily within a 10 by 12 m section of the CH-I site, were recovered. As discussed below, this widely scattered evidence suggests an intermittent and ephemeral human presence over a few millennia and indicates the laboriousness of searching for early archaeological remains in this type of setting.

The methodologies of specific interdisciplinary studies are presented in Texts A-H in S1 File. Analyses of data reported in several of these studies are ongoing, thus some results are preliminary. All necessary permits for the field and laboratory research reported here were obtained from the National Council of Monuments (No. Proyecto FIC–526), Santiago, Chile. The research complied with all relevant regulations. The excavated archaeological materials studied here are Features 2013/1-12 and Lithics 2013/1-39 permanently stored at the Universidad Austral de Chile in Valdivia and are available for further analysis.

### Geomorphology and Stratigraphy

A key component of the recent work at Monte Verde involved large-scale correlation of the local geology with established regional paleoenvironmental records from nearby Andean glaciers and volcanoes (Fig 1) and from pollen, starch grain, and phytolith studies (see Texts C-G in S1 File). This approach had the benefits of improving stratigraphic age control, helping to develop predictive models to identify future sites in the region, and informing the development of new questions about how people used Monte Verde and similar landscapes.

During the late Llanquihue glaciation, successive glacial advances culminated at the outermost moraine complex at ~26,000 cal BP and later at ~22,000 cal BP [31] (Fig 1). These moraines were directly linked to the main outwash plain where Monte Verde was emplaced, that is, the fluvial system in the study area was part of a much wider surface directly draining glacier discharge. Drainages under this regime were heavily sediment-loaded and developed a braided network, with frequent but shallow channel migrations and network reconfigurations, thus effectively spread across a broad surface. These conditions deposited the SCH-Fm during successive advances prior to ~40,000 cal BP and between ~33,000 cal BP and 22,000 cal BP. Younger advances after this period did not reach the outermost moraines, but instead stopped at the inner ridges, roughly coincident with the current Llanquihue Lake and the Reloncaví bay shorelines (Fig 1). Drainage of the glacier lobes, however, did not engage directly with the main outwash plain where the Monte Verde and Chinchihuapi sites are located but instead was channeled through subsidiary sandur plains of the Río Negro and Río Gómez drainages for the Reloncaví lobe and the Río Maullín drainage for the Llanquihue lobe. The Monte Verde site area thus was never in direct contact with the glaciers and moraines; nevertheless, cold conditions prevailed, inhibiting soil development. Small, seasonal streams running through the plain were fed by snowmelt during warm seasons, which account for abundant sediments of the SCH-Fm.

These conditions ended with glacier retreat ~19,000 to 17,600 cal BP. It is from this time onwards that soil development began to stabilize the plain and the streams were restricted in sediment availability. The new archaeological evidence suggests that this also was the time when people began to explore the Monte Verde area. The drainage regime then changed from a snow-melt influenced condition to a low-energy pluvial setting. This restriction eventually promoted the incision and entrenchment of Chinchihuapi Creek around 15,000 cal BP. Most of the archaeological horizons reported here date between ~19,000 and 14,500 cal BP in the MV-I, CH-I and CH-II sites, and are located next to small meandering or braided drainages flowing across the sandur plain prior to incision of creek. (Only the MV-II site and the upper cultural levels of the CH-I site are associated with the Chinchihuapi Creek setting.) These drainages would have had seasonally variable discharges, with maxima during the estival season. Regional and local pollen records suggest that the plain was covered by patchy subantarctic parkland between ~20,000 and 17,600 cal BP (S1 Fig). After ~17,600 cal BP, the environment changed to an open North Patagonian forest and after 15,000 cal BP to a dense, cool Temperate Rainforest [13,14,32,33]. Like today, it is possible to infer strong seasonal variation with prolonged cold periods in the winter, flooding in the spring, and extensive grass cover and reduced water flow in the summer and early autumn.

**Fig 4:**
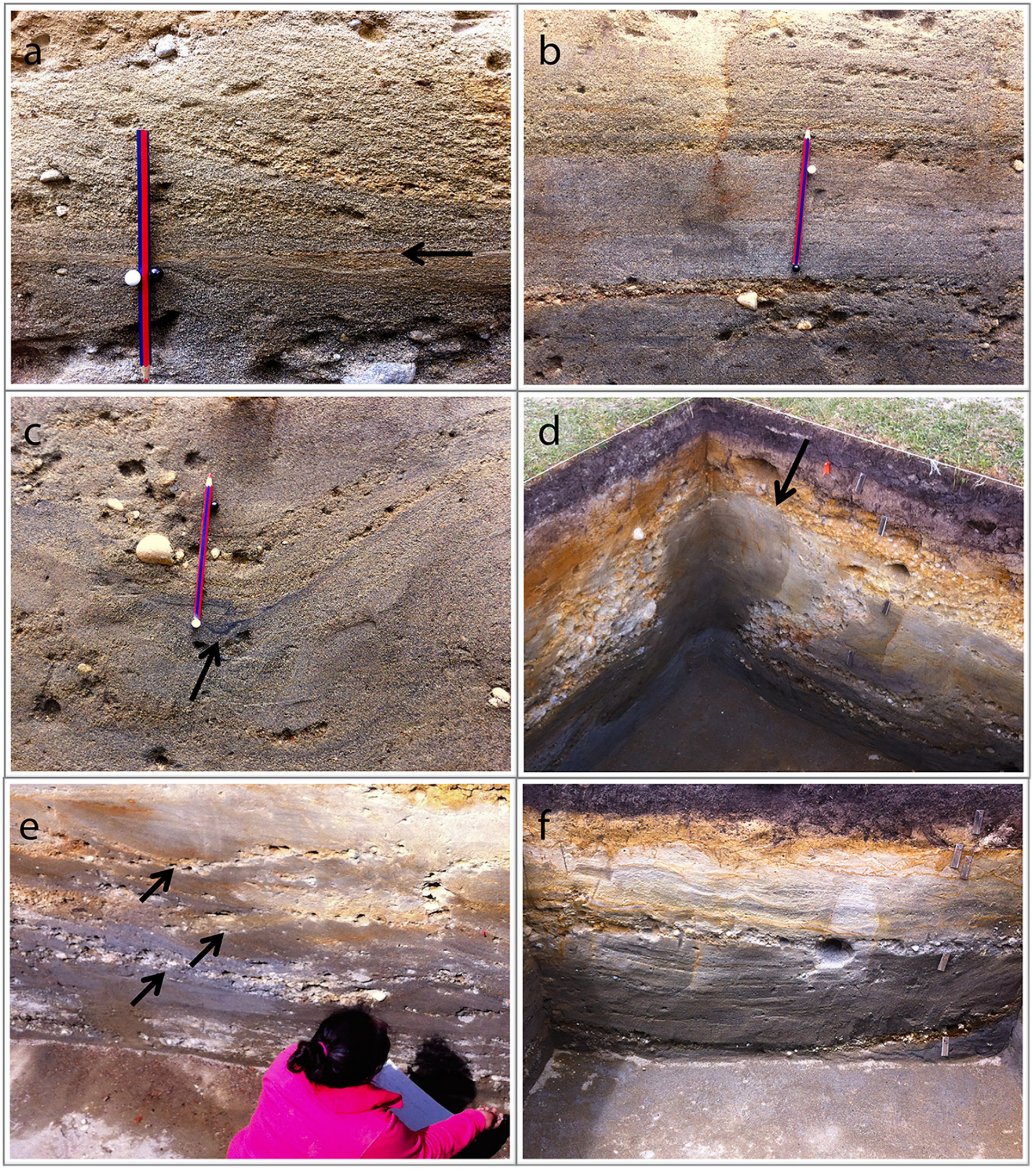
Sedimentary attributes of SCH-4. a: Planar cross-stratified sand, note the rippled reactivation surface (arrow, Unit 45-A); b: Parallel/planar cross-stratified sand capped by thin, intermittent small-gravel layers (Unit 55); c: Soft sediment deformation structures; note the small fold in the organic layer beneath gravelly sand (arrow, Unit 17); d: Gravel layer disrupted and submerged under a liquefied sand outburst (Unit 56); e-f: Trough cross-bedded sand with thin gravel lag deposits in the bottom (Units 82 and 57, respectively).

The SCH-Fm outcrops within the site of MV-I and is composed of four members [34,35], all characterized by mixtures of coarse to medium sand and gravel (Fig 4). The youngest three members (SCH-1 to 3) outcrop outside of the Monte Verde area and later were eroded when the Chinchihuapi Creek developed. The fourth, SCH-4, comprises medium and fine sands, including thin gravel and tephra lenses. Trough cross-bedded strata are less common and are related to bar dissecting and small second- or third-order drainage channels. Where present, they conform 1 to 1.5 m thick layers with ~0.5–1.5 m wide and ~0.15–0.85 m deep troughs. Small pebble-sized (0.5–1.1 cm) lag deposits typically characterize the base of each trough. Soft-sediment deformation structures are widespread. Unstructured, though well-sorted sand bodies are ubiquitous. These SCH-4 deposits are interpreted as thin, discrete, fluvial channel facies. Planar cross-bedded and parallel-stratified sand bodies capped by thin pebble layers represent armored sand bars, that is, lateral or downstream accretion elements. These facies are not associated with a stable stream but with a secondary braided network subject to intermittent activity in response to seasonally variable discharge. These abundant unstructured sand facies were vegetated bars subject to limited, low-energy flooding during ice-melt seasons.

**Fig 5:**
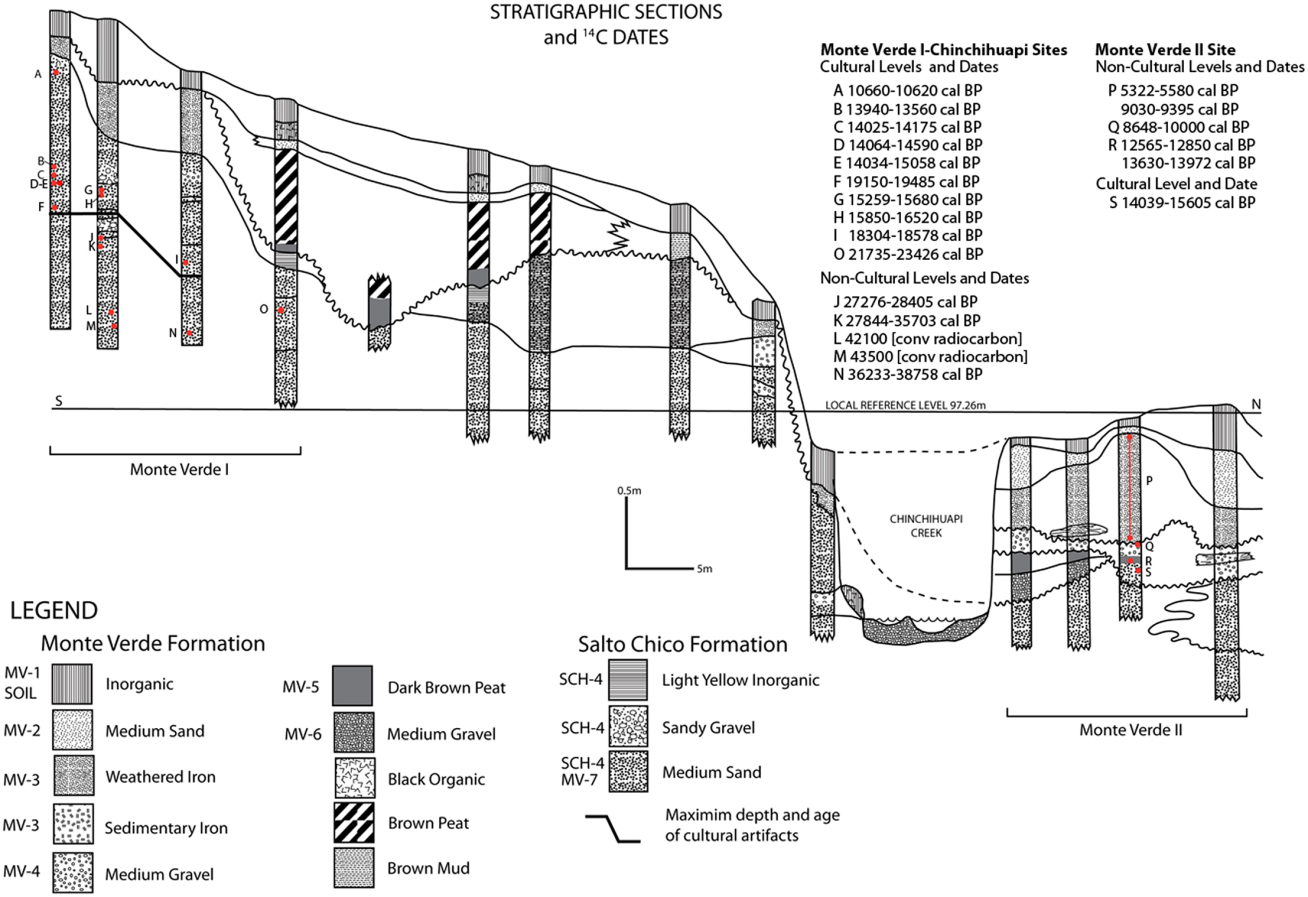
Schematic representation of the stratigraphic cross-sections of the MV-I and MV-II sites divided by the drainage of Chinchihuapi Creek. Location of the corresponding radiocarbon dates and cultural and non-cultural levels in the MV-I and MV-II sites also are shown. Not shown is the stratigraphic profile for the CH-I and CH-II sites; however, their profiles are comparable to that for the MV-I site. The dates listed under the MV-II site represent the range of radiocarbon assays for the overlying non-cultural strata MV-3 (P), MV-4 (Q) and MV-5 (R) and for the single cultural component of MV-II on the buried surface of stratum MV-7 (S). Since these dates were published previously [13,14,25], their laboratory numbers are not provided here (see Table 1). Radiocarbon dates in site MV-I are in stratigraphic order but at slightly different depths due to the downward sloping strata in site MV-I (see Text B in S1 File).

In the MV-I site, SCH-4 is represented by strata MV-7, a gray sand (2.5YR 5/1), and MV-8, a bluish gray sand (10BG 4/1), which were deposited by seasonal spring and summer meltwater of the frontal ice lobes to the southeast. These strata show a characteristic fabric indicative of several narrow, shallow, low-energy drainages within a braided system. Based on local litho-stratigraphic correlations and ^14^C assays, a time span between ~35,000 and 19,000 cal BP was determined for SCH-4 [34,35] (Fig 5). The lower MV-8 stratum shows little internal stratification and no pedogenic development. In contrast, the bedded and pedogenically modified lower levels of stratum MV-7 represent slight reworking of the deposits by the narrow, ephemeral braided channels running across portions of the plain of the original outwash flow. In general, MV-7 represents the transition to a wetter depositional environment, as evidenced by sediment deformation and the presence of small braided (~1.5 m maximum width and 0.8 m maximum depth; see Fig 4c) channels (see Texts C-G in S1 File). The middle and upper levels of MV-7 represent a continuation of this environmental setting, with deposition also by small, shallow channels flowing with intermittent, diffused, low-energy flow regimes.

The newly recovered archaeological horizons rest on or are embedded in thin, highly oxidized, stable tephra lenses (~0.3–3.1 cm thick) deposited by nearby volcanoes (see Text C in S1 File) at different depths and locations on uneroded surfaces of the buried braided system. These horizons are associated with the upper to upper lower sediments of stratum MV-7 (generally ~0.90–1.50 m below present-day ground surface) and deposited prior to the incision of Chinchihuapi Creek ~15,000 cal BP. The source of the lenses was airborne tephra that included ash and pumice lapilli. Low-energy, probably rainwater, runoff during the summer months removed much of the lighter pumice (see Texts B and C in S1 File), leaving the thin crystal deposits of pumice across elevated areas between drainage channels. Warm summer temperatures aided the precipitation of iron hydroxide colloids on the tephra, preserving the thin orange lenses upon which past humans camped and left behind the cultural features and artifacts (see S2 and S3 Figs). The presence of these lenses, twelve of which contain cultural features and artifacts, attest to their stability and integrity and to minimal impact by surface modification due to flooding at the time of or after their deposition and later by other potential disturbances (e.g., animal burrowing, tree roots, and solifluction). There is some evidence of solifluction but none directly documented with the features and cultural materials.

The research team, composed of several geomorphologists, sedimentologists, geoarchaeologists and volcanologists, was acutely aware of the various dynamic processes that could have produced important modifications in the stratigraphy, context, and structure of the archaeological materials in this early sandur environment. As a result, the team was very cautious in employing detailed geo-archaeological and geological measures to detect any taphonomic disturbances caused by natural or other processes. Yet, we discovered no mixed stratigraphic levels in the archaeological contexts, no subsequent natural retouch and water or wind patina on the edges of artifacts, and no frost shattering of bone remains. We identified limited fluidization and minor stratigraphic disturbance in two excavation units (e.g., see Fig 4d) that are attributed to earthquakes or other processes but these were not directly associated with the archaeological horizons studied here. There also was no regularity in the strike and dip of elongated bone and stone materials to suggest fluvial, rootlet or other disturbance. All bone and stone artifacts were in horizontal positions, with their longitudinal axes oriented in different directions. Furthermore, there are no artifacts that are slope-oriented because the sandur plain in the study area is relatively flat. All ash and flecks of charcoal are embedded horizontally in the features, are the result of burned plant material, and are different from and rest on or embedded in the thin pumice lapilli lenses. This is not to say that there has not been some disturbance to ash, charcoal and light-weight artifacts comprising the features, because a few small bone and lithic flakes (>3 mm) and charcoal flecks possibly have been displaced several centimeters as indicated by unidirectional ash and charcoal scatters outside three features. However, we found no hard evidence for moderate to heavy disturbance. The majority of the features are intact and in situ. It is most probable that more extensive excavation will reveal more horizons across the buried plain.

The intactness of the horizons is also suggested by the tephra, micro-stratigraphic, magnetic, other sedimentological, and luminescence studies presented in the Texts C-E and Text G in S1 File. Intactness probably was facilitated by the location of features and artifacts on the slightly higher, uneroded surfaces between the narrow and shallow water channels of the braided system and by rapid growth of grass and other vegetation over and around these features during warm months. This is a condition that quickly occurs today during warm, humid summers when similar thin, oxidized lenses are rapidly produced in shallow, standing pools of water and vegetation (~2–4 cm deep; S3c Fig). Several species of grasses were identified by pollen and phytolith analyses of the burned features (see Text F in S1 File), which suggest the use of these features during warm seasons. It also is likely that good preservation of the documented archaeological materials resulted from over-ridding deposits from low-energy debris flow [36,37], which was non-erosive on the strata on which they spread and on which the cultural materials were deposited.

Four burned or scorched and four unburned animal bone fragments, and thirty culturally produced lithics and/or exotic stone manuports were embedded in the surface or contained within the thin, mixed charcoal and ash sediments of the burned features (Fig 3 and S4 Fig). *In situ* burning of the features is indicated by a light pinkish gray discoloration (2.5YR 8/1) of the underlying 2–4 mm of sand. Other worked lithics were within a few centimeters of and on the same horizontal surface as the features and bones. The majority of the lithics have specks of charcoal powder embedded in micro-fissures suggesting direct association with the features. These associations also suggest the intactness and human intervention of the features.

## Chronology

**Fig 6:**
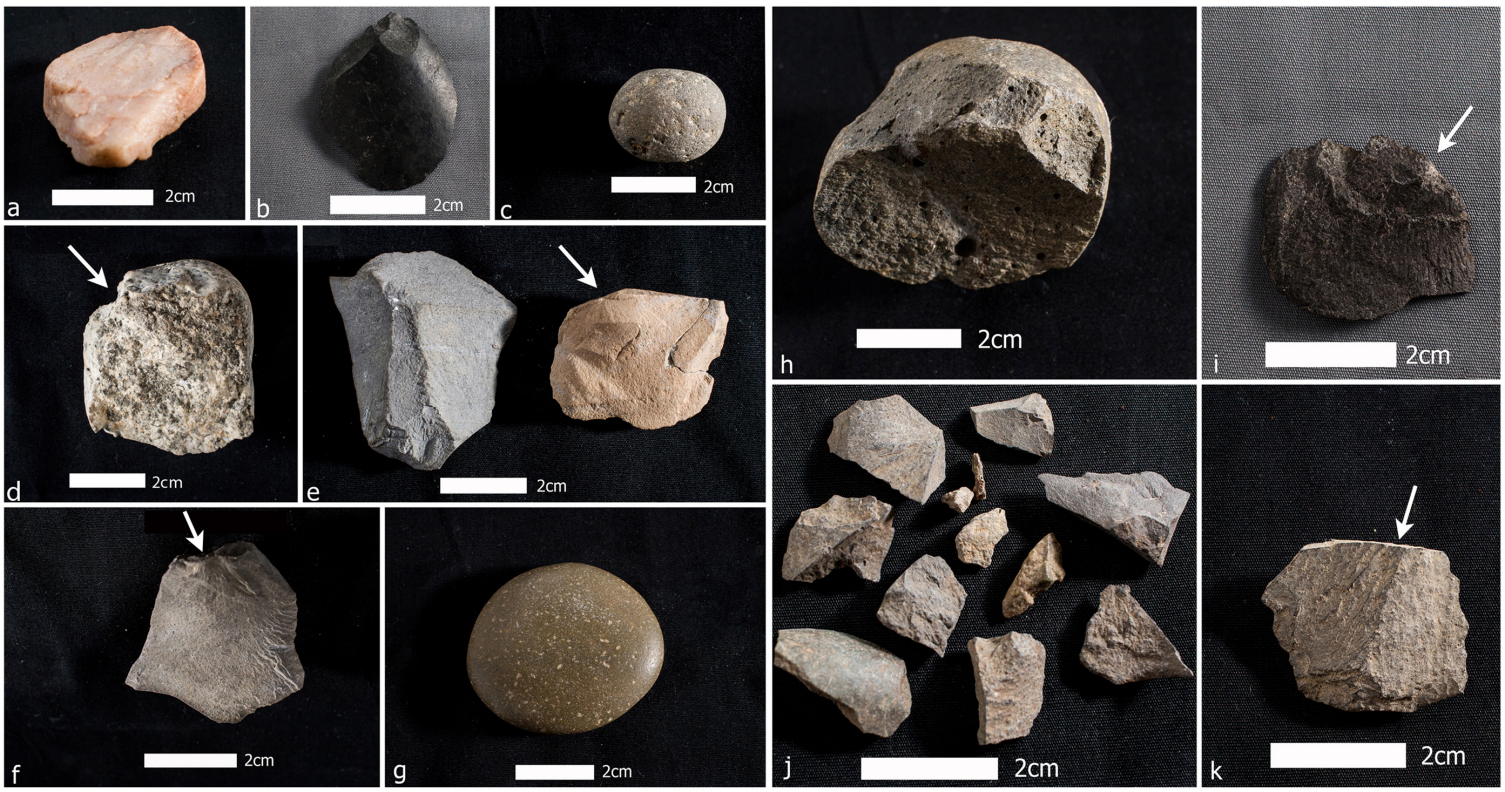
Various cultural stone tools and manuports from the 2013 ^14^C dated archaeological horizons in sites MV-I and CH-1, classified by Collin’s typology of Groups (43). a: Group 23 manuport of a fractured pinkish quartzite probably derived from a beach of the Pacific Ocean; b. Group 5f pebble flake tool of basalt showing multiple percussion facets; c. Round Smooth possible sling stone of andesite, note the dull matte finish; d. Group 5f pebble flake tool of rhyolite (worked edge at the bottom, arrow shows percussion facet); e. two Group 5f knapped flakes of exotic, unsourced limestone raw material, note the striking platforms and bulb of percussions [arrow]; f. Group 5f pebble flake tool of basalt, note the striking platform and bulb of percussion at top [arrow]; g. Exotic discoid manuport of unknown raw material probably from a beach of the Pacific Ocean; h. Group 5f knapped core of andesite with multiple percussion flake scars; i. Group 5f flake tool of basalt, note the striking platform and bulb of percussion [arrow]; j. flake scatters of basalt produced by knapping; k. Group 5f flaked tool of basalt, note the striking platform [arrow] (see S1 and S2 Tables for more details and S7 Fig for drawings of selected lithics).

The chronology of sediments and archaeological horizons was determined by ^14^C and OSL dating (Table 1; see Texts B-G in S1 File). Nine radiocarbon dates were obtained from single pieces of charcoal and from animal bone fragments in the spatially discrete burned features and reported at the 95.4% probability range (Table 1; Figs 3 and 5, and S4 Fig). A date of 10,660–10,620 cal BP on wood charcoal in the basal level of stratum MV-4 in Unit 6, site CH-1, was associated with a Paijan-like projectile point (see discussion below). With exception of the ^14^C assay in stratum MV-4, all other radiocarbon dates were derived from the deeper stratum MV-7, which dates from ~15,000 to 45,000 years ago from the top of its upper level to its base (see Fig 5 and Text G in S1 File). Two assays were from the MV-7 stratum in site CH-I: a date of 14,175–14,025 cal BP was assayed on wood charcoal from the buried surface of stratum MV-7 in Unit 5 and associated with a percussion flake; and a previous date of 15,058–14,034 cal BP was processed on wood charcoal in the upper level of stratum MV-7 in Unit 5 and also associated with percussion flakes [13,14]. Both the prior and recent dates from CH-I agree stratigraphically and chronologically with those from the MV-II site [13,14,25,34,35]. Six recently processed dates were from the upper to lower middle levels of stratum MV-7 in different excavation units of the MV-I site: 13,940–13,560 cal BP was assayed on collagen in an unburned bone fragment from a burned feature (No. 4) embedded in the buried surface of stratum MV-7 in Unit 45(A) and associated with percussion flakes; 14,590–14,064 cal BP was processed on a burned bone fragment associated with percussion flakes (Fig 3a) and an exotic manuport (Fig 6a) in a feature (No. 3) in the upper level of stratum MV-7 in Unit 56; 15,680–15,259 cal BP was assayed on a scorched bone in a burned feature (No. 5) in the lower upper level of stratum MV-7 in Unit 82 and also associated with percussion flakes; 16,520–15,850 cal BP was assayed on a burned plant stem in the upper middle level of stratum MV-7 in a feature (No. 8), Unit 55, and associated with flake spalls, percussion flakes, and exotic clay; 18,578–18,304 cal BP was processed on a burned plant stem in a burned feature (No. 10) in the lower middle level of stratum MV-7 in Unit 57 and also associated with percussion flakes; and 19,485–19,150 cal BP was assayed on an unburned bone fragment in a burned feature (No. 2) in the lower middle level of stratum of MV-7, Unit 45(A) and associated with percussion flakes (see precise contexts of ^14^C dates in Table 1). It is significant that all new radiocarbon assays date specific features with associated cultural materials and agree stratigraphically with the prior radiocarbon dates on cultural and non-cultural levels in the MV-I site [14,35] (see Table 1). Six additional samples of bone and sediments from three burned and unburned features in Units 45(A), 55, 56, 57 and 82 at MV-I were submitted for radiocarbon dating (i.e., Beta-369124, 369126, 371801, 371802, 371803, 371805), but none contained a sufficient quantity of bone collagen or of *in situ* flecks of charcoal for dating. (Scattered flecks were recovered beyond the outer edges of these features, but they were not dated because they were not securely embedded in the formal structure and sediment of the features.) We had expected these samples to date roughly between 18,000 and 14,500 cal BP, given their varying depths between 1.12 and 1.45 m.

**Table 1:** Radiocarbon dates from archaeological horizons and non-archaeological layers in the MV-I and CH-I and CH-II sites*.

Sample No.	Provenience	Depth/ Stratum (m)	13C/12C	Conventional Radiocarbon	68.2% calibrated Age Range (BP)	98.4% calibrated Age Range (BP)	Material
BETA-343109	CH-1, Unit 6	0.51, base of MV-4	-24.9	9320±40 BP	10407–10558	10660–10620	Burned plant stem
PRI-15-036-1	MV-1, Unit 45(A)	0.94, buried surface of MV-7	-21.42	11959±33 BP	13800–13630	13940–13560	Bone collagen
BETA-375837	CH-1, Unit 5	1.04, buried surface of MV-7	-29.5	12210±40 BP	13989–14125	14025–14175	Wood Charcoal
BETA-403545	MV-1, Unit 56	1.10, upper level of MV-7	-22.2	12350±40 BP	14110–14342	14064–14590	Bone collagen
BETA-65842	CH-1, Unit 5	1.21, upper level of MV-7	-29.3	12420±130 BP	14153–14727	14034–15058	Wood charcoal
BETA-375838	MV-1, Unit 82	1.31, lower upper level of MV-7	-22.4	12980±40 BP	15334–15565	15259–15680	Bone collagen
BETA-369125	MV-1, Unit 55	1.36, upper middle level of MV-7	-25.2	13200±60 BP	15694–15927	15850–16520	Burned plant stem
BETA-372893^#^	MV-1, Unit 57	1.45, lower middle level of MV-7	-24.3	15210±30 BP	18365–18503	18304–18578	Burned plant stem
BETA-372889	MV-1, Unit-45(A)	1.49, lower middle level of MV-7	-24.8	16000±60 BP	19138–19378	19150–19485	Bone collagen
AA75323	MV-I-T4	1.49, basal, middle level	-27.6	18910±300	22420–23080	22100–23515	Wood charcoal
BETA-^35193	Test Pit 42	1.63, upper lower level of MV-7	-------	23660±320 BP	27480–28001	27276–28405	Peat ball
BETA-^41983	Test Pit 42	1.69, upper lower level of MV-7	-------	27860±2010 BP	29969–33925	27844–35703	Peat ball
BETA-^7825	Test Pit 5	1.74, lower level of MV-7	-------	>33020 BP	-------	-------	Carbonized wood
BETA-^6754	Test Pit 44	1.76, lower level of MV-7	-------	33370±530 BP	36870–38305	36233–38758	Carbonized wood
BETA-52011^	Test Pit 42	1.97, basal level of MV-7	-------	>42100 BP	-------	-------	Unburned wood
BETA-373942^	Test Pit 57	-2.03, basal level of MV-7	-21.9	>43,500 BP	-------	-------	Animal Skin

*all dates calibrated with CALIB v7.0 using the Southern Hemisphere calibration curve [38,39].

^#^ This date is slightly deeper because it is from a unit farther downslope from other units.

^ ^14^C dates from non-cultural levels in stratum MV-7 in site MV-I.

A dubious association of one percussion flake of basalt, another flake, and a non-feature scatter of charcoal was recorded at a deeper level (1.47–1.49 m) in a downslope extension of Unit 56, which was sealed by overlying strata MV-1 to MV-4 (see Fig 5 and Text B in S1 File). The charcoal dated between ~23,515 and 22,100 cal BP. In addition, a white quartz chopper, two spherical stones, and an unknapped flake were recovered in Unit 55, the upper lower level of stratum MV-7, site MV-1 (Text A in S1 File, see S6 and S7i Figs). These lithics were not associated with a feature and datable material. Previously, peat balls at the 1.63 and 1.69 m in the upper lower levels in MV-I were ^14^C dated between ~23,000 and 29,000 BP (see Table 1). The middle lower to basal levels of stratum MV-7 are dated ~33,000 BP by ^14^C dates on wood charcoal associated with burned features and greater than 40,000 years ago by OSL assays (see Text G in S1 File) [13,14]. A fragment of animal skin in a peat ball at the 2.03 m basal level in Unit 57 of the MV-I site recently ^14^C dated at 43,500 BP. This date generally agrees with a measure of 42,100 BP on a previously ^14^C assayed peat ball at the 1.97 m non-cultural basal level in Test Pit 42 of MV-1 [14]. The data from these deeper levels are still too meager and inconclusive to determine whether they represent human activity or indeterminate natural features. At present, the latter case is perhaps more feasible, given that there is presently no convincing archaeological or other data to substantiate a human presence in South America prior to 20,000 years ago.

All charcoal dates were derived from burned plant stems and single pieces of wood. An attempt was made to identify the species of the stems but to no avail due to their small size. However, the stems are likely from small bushes or edible plant remains (e.g., Myrtaceae, *Typha*) reported in the pollen and phytolith section below (see Text F in S1 File). The dated wood charcoals were identified as relatively short-lived *Nothofagus* sp. trees (50–90 years, probably *N*. *dombeyi* or *coigue*), which are local to the area in the past and today. Dating old wood at sites in the Monte Verde area is not a problem for two reasons. First, an abundant supply and wide variety of trees were always available during the time periods discussed here. And second, wood rots quickly in the humid setting of the Northern Patagonian and Temperate Rainforest environments, making it culturally useless after a few years. Only the lifespan of the trees, which is relatively short in this case, can be contributed to old wood problems.

OSL dating aimed to improve the chronology of the stratigraphic units of the Monte Verde sites (see S2 Fig and Text G in S1 File). All luminescence dated samples were taken from a profile section in Unit 45(A) in the MV-I site, from profile sections in test pits in the MV-II site, and from the present-day bank of the Chinchihuapi Creek. Unit 45(A) was selected for sampling in the MV-I locality because it was the deepest excavation unit (~2.7 m) and it yielded intermittently occurring archaeological horizons throughout the previously ^14^C dated MV-7 stratum [14,34,35]. As discussed above and demonstrated by tephra, micromorphological, magnetic, and luminescence studies (Texts C-E and Text G in S1 File), there is no bioturbation or other disturbances in any of the excavated units, which might have affected the integrity of the luminescence ages.

The deposits selected for luminescence dating were mainly composed of poorly to moderately well-sorted, medium to very coarse subangular sand with occasional thin lenses of small gravel. Planar cross-bedded and parallel stratified ~30 cm thick sand bodies are common, usually capped by well-rounded, one to two clast-thick small pebble (0.5–1.2 cm in diameter) layers. Some sand beds show soft-sediment deformation structures, but these were avoided for luminescence sampling. The sands are composed of quartz, feldspar and lithic (polymineralic) grains. The luminescence dating of the Monte Verde sediments was based on the Single-Aliquot Regenerative (SAR) dose protocol [40] (see Text G in S1 File) applied to concentrates of quartz and K-rich feldspar in the 180–250 μm grain size. High residual signals hindered the use of the post-IRIR signal [40–42] for dating of the Monte Verde sediments. The optically stimulated luminescence of quartz and infrared stimulated luminescence (IRSL) of K-rich feldspar concentrates were the most feasible signals for dating the Monte Verde sediments. The K-rich feldspar IRSL ages with correction for athermal fading are compatible with quartz OSL ages and ^14^C ages of correlated stratigraphic units. Thus, K-rich feldspar IRSL, quartz OSL and ^14^C ages were integrated to define the chronology of sediment and archaeological depositions in the sites. Detailed explanations of the luminescence dating methods and results are in the Text G in S1 File.

The OSL dates suggest that sediment accumulation of stratum MV-7 in the MV-I and MV-II sites occurred primarily between 30,000 and 15,000 years ago. The most significant deviations occurred around 30,000 to 20,000 years ago, correlating roughly with the second Llanquihue glacial advance reported for this period [31]. The OSL dates generally agree with the radiocarbon ages for the upper to upper lower levels of the MV-7 stratum in site MV-1, ranging from ~15,000 to 25,000 years ago, where the recent archaeological horizons were recovered and where prior work recorded earlier cultural materials (see S2 Fig and Texts B-G in S1 File for discussion of the chronology and stratigraphy of the OSL and ^14^C assays.).

The OSL dates also confirm the prior ^14^C dating of cultural materials in the single component occupation of the MV-II site which was assayed at ~14,500 cal BP. The cultural materials rested on and were embedded in the buried surface of the MV-7 stratum, which was covered and sealed by the overlying non-cultural MV-5 peat layer. The OSL dates place the base of the younger, non-cultural MV-4 stratum (Unit C, see Text G in S1 File) at 10,174±883 years. This stratum directly overlies stratum MV-5, which has an OSL assay of 13,793±906 years. In turn, stratum MV-5 overlies and seals the MV-II archaeological component, which is embedded in the buried surface of stratum MV-7, the ancient terrace of the Chinchihuapi Creek. The surface layer of MV-7 was dated ~15,000 years ago by OSL means. Thus, the sequence of OSL assays in both the MV-I and MV-II sites not only correctly places the stratigraphic location of the MV-II occupation around 14,500 years ago [14,34,35], but also indicates that any cultural materials buried below the upper level of stratum MV-7 across the sandur plain date earlier than 15,000 years ago.

In summary, the combined twenty-seven previous and new radiocarbon assays and OSL ages (Text G in S1 File) are generally in chronological and stratigraphic order in the MV-I and CH-I sites. The most conservative estimate of the ages of the sporadic occupations on the sandur plain in these sites range between ~19,000 and 14,000 cal BP, on the basis of the minimum age represented by each of the ^14^C and OSL dates.

## Material Remains

### Lithic assemblages

The present lithic sample of thirty-nine stones is a small but significant addition to the previously reported MV-I and MV-II assemblages particularly as regards the added contextual and chronometric evidence. Percussion flaking of both local and exotic stones is well represented throughout the temporal span of the new evidence. Expediency in material selection and in the limited modification of individual pieces prevails as it did in the previously reported MV-I and most of the MV-II assemblages [30,43]. From a behavioral perspective, the people leaving these artifacts at sites MV-I, CH-I and CH-II were not solely dependent on local material sources (65.7% of the assemblage is of locally available stone and 34.3% is non-local; see S1 and S2 Tables in Text A in S1 File).

There is not a robust protocol for the analysis of most of the stone artifact forms from these sites, particularly the issue of distinguishing cultural from natural objects. Specimens are described from visual attributes of form, condition, and lithology (specifically facets and their relative “freshness,” edge attrition, shape, and any indication on how the present shape was acquired). Size is indicated by measurement of the three primary axes in millimeters using sliding calipers. Central to this analysis is comparison of stone morphologies and sizes to naturally occurring forms in the geologic deposits in and near the sites as reported previously; the local deposits occur as fluvioglacial and airborne volcanic clastic fill in a deep graben and include no bedrock outcrops, so they offer limited choices for tool stones. Stones are considered to be exotic manuports if their geologic sources are outside of the sandur plain and the project area.

**Fig 7:**
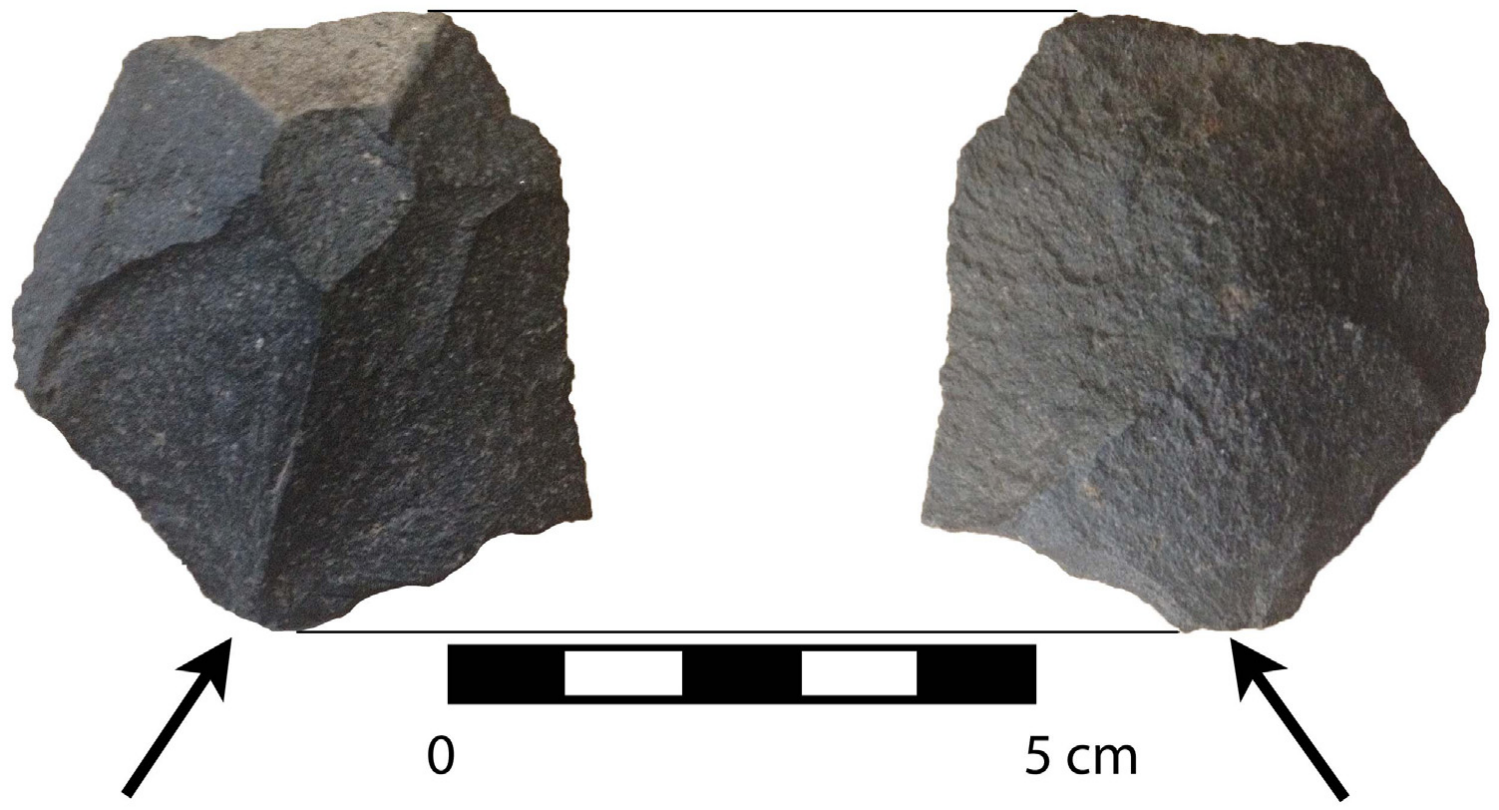
Serpentine pebble tool from Unit 17, MV-I, showing bifacially knapped and retouched edge. Serpentine is a raw material available in the coastal cordillera west of Monte Verde.

**Fig 8:**
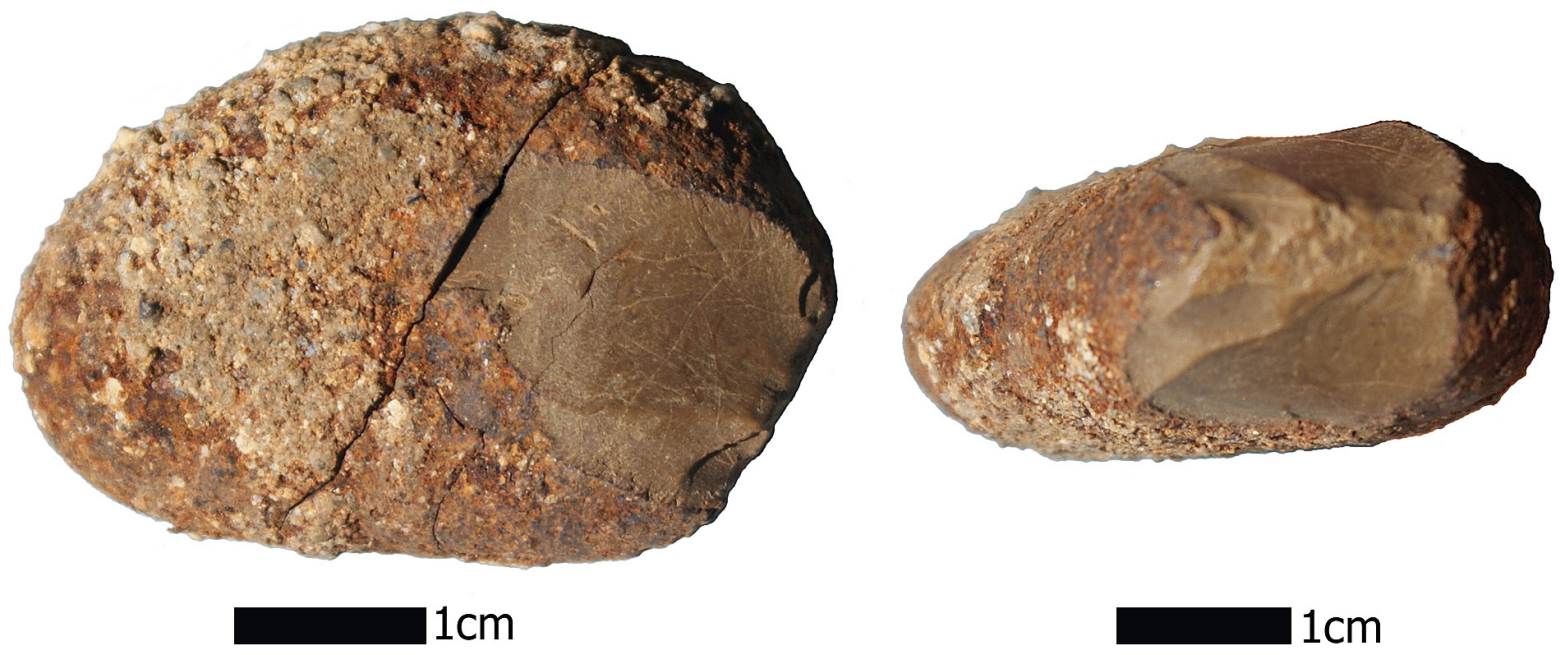
Basalt wedge showing seven facets on obverse face (one of which is cortex) and three on the reverse face. Arrows indicate inferred direction of force and point of impact of the blow that detached this piece.

Four stone artifact assemblages are distributed through time and across the investigated area (Fig 6; see Text A in S1 File, S6 and S7 Figs). The artifacts in these assemblages are closely similar to those previously reported as two assemblages, MV-I and MV-II, but the present evidence suggests that a more complex and prolonged cultural history transpired in this locality [30,43]. The earliest probable lithics (see S6 and S7i Figs), probably date around or before 25,000 years ago, are similar with the previously recorded possible site of MV-I [14,30] and consists of four specimens including two well-rounded possible “sling stones” (S6c-d Fig; sphericity indices of 0.699 and 0.690), a possible chopper with a clear percussion flake on an exotic white quartz (S6a and S7i Figs), and an angular spall of basalt showing no evidence of cultural use (S6b Fig). Next is nine specimens, dated between ~19,000 and 17,000 cal BP, including a clearly flaked pebble of serpentine (Fig 7), five human-made percussion flakes (three of basalt and two of unsourced limestone likely from central Chile or western Argentina (Fig 6e), a round stone, and a spherical stone possibly used as a sling stone (sphericity index of 0.692), and an exotic discoidal beach pebble manuport (Fig 6g). Dating between ~16,000- and 15,000 cal BP are three artifacts, a basalt wedge (Fig 8), a culturally produced basalt flake with clear percussion marks, and an intentionally split pebble with heavy marginal retouch and “edge-batter”. The fourth assemblage, dated at ~15,000 to 14,500 cal BP, is contemporary with MV-II [14,43] and numbers 18 specimens. This assemblage is comprised of a clear culturally produced core of unknown raw material (Fig 6h and S6c Fig show the reverse multi-flaked side), five human-struck percussion flakes of basalt (e.g., Fig 6b, 6f, 6i and 6k), eight pieces of percussion shattered basalt, three of which conjoin (Fig 6j) and attest to the intactness of the cultural deposit containing them, one split pebble with a retouched edge (Fig 6b, S6e Fig), two single faceted pebbles without microscopic evidence of cultural use, a wedge, and a multifaceted, percussion struck pebble without evidence for use (Fig 6d).

**Fig 9:**
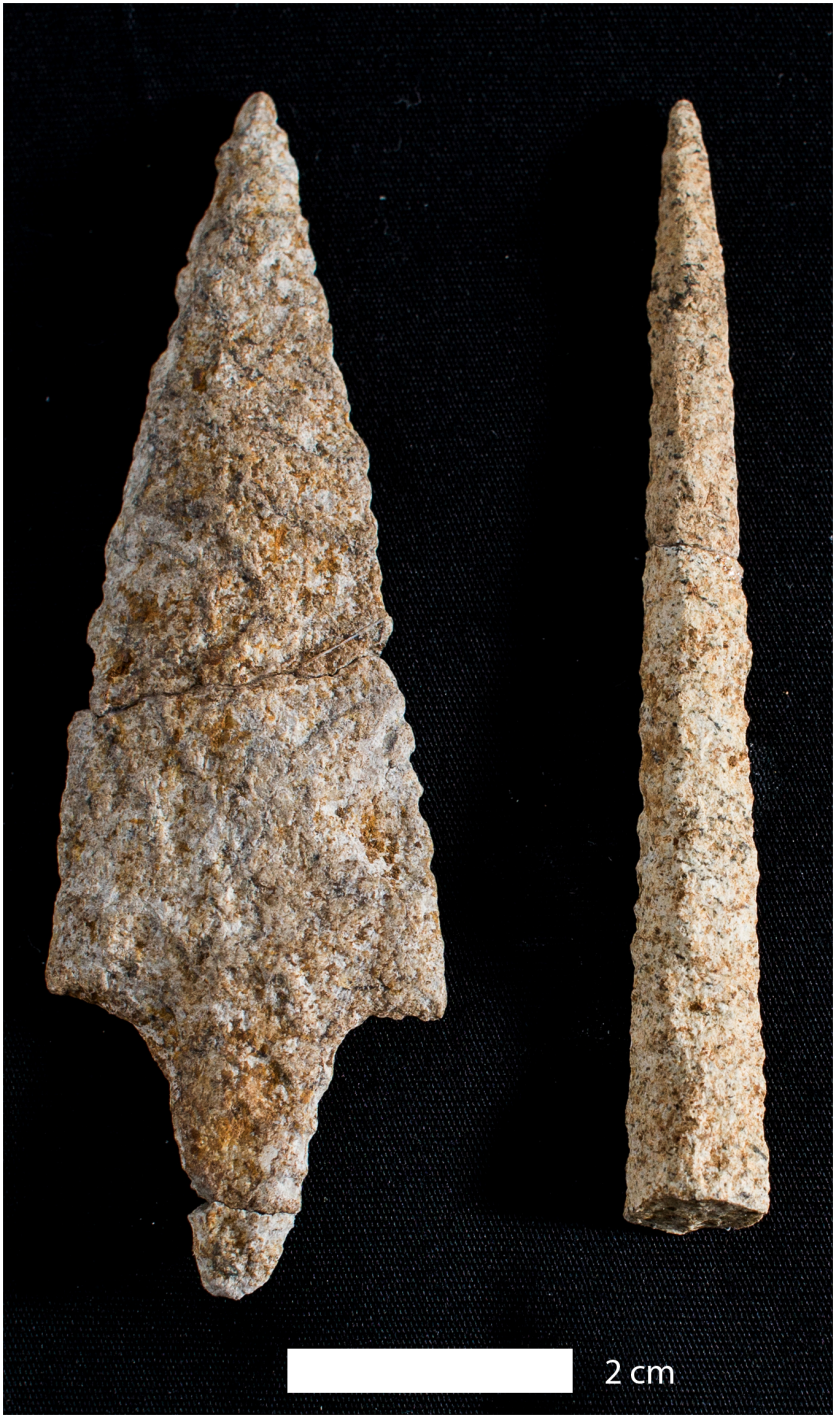
Paijan-like projectile point of rhyolite recovered from a late Pleistocene level at the CH-I site (Unit 6, Level 51 cm; see S6a Fig). A drill fragment is from the same level (right side).

Evidence for clear human-derived percussion flaking appears in all four assemblages and occurs primarily as local basalts and andesites, but the exotic serpentine (Fig 8), unsourced limestones, and white quartzite were also knapped. The beach pebble manuports also are exotic. For the most part, the artifacts lack evidence for wind or water erosion and display sharply defined flake scars and edges suggesting rapid burial, a condition also reflected by the associated burned features and microstratigraphic and sedimentological studies (see Texts C-E in S1 File). The first possible cultural assemblage, dated prior to ~20,000 cal BP, adds the presence of two possible sling stones to what was previously known about the early, possible MV-I site. In the second assemblage, ~19,000–17,000 cal BP, are clear human-produced flakes, a possible sling stone, and an exotic manuport in common with similar ones from the later MV-II. A flake, a wedge, and an edge-battered pebble in the third assemblage, ~16,000–15,000 cal BP are traits also shared with MV-II as are the core, flakes, and retouched split pebble in the fourth assemblage; the basalt wedge lacks any known counterparts. More than thirty small pieces of lithic debitage (ranging from 0.3–0.7 mm in size) were also recovered in and around several burned features. As an assemblage, the later 14,500 cal BP MV-II stands apart from all of the rest in its diversity and its apparent result from a relatively longer interval of occupation. Among the 2013 as well as the previously reported MV-II stone artifacts, exotic materials suggest a high degree of long-distance mobility or exchange. The manuports include discoid pebbles of quartz and quartzite not found in the Monte Verde area. Furthermore, there is no stratigraphic, taphonomic (e.g., animal burrowing, rootlet channels) or other evidence to suggests that the lithics dating prior to ~15,000 years ago in site MV-I were derived from earlier overlying levels. As noted earlier, the lithics in these deeper levels are directly associated with intact features and lay flat in the archaeological horizon. Lastly, recovered from the younger basal level of stratum MV-4 and its interface with the overlying stratum MV-7 in site CH-I (strata MV-5 and MV-6 are associated with the Chinchihuapi Creek drainage and thus are absent in the higher, older terrace where sites MV-I, CH-I and CH-II are located) are a Paijan-like projectile point and a hearth with a contracted stem and slight shoulder barbs (Fig 9 and S7a Fig) dated at 10,650–10,620 cal BP. This date agrees with radiocarbon assayed late Paijan points documented throughout the Andean region [44]. Also excavated from this same level was a drill fragment (Fig 9).

In addition, three grinding stones were recovered from test pits at the MV-II site. Sediments from these stones yielded pollen and phytolith remains from a wide variety of edible and non-edible species, supporting prior evidence dated ~14,500 cal BP of people grinding seeds and nuts from various economically useful plants [14], including *Gevuina*, *Lomatia*, Cheno-ams, Cyperaceae, *Polygonum*, Brassicaceae, *Cleome*, *Plantago*, and *Typha*.

With the exception of the possible manuport sling stones, most stone tools seem to have been used for scraping and cutting, as suggested by their morphology and by preliminary micro-usewear analysis (see Text H in S1 File). Although the majority of materials are similar to previous findings at MV-I and MV-II, the technological diversity presented here likely represents the variable focus on shaping and knapping production, which may be indicative of the raw material economy. This variation may represent adaptations that are the earliest precursors to the later production techniques recorded at the MV-II site.

At the older ~33,000 cal BP depth in the basal levels of stratum MV-7 in site MV-I, no new burned features and datable radiocarbon material were associated with the recently recovered materials, although OSL assays from the stratum containing the four lithics date ~25,000 cal BP. Our current material inventory for the older, deeper possible cultural MV-I levels is still very small (now a total of 30 stones and 3 possible cultural features) and includes forms such as unmodified and retouched flakes, other elongated flakes, two possible hammerstones, and unworked but possibly used manuports. There still is no taphonomic evidence to explain the presence of these deeper materials (e.g., tree roots, animal burrows). The new findings do not improve the resolution of these older materials, and we remain inconclusive and skeptical about their cultural status [14,30]. However, the recent work confirms the discrete and dispersed nature of these materials in the older, deeper levels of stratum MV-7 at the MV-I site and the very remote possibility that people were in the area earlier than we have documented here.

Lastly, if the early archaeological horizons documented here were associated with transient peoples primarily utilizing unifacial stone tools, then the expedient pebble tool industry and the high percentage of exotic raw material make sense. Pebble tool industries with a working edge are relatively common in early multi-purpose technologies throughout South America [5,45,46]. These industries are dominated by a range of shaped edge-trimmed flakes and edge-battered pebbles and cobblestones and are assumed to represent, primarily if not entirely, expedient technologies [5,7,14,16, 23,28,45,46]. As noted earlier, not well understood in South America is the historical and technological relationship between early unifacial and bifacial assemblages and whether one preceded the other or whether they represent distinctive adaptations associated with different migrations into different environments. At present, the stone tool assemblages from several early sites in South America generally indicate independent technological developments once people entered the continent [5,7,12,14–16,22,23,28–30, 45,46], with antecedent knowledge probably derived from an older stone tool industry likely shared with contemporary North American culture.

### Burned features

Twelve burned features dated between ~14,000 and at least 18,500 cal BP were scattered mainly in the MV-I site area between the depths of 0.95 and 1.49 m in the upper middle to upper lower levels of stratum MV-7 (Fig 3; see S4 Fig and Table 2 for the contents of each feature). They do not contain clay-lined pits and burned rocks, yet they are directly associated with fragmented burned and unburned animal bones, knapped lithics, and varying amounts of primarily ash and secondarily charcoal. No non-cultural or other burned or feature-like areas containing charcoal and ash were recovered from the numerous cores, trenches, and units excavated in sites. The features were documented in five of the seven excavated units in MV-I and in one of two units in CH-I. Five features were associated with Unit 45(A), two in Unit 56, and one each in Units 55, 57 and 82 in site MV-I; two were recovered from Unit 5 in CH-I. The features measured between ~1.0 and 2.8 cm in thickness and varied in form from amorphous to semi-circular, roughly averaging ~33 by 42 cm in size, excluding light scatters of ash and charcoal flecks. All features were stratigraphically embedded in thin horizontal lenses at different levels between ~.25 and .80 cm above the three clay-lined burned features previously recovered in the deeper levels of stratum MV-7 in the MV-I site and dated ~33,000 BP [14].

**Table 2:** Distribution and Characteristics of Features Recovered during the 2013 Field Season.

Unit	Feature Number	Dimensions (Length, Width, Thickness in cm)	Content (A = ash; C-charcoal; L = lithic; B = bone)
**Site MV-I**			
45(A)	1	34 x 23 x 2.0	A, C, L
45(A)	2	17 x 16 x 1.0	A, C, L
45(A)	4	18 x 18 x 2.0	A, C, B
45(A)	7	27 x 23 x 2.2	A,C
45(a)	9	42 x 32 x 2.6	A,C,L
55	8	39 x 33 x 2.8	A,C,L
56	3	37 x 33 x 2.3	A,C,L,B
56	6	65 x 46 x 2.1	A,C,L,B
57	10	33 x 21 x 2.2	A,C,L
82	5	15 x 12 x 1.2	A,C,L,B
**Site CH-I**			
Unit 5	11	24 x 19 x 2.6	A,C,B
Unit 5	12	39 x 19 x 2.2	A,C,L

A part of one feature (No. 8) in Unit 55 at the 1.39 cm level of stratum MV-7 contained a percussion flake and an elongated and rounded block of exotic clay that measured 8.3 by 15.4 cm, (S4a Fig). No datable organic material was associated with this feature, but its relative depth and context suggests it dates between ~16,000 and 15,000 cal BP. The origin and nature of this peculiar block of clay is not known.

It is not known whether any of the recently discovered features dating between ~14,500 and 14,000 years ago were associated with the previously documented MV-II occupation or with different visits at different times by small groups of people.

### Faunal remains

Four burned, one slightly scorched, and four unburned bone fragments (1–3.5 cm long) were excavated at sites MV-I and CH-I. The majority of the fragments are too small to identify the species, although they are possibly from ghompothere and paleocamelid, the two mammals previously recovered at MV-II and CH-I [47]. Two bone fragments reveal comparably thicker epiphyses (~0.9 cm) that are more suggestive of a larger animal such as ghomphothere. One small bone fragment is the head of a tibia, probably from a deer or small horse (S5 Fig). Six of the nine bone fragments were recovered from five burned features that were associated directly with stone artifacts or located within a few centimeters at the same level (Fig 5). The fractured edges of two bones suggest possible flaking or crushing, but the pieces are too small to determine cause. None of the bones have cutmarks, trampling striations, or root scars. The direct association of economically useful plant remains (see Text F in S1 File), worked lithics, and bone fragments with the features suggest human intervention. An animal skin fragment was recovered from a peat ball at the interface between strata MV-7 and MV-8 at a depth of 2.1 m and dated at 43,500 calibrated years ago (Table 1). This date agrees with a previous ^14^C assay of 42,000 BP at the same level in a nearby excavation block [14,35]. No cultural materials were associated with the skin.

### Paleobotany

All cultural features and selected sediment samples from various levels in stratum MV-I were subjected to flotation, resulting in the recovery of no identifiable macro-botanical remains except for the burned stems and wood charcoal reported here [14]. Pollen, starch grain, and phytolith studies were carried out on five samples submitted from two burned features and from three grinding stones from sites MV-I and MV-II, respectively. These studies yielded evidence of probable food remains and the local environment during the time period under study.

Sample 3 represents fill from an ash/charcoal feature at site MV-I (Unit 82, No. 5) dated to ~15,680–15,259 cal BP. It displayed a large quantity of *Nothofagus* pollen, indicating local growth of Southern Beech. Poaceae pollen frequency was elevated in this sample, suggesting processing and possibly parching grass seeds. Small quantities of *Podocarpus*, High-spine Asteraceae, *Cirsium*, Liguliflorae, *Caltha*-type, *Cleome*, Cyperaceae, Polygonaceae, Rosaceae, and *Sphaeralcea* pollen indicate that local vegetation also included these plants. Liguliflorae, Caltha-type, Cyperaceae, and Polygonaceae pollen are indicators of wetland vegetation in the vicinity of the site. Only a few fern spores of two types were observed in this sample. The phytolith record confirms a grassy wetland environment.

Sample 4 was collected from a burned feature (Unit 57, No. 10) dated to ~18,578–18,304 cal BP. This pollen signature was dominated by Liguliflorae pollen, suggesting an open weedy area with trees. Small quantities of Myrtaceae, *Nothofagus*, *Podocarpus*, Apiaceae, *Artemisia*, High-spine Asteraceae, Brassicaceae, Cyperaceae, *Plantago*, Poaceae, *Megalachne* grass, *Polygonum*, *Rumex*, and *Typha* angustifolia-type pollen document trees and shrubs growing in the area. Fern spores were observed, but were not particularly abundant. The phytolith record presents a similar profile.

Recovery of Polygonum pollen from the three grinding stone samples from the MV-II site, dated ~14,500 cal BP, suggests processing a member of this genus. Likewise, recovery of *Rumex* pollen from one grinding stone suggests processing an edible portion of the plant during this occupation. It also is possible that some of the stones represented were used to grind seeds and perhaps nuts from a variety of plant remains that included nuts from *Gevuina* and *Lomatia* and seeds from Cheno-ams, Cyperaceae, *Polygonum*, *Rumex* and *Typha*.

Diatoms, spherasters, and/or sponge spicules were observed in samples from a burned feature in MV-I and especially one of the grinding stones from MV-II. The two burned samples yielded small quantities of diatoms and the occasional spheraster and sponge spicule, which suggests that the sediments were not overly wet, perhaps indicating their use during the summer conditions sometime after 15,000 cal BP.

## Discussion

Paleovegetation records in the study area suggest that climate warming occurred between ~26,000 and 18,400 cal BP, with short cooling events at ~25,100 and 18,100 cal BP. Warming after the last glacial termination was well underway by ~17,600 cal BP and was followed by warmer pulses at ~17,000 and 15,300 cal BP. Post-glacial conditions changed to a cool, temperate climate by ~15,000 cal BP, slightly cooler and wetter than today [31–33,48], a time when the Chinchihuapi Creek formed. These intermittent warming trends correspond with most of the archaeological horizons recorded in sites MV-I, CH-1 and CH-II between ~18,500 and 15,000 cal BP and with the later, more prolonged presence of people at MV-II around ~14,500 cal BP. During all periods, volcanic eruptions and ecological barriers may have impacted people’s movements, as suggested by the numerous tephra lenses at sites and especially by a coating of ash on the artifacts at MV-II [14,34].

The new horizons represent low-density, discontinuous cultural deposits that contain few cultural materials. This suggests discrete, functionally similar, short-term anthropogenic activities most likely associated with hunting and gathering, heating food in small hearths, and producing and discarding expedient stone tools. More than a third of the stone tools associated with these deposits are made of exotic material, suggesting a high degree of mobility and/or long-distance exchange. If these scenarios are correct, then the settlement pattern in the Monte Verde area during this period was probably just one of several with small groups of people seasonally adapted to cold parkland and boreal environments, most likely during the warmer months. After ~17,000 cal BP, when the environment changed to a slightly warmer Northern Patagonian forest and after 15,000 cal BP, when the Chinchihuapi Creek formed, people settled for a longer stay as evidenced by more varied resources and artifact types and by the more extensive cultural deposits at MV-II.

We had postulated previously that the wide variety of local and non-local resources at MV-II [13,14,25], which were obtained from a mosaic of environmental zones extending from the western Argentina steppes to the Pacific coastline, suggests direct procurement by people at the site. Given the detailed knowledge required to exploit several highly specific species (including medicinal plants and seaweeds) from different and distant zones, we now consider an additional explanation to account for the presence of exotics at the site, that is, exchange with different groups of people dispersed across these zones. It does not seem feasible that people at MV-II would have had intimate knowledge of so many different resources spread over such a large area. This knowledge was most likely learned and shared by several groups actively involved in exchange networks by at least 14,500 cal BP.

Lastly, because few historical and present-day foragers have been documented in similar types of cold, post-glacial environments, it is difficult to use ethnographic analogy to gain insight into the possible types of archaeological records and inferred human behaviors we might expect for this earlier time period in south-central Chile. Perhaps one of the closest analogues is the Nunamiut of northern Alaska who made sporadic and brief hunting trips into cold, post-glacial-like environments, creating an ephemeral material record of small scattered hearths, bone remains, and other traces [49]. In comparison, there are several archaeological studies in periglacial and glaciogenic settings in high northern latitudes that discuss early pebble tool cultures and ephemeral, discontinuous archaeological records [50,51], which seem to be similar in some ways to the data presented here.

## Conclusion

An element that seems predictable in the archaeological horizons reported here is that the successive human presence in the Monte Verde area between at least 18,500 and 14,500 cal BP is likely to have been connected to Pacific coastal locations and possibly to deglaciated passes through the Andes to the Argentine steppes. This is suggested by the presence of exotic raw lithic materials dated to this period and plant species from both areas, especially those from coastal beaches and estuaries at MV-II [14,25]. It is doubtful whether local inhabitants would have procured non-local lithics when suitable basalts, andesites, quartzites, and other stones were readily available in the site area. The presence of a relatively high number of these materials prior to ~14,500 cal BP most likely suggests people from distant areas passing through the Monte Verde area.

In turning to a methodological issue, the new archaeological horizons in the Monte Verde area are difficult to trace laterally over areas larger than ~8–10 m^2^ and probably represent only fragments of a broader landscape utilized by people adapting to changing climates and environments in the area. More of these horizons surely exist in the site areas at different vertical and horizontal locations undiscovered by out recent investigation. If we had employed a different excavation strategy focused more on extended, large block excavations than on discontinuous, systematically-placed cores, test pits and limited block excavations, we probably would have obtained similar results. That is, more extensive horizon excavation also probably would have revealed the same discontinuous and ephemeral nature of the earlier archaeological record of MV-I and CH-I and CH-II sites, which we first documented in the early 1980s (13,14).

The criteria for defining and explaining some early sites may gradually change as we reconsider the scale of analysis applied by current research designs. For instance, when we first excavated the MV-II site in the late 1970s, we opened spatially limited areas (5 by 8 m and a few outlying 1 by 2 m test pits). These areas exposed one bifacial tool, a few unifacial flakes, and the bone remains of “mastodonts” or gomphotheres [24,47]. Later in the 1980s, when we returned to the site and excavated three larger excavation blocks (~6 by 15 m and more test pits and trenches), we discovered the dense and more extensive structured evidence of the MV-II occupation [13,14]. Now, after the recent investigation of a more extensive area and of other types of depositional environments in the site, we have discovered a different, more complex ecological and ephemeral archaeological setting. Would the type of evidence at and our thinking of previously some excavated early sites in other areas of the Americas change if they were reexamined and more extensively excavated?

Furthermore, the type of ephemeral records revealed at sites like MV-I and CH-I does not easily fit the criteria of more laterally and/or vertically dense cultural deposits evidenced at later sites, such as MV-II, Arroyo Seco in Argentina [52], Gault and Friedkin sites in Texas [9–10], Clovis and other early localities in North America [1,8,11]. These and especially the later Clovis and Fishtail sites may represent a time when landscape use had risen to the point of being more archaeologically visible as a result of human populations exploratory less and colonizing and settling in more. The discontinuous and minimal nature of earlier records and particularly those reported in Brazil [12,15,16, 22,23,28,53], Peru [45] and North America [8–9] challenge us to consider a wider variety of temporal, spatial and archaeological scales of early, possibly first arrival, human activity associated with sites of low archaeological visibility and with stone and bone technologies sometimes different from what we expect. The types of discontinuous and short-lived records reported here make the task of defining their archaeological and taphonomic characteristics and evaluating their scientific validity or invalidity more difficult than expected.

To conclude, the chronology and nature of the peopling of the New World are the focus of great deliberation between multiple schools of thought: some stress a short chronology and others a long chronology, some advocate one migration and others multiple migrations, some point to Asia as the only source of human entry and others point to Europe. For the moment, the majority of anatomical, archaeological and genetic evidence gives credence to the view that people were relatively recent arrivals to the Americas, probably sometime between 20,000 and 15,000 years ago. The current evidence presented here for the Monte Verde area best fits this scenario; however, this may change as more data are gathered and assessed. The early archaeological record of the Americas continues to be remarkably unpredictable and intriguingly complex.

## Supporting Information

S1 Fig View of present-day drainage setting with elevated areas and patchy vegetation. . This low-energy setting, situated near the modern-day glacial below the active volcano of Tronador located about 50 km east of Monte Verde, is topographically and ecologically reminiscent of the sandur plain at the site that was occupied in late Pleistocene times. Note the narrow and shallow trough drainages slightly eroded by seasonal rainwater and snowmelt and the intact, uneroded vegetated rises between them, the latter similar to those containing the archaeological horizons reported here in the buried sandur plain at Monte Verde.
(PDF)

S2 Fig Schematic profile of archaeological horizons and OSL dated samples in sites. a. Schematic profile of the location of archaeological horizons and OSL dated samples (Unit 45(A)) on uneroded, elevated areas between the narrow, shallow channels of the braided system in the buried sandur plain of the Monte Verde area; b-g. schematic profiles of the sedimentological and archaeological deposition phases for the study area; b. the two profiles, one from Unit 45(A) on the south side of the present-day Chinchihuapi Creek and the other from the MV-II site in the north bank terrace of the creek, correlate chronologically and sedimentologically. An OSL date reveals the MV-II occupation resting on the palimpsest surface of MV-7 stratum. Stratum MV-7 was deposited around 42,000 years ago and then exposed ~15,000 years ago when the creek formed (stratum MV-6 is the ancient creek bed). Around 15,000 years ago Chinchihuapi Creek downcut and washed away the upper and middle levels of stratum MV-7 in the north section (the ~40,000–14,000 year upper and middle layers of stratum MV-7 shown in section D of the profile of Unit 45(A) were not eroded). While the ~42,000 year surface was exposed ~15,000 years ago in the north section, the MV-II occupation took place shortly afterwards ~14,500 years ago and then sometime before 14,000 years ago both the exposed ~42,000 year surface of stratum MV-7 and the MV-II archaeological remains resting on it in the north section were buried by the subsequent deposition of strata MV-1 to MV-5. As indicated by the new evidence reported here, the uneroded surface and upper level of stratum MV-7 on the higher south side (Unit 45(A)) of the creek also were occupied between ~14,500 and 14,000 years ago; c-g. depositional and erosional Phases I-V schematically represent the sedimentological and archaeological sequence across site areas: c. Phase I: beginning before ~45,000 years ago the high terrace in site MV-I (Unit 45 (A)) was formed and comprised of intermittent shallow channels making up the braided stream system of the sandur plain. The channels roughly flowed from southeast to northwest; d. Phase II: after ~33,000 years ago the upper and middle levels of stratum MV-7, the high terrace levels as depicted in Unit 45 (A), were eroded in some places (curvy yellow line), then between 28,000 and 15,000 years ago new deposits of shallow braided stream channels were formed, including the airborne deposition of tephra lenses and the subsequent intermittent seasonal occupations by humans between 19,000 and 15,000 years ago. Sediment deposition during this phase was not continuous as indicated by non-depositional hiatuses; e. Phase III: ~15,000 years ago, deep lineal erosion occurred in some places and the Chinchihuapi Creek was formed (the date for the upper level of stratum MV-6, the creek bed, is shown in b,f and g), washing away the upper and middle levels of stratum MV-7 in the north section and exposing the surface of the ~42,223 year level of this stratum, upon which the MV-II occupation took place: f. Phase IV: a soil contemporary with the deposition of stratum MV-4 developed in the area of Unit 45(A) in the south section and strata MV-4 and MV-5 formed over and sealed the MV-II site in the north section; g. Phase V, strata MV-1 to MV-3 formed in both sections.
(PDF)

S3 Fig Thin tephra lenses in the past and present. a: arrows point to one of several thin, orange pumice lapilli lenses typical of those found throughout the MV-7 stratum containing the archaeological horizons; b: ventral side of a percussion flaked tool embedded in a 2 cm thick tephra lens located on an uneroded, elevated surface between narrow drainage channels in stratum MV-7, Unit 56 (see Fig 6b for a close-up of the dorsal side of the flake); c: typical micro-setting where thin orange lenses form during the summer months in shallow humid grassy areas at Monte Verde.
(PDF)

S4 Fig Archaeological features showing underlying tephra lens and burned area. a. Oval-shaped object of clay associated with a percussion struck lithic in Unit 55; b. Feature 9 in Unit 45(A) showing underlying orange tephra lens and overlying burned area with *in situ* patch of charcoal (bottom arrow) and percussion flake (top arrow).
(PDF)

S5 Fig Epiphysis of animal tibia. Fractured and partially burned epiphysis of a tibia probably of a deer or horse from Unit 56, MV-I dated at 13,940–13,560 cal BP (see Table 1).
(PDF)

S1 File Text A. Description of Lithics. Text B. Chrono-Stratigraphy of the 2013 Cultural Materials. Text C. Mineral, Petrographic and SEM Analyses of Tephra Sediments. Text D. Micromorphological Analysis of Archaeological Sediments. Text E. Magnetic Analysis of Archaeological Sediments. Text F. Pollen, Phytolith, and Starch Grain Analyses of Features. Text G. Optimal Luminescence Dating. Text H. Lithic Micro-Usewear Study.
(DOCX)
