# Correction: New Archaeological Evidence for an Early Human Presence at Monte Verde, Chile

**DOI:** 10.1371/journal.pone.0145471

**Published:** 2015-12-23

**Authors:** Tom D. Dillehay, Carlos Ocampo, José Saavedra, Andre Oliveira Sawakuchi, Rodrigo M. Vega, Mario Pino, Michael B. Collins, Linda Scott Cummings, Iván Arregui, Ximena S. Villagran, Gelvam A. Hartmann, Mauricio Mella, Andrea González, George Dix

The images for Figs 7 and 8 have been incorrectly swapped. Please view the correct Figs 7 and 8 here.

**Fig 7:**
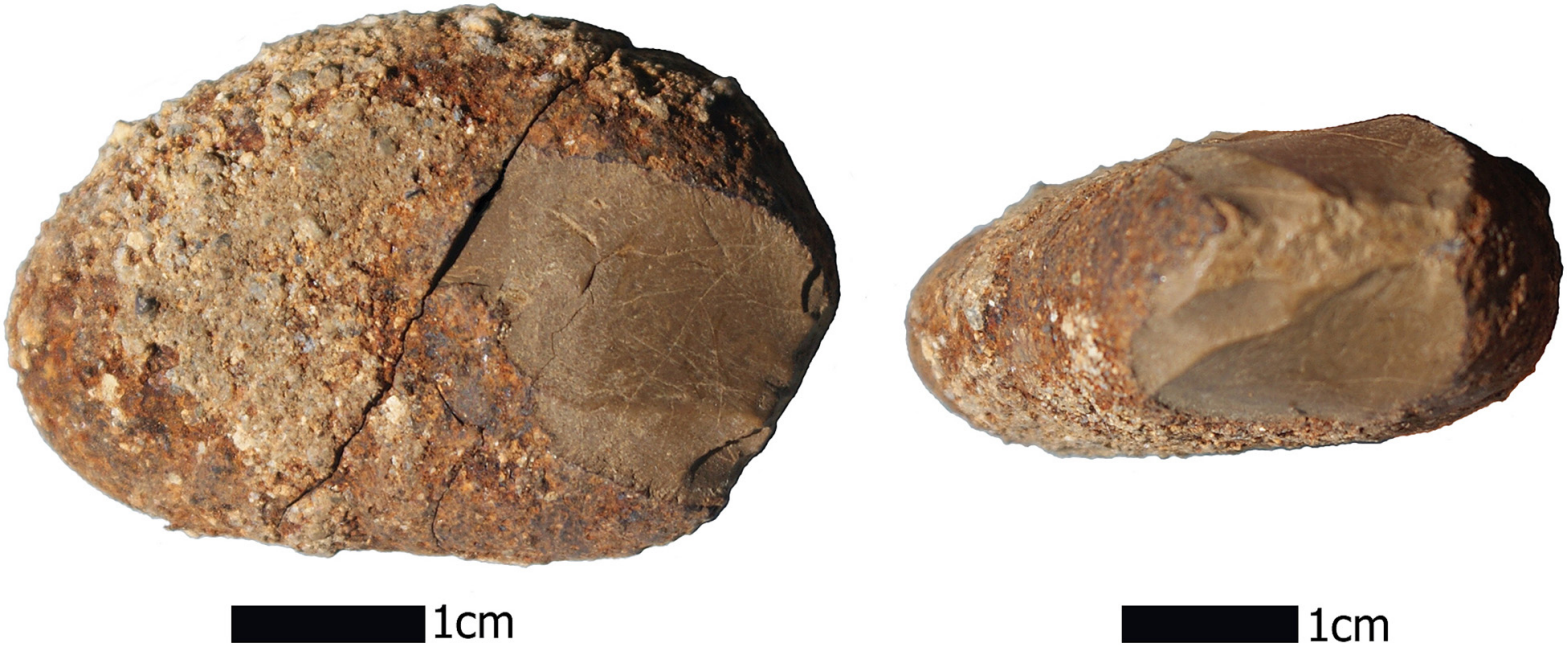
Serpentine pebble tool from Unit 17, MV-I, showing bifacially knapped and retouched edge. Serpentine is a raw material available in the coastal cordillera west of Monte Verde.

**Fig 8:**
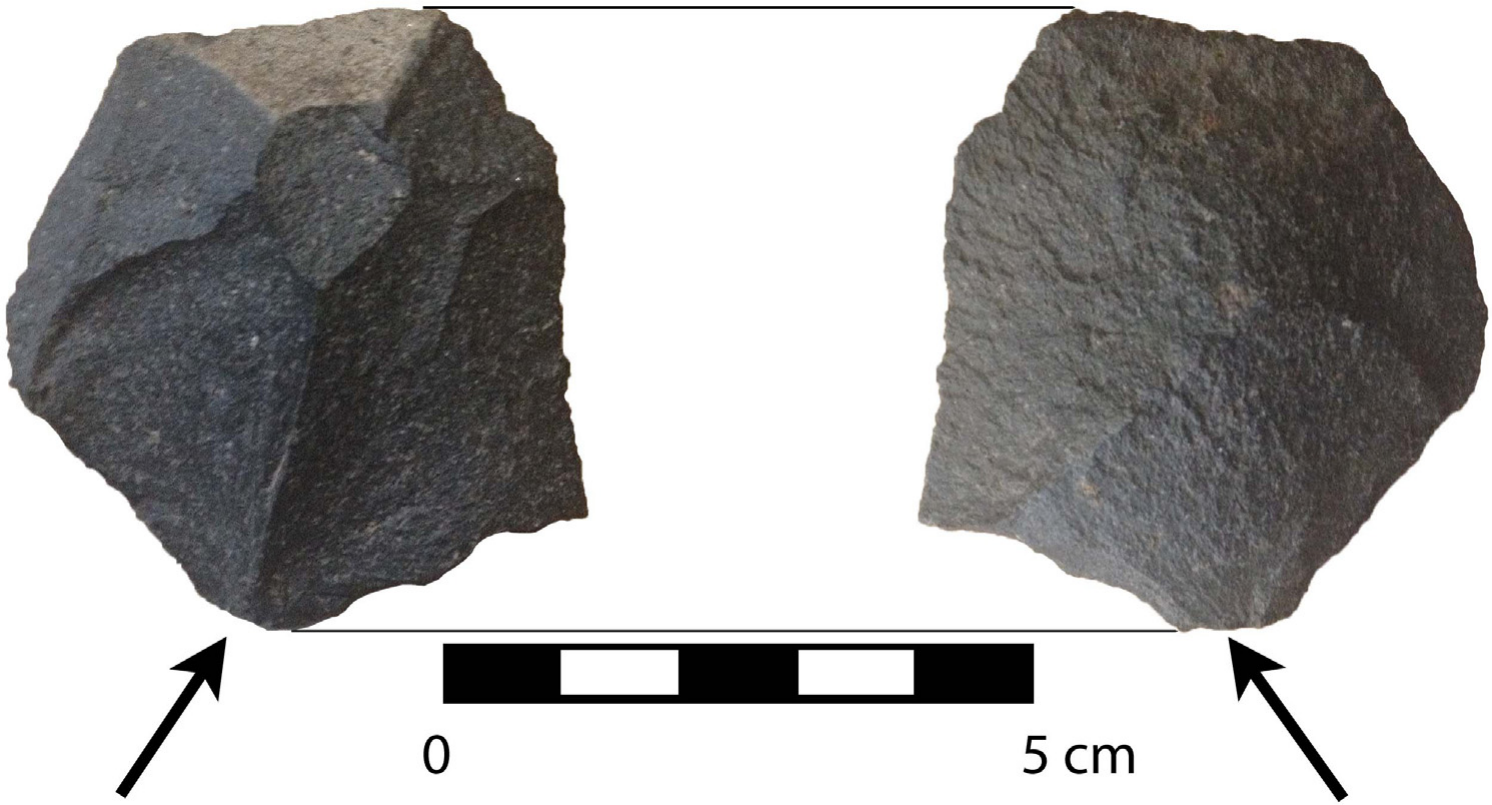
Basalt wedge showing seven facets on obverse face (one of which is cortex) and three on the reverse face. Arrows indicate inferred direction of force and point of impact of the blow that detached this piece.
