# Supplementary material for: New Archaeological Evidence for an Early Human Presence at Monte Verde, Chile
Source: PLoS One. 2015 Nov 18;10(11):e0141923. doi: 10.1371/journal.pone.0141923 (PMC4651426; doi:10.1371/journal.pone.0141923)
Supplement: S1 Fig — . This low-energy setting, situated near the modern-day glacial below the active volcano of Tronador located about 50 km east of Monte Verde, is topographically and ecologically reminiscent of the sandur plain at the site that was occupied in late Pleistocene times. Note the narrow and shallow trough drainages slightly eroded by seasonal rainwater and snowmelt and the intact, uneroded vegetated rises between them, the latter similar to those containing the archaeological horizons reported here in the buried sandur plain at Monte Verde. (PDF) [file pone.0141923.s001.pdf]

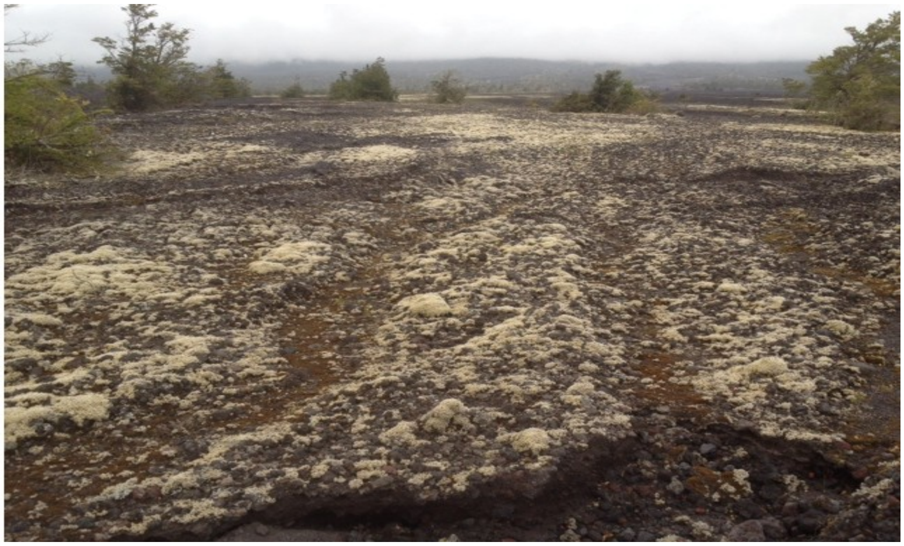

S1 Figure. View of a present-day shallow, braided drainage setting with slightly elevated areas and patchy vegetation. This low-energy setting, situated near the modern-day glacial below the active volcano of Tronador located about 50 km east of Monte Verde, is topographically and ecologically reminiscent of the sandur plain at the site that was occupied in late Pleistocene times. Note the narrow and shallow trough drainages slightly eroded by seasonal rainwater and snowmelt and the intact, uneroded vegetated rises between them, the latter similar to those containing the archaeological horizons reported here in the buried sandur plain at Monte Verde.
