# Supplementary material for: New Archaeological Evidence for an Early Human Presence at Monte Verde, Chile
Source: PLoS One. 2015 Nov 18;10(11):e0141923. doi: 10.1371/journal.pone.0141923 (PMC4651426; doi:10.1371/journal.pone.0141923)
Supplement: S2 Fig — a. Schematic profile of the location of archaeological horizons and OSL dated samples (Unit 45(A)) on uneroded, elevated areas between the narrow, shallow channels of the braided system in the buried sandur plain of the Monte Verde area; b-g. schematic profiles of the sedimentological and archaeological deposition phases for the study area; b. the two profiles, one from Unit 45(A) on the south side of the present-day Chinchihuapi Creek and the other from the MV-II site in the north bank terrace of the creek, correlate chronologically and sedimentologically. An OSL date reveals the MV-II occupation resting on the palimpsest surface of MV-7 stratum. Stratum MV-7 was deposited around 42,000 years ago and then exposed ~15,000 years ago when the creek formed (stratum MV-6 is the ancient creek bed). Around 15,000 years ago Chinchihuapi Creek downcut and washed away the upper and middle levels of stratum MV-7 in the north section (the ~40,000–14,000 year upper and middle layers of stratum MV-7 shown in section D of the profile of Unit 45(A) were not eroded). While the ~42,000 year surface was exposed ~15,000 years ago in the north section, the MV-II occupation took place shortly afterwards ~14,500 years ago and then sometime before 14,000 years ago both the exposed ~42,000 year surface of stratum MV-7 and the MV-II archaeological remains resting on it in the north section were buried by the subsequent deposition of strata MV-1 to MV-5. As indicated by the new evidence reported here, the uneroded surface and upper level of stratum MV-7 on the higher south side (Unit 45(A)) of the creek also were occupied between ~14,500 and 14,000 years ago; c-g. depositional and erosional Phases I-V schematically represent the sedimentological and archaeological sequence across site areas: c. Phase I: beginning before ~45,000 years ago the high terrace in site MV-I (Unit 45 (A)) was formed and comprised of intermittent shallow channels making up the braided stream system of the sandur plai [file pone.0141923.s002.pdf]

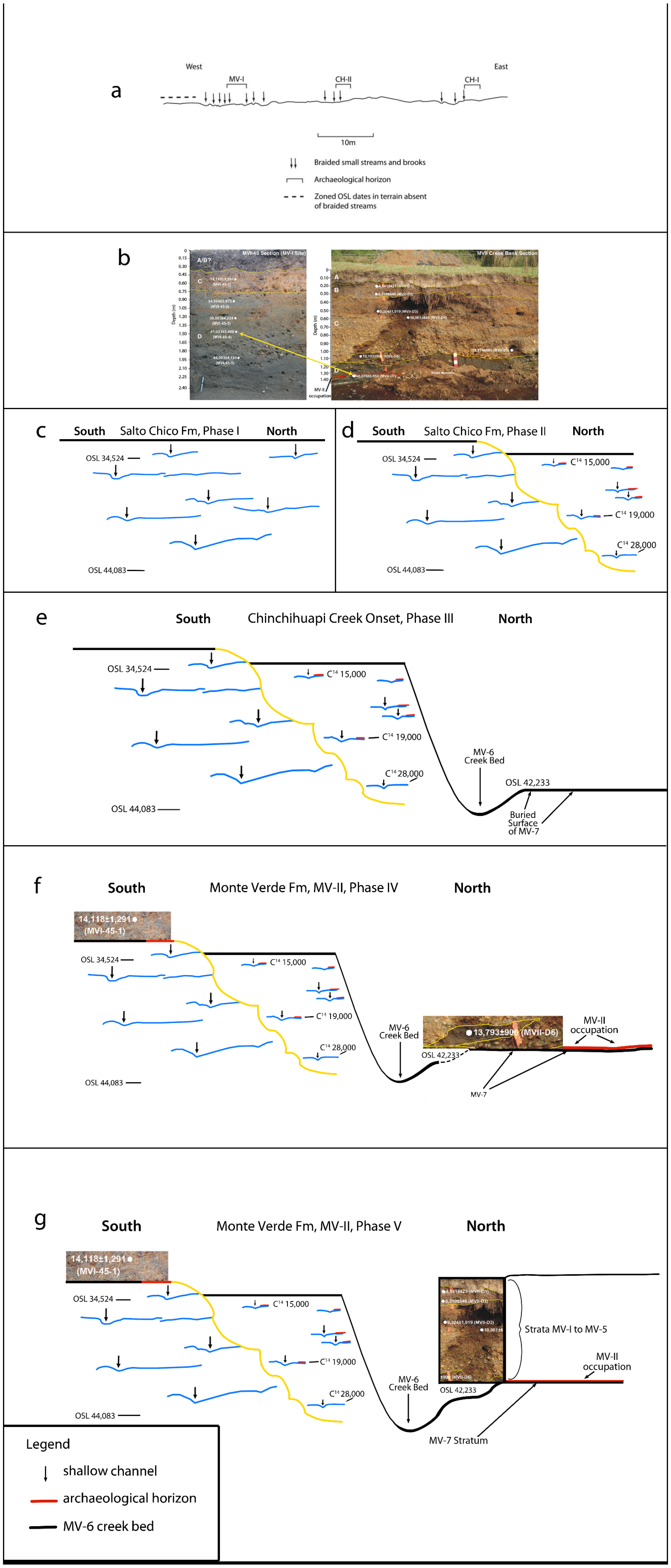
a
West
East
MV-I
CH-II
CH-I
10m
Braided small streams and brooks
Archaeological horizon
Zoned OSL dates in terrain absent of braided streams
b
MVI-45 Section (MV-I Site)
MVII Creek Bank Section
c
South
Salto Chico Fm, Phase I
North
OSL 34,524
OSL 44,083
d
South
Salto Chico Fm, Phase II
North
OSL 34,524
C14 15,000
C14 19,000
C14 28,000
OSL 44,083
e
South
Chinchihuapi Creek Onset, Phase III
North
OSL 34,524
C14 15,000
C14 19,000
C14 28,000
MV-6 Creek Bed
OSL 42,233
Buried Surface of MV-7
OSL 44,083
f
South
Monte Verde Fm, MV-II, Phase IV
North
14,118±1,291
(MVI-45-1)
OSL 34,524
C14 15,000
C14 19,000
C14 28,000
MV-6 Creek Bed
13,793±900 (MVII-D6)
MV-II occupation
OSL 42,233
MV-7
OSL 44,083
g
South
Monte Verde Fm, MV-II, Phase V
North
14,118±1,291
(MVI-45-1)
OSL 34,524
C14 15,000
C14 19,000
C14 28,000
Strata MV-I to MV-5
MV-II occupation
MV-6 Creek Bed
OSL 42,233
MV-7 Stratum
OSL 44,083
Legend
shallow channel
archaeological horizon
MV-6 creek bed

S2 Figure. a. Schematic profile of the location of archaeological horizons and OSL dated samples (Unit 45(A)) on uneroded, elevated areas between the narrow, shallow channels of the braided system in the buried sandur plain of the Monte Verde area; b-g. schematic profiles of the sedimentological and archaeological deposition phases for the study area; b. the two profiles, one from Unit 45(A) on the south side of the present-day Chinchihuapi Creek and the other from the MV-II site in the north bank terrace of the creek, correlate chronologically and sedimentologically. An OSL date reveals the MV-II occupation resting on the palimpsest surface of MV-7 stratum. Stratum MV-7 was deposited around 42,000 years ago and then exposed ~15,000 years ago when the creek formed (stratum MV-6 is the ancient creek bed). Around 15,000 years ago Chinchihuapi Creek downcut and washed away the upper and middle levels of stratum MV-7 in the north section (the ~40,000-14,000 year upper and middle layers of stratum MV-7 shown in section D of the profile of Unit 45(A) were not eroded). While the ~42,000 year surface was exposed ~15,000 years ago in the north section, the MV-II occupation took place shortly afterwards ~14,500 years ago and then sometime before 14,000 years ago both the exposed ~42,000 year surface of stratum MV-7 and the MV-II archaeological remains resting on it in the north section were buried by the subsequent deposition of strata MV-1 to MV-5. As indicated by the new evidence reported here, the uneroded surface and upper level of stratum MV-7 on the higher south side (Unit 45(A)) of the creek also were occupied between ~14,500 and 14,000 years ago; c-g. depositional and erosional Phases I-V schematically represent the sedimentological and archaeological sequence across site areas: c. Phase I: beginning before ~45,000 years ago the high terrace in site MV-I (Unit 45 (A)) was formed and comprised of intermittent shallow channels making up the braided stream system of the sandur plain. The channels roughly flowed from southeast to northwest; d. Phase II: after ~33,000 years ago the upper and middle levels of stratum MV-7, the high terrace levels as depicted in Unit 45 (A), were eroded in some places (curvy yellow line), then between 28,000 and 15,000 years ago new deposits of shallow braided stream channels were formed, including the airborne deposition of tephra lenses and the subsequent intermittent seasonal occupations by humans between 19,000 and 15,000 years ago. Sediment deposition during this phase was not continuous as indicated by non-depositional hiatuses; e. Phase III: ~15,000 years ago, deep lineal erosion occurred in some places and the Chinchihuapi Creek was formed (the date for the upper level of stratum MV-6, the creek bed, is shown in b,f and g), washing away the upper and middle levels of stratum MV-7 in the north section and exposing the surface of the ~42,223 year level of this stratum, upon which the MV-II occupation took place: f. Phase IV: a soil contemporary with the deposition of stratum MV-4 developed in the area of Unit 45(A) in the south section and strata MV-4 and MV-5 formed over and sealed the MV-II site in the north section; g. Phase V, strata MV-1 to MV-3 formed in both sections.
