# Supplementary material for: New Archaeological Evidence for an Early Human Presence at Monte Verde, Chile
Source: PLoS One. 2015 Nov 18;10(11):e0141923. doi: 10.1371/journal.pone.0141923 (PMC4651426; doi:10.1371/journal.pone.0141923)
Supplement: S3 Fig — a: arrows point to one of several thin, orange pumice lapilli lenses typical of those found throughout the MV-7 stratum containing the archaeological horizons; b: ventral side of a percussion flaked tool embedded in a 2 cm thick tephra lens located on an uneroded, elevated surface between narrow drainage channels in stratum MV-7, Unit 56 (see Fig 6b for a close-up of the dorsal side of the flake); c: typical micro-setting where thin orange lenses form during the summer months in shallow humid grassy areas at Monte Verde. (PDF) [file pone.0141923.s003.pdf]

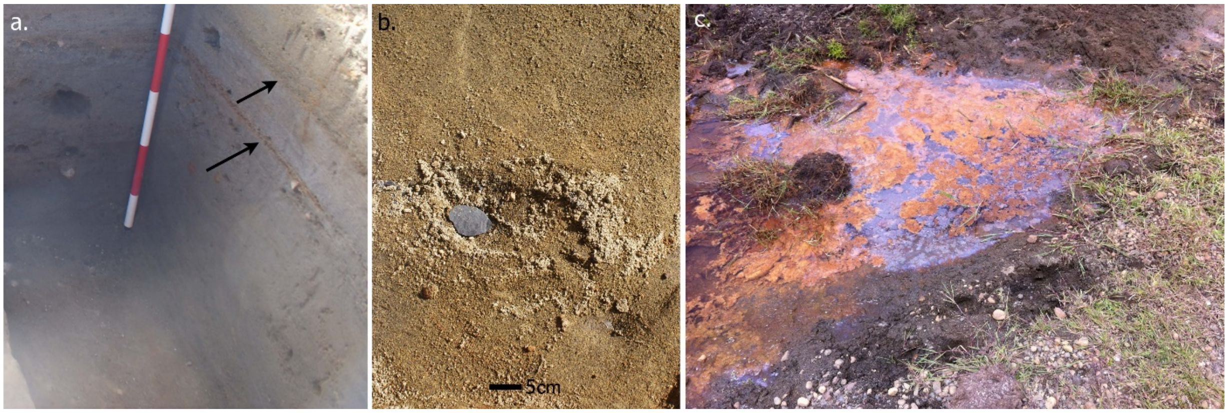

S3 Figure. Thin tephra lenses in the past and present: a: arrows point to one of several thin, orange pumice lapilli lenses typical of those found throughout the MV-7 stratum containing the archaeological horizons; b: ventral side of a percussion flaked tool embedded in a 2 cm thick tephra lens located on an uneroded, elevated surface between narrow drainage channels in stratum MV-7, Unit 56 (see Fig. 6b for a close-up of the dorsal side of the flake); c: typical micro-setting where thin orange lenses form during the summer months in shallow humid grassy areas at Monte Verde.
