# Supplementary material for: New Archaeological Evidence for an Early Human Presence at Monte Verde, Chile
Source: PLoS One. 2015 Nov 18;10(11):e0141923. doi: 10.1371/journal.pone.0141923 (PMC4651426; doi:10.1371/journal.pone.0141923)
Supplement: S4 Fig — a. Oval-shaped object of clay associated with a percussion struck lithic in Unit 55; b. Feature 9 in Unit 45(A) showing underlying orange tephra lens and overlying burned area with in situ patch of charcoal (bottom arrow) and percussion flake (top arrow). (PDF) [file pone.0141923.s004.pdf]

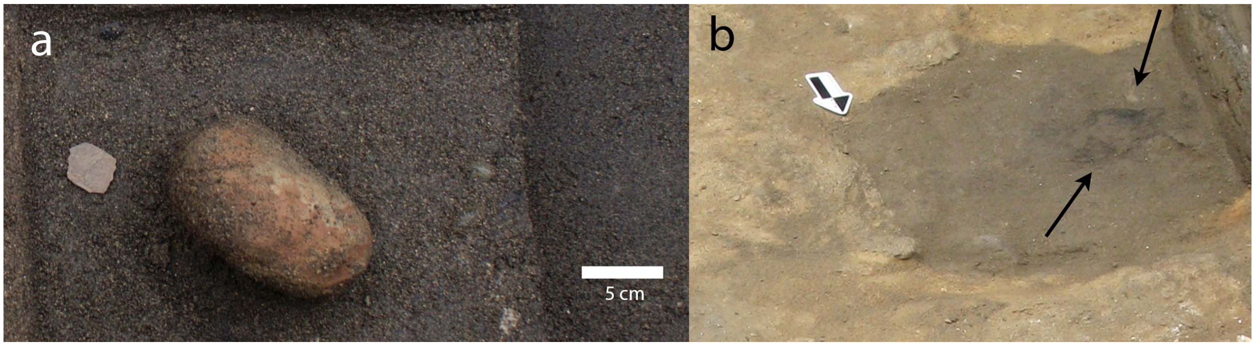

S4 Figure. a. Oval-shaped object of clay associated with a percussion struck lithic in Unit 55; b. Feature 9 in Unit 45(A) showing underlying orange tephra lens and overlying burned area with *in situ* patch of charcoal (bottom arrow) and percussion flake (top arrow).
