# Supplementary material for: New Archaeological Evidence for an Early Human Presence at Monte Verde, Chile
Source: PLoS One. 2015 Nov 18;10(11):e0141923. doi: 10.1371/journal.pone.0141923 (PMC4651426; doi:10.1371/journal.pone.0141923)
Supplement: S5 Fig — Fractured and partially burned epiphysis of a tibia probably of a deer or horse from Unit 56, MV-I dated at 13,940–13,560 cal BP (see Table 1). (PDF) [file pone.0141923.s005.pdf]

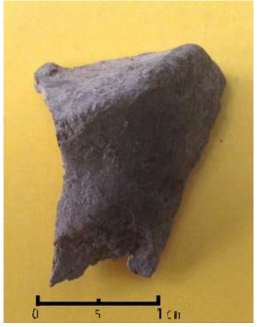

Figure S5. Fractured and partially burned epiphysis of a tibia probably of a deer or horse from Unit 56, MV-I dated at 13,940-13,560 cal BP (see Table 1).
