# Supplementary material for: New Archaeological Evidence for an Early Human Presence at Monte Verde, Chile
Source: PLoS One. 2015 Nov 18;10(11):e0141923. doi: 10.1371/journal.pone.0141923 (PMC4651426; doi:10.1371/journal.pone.0141923)
Supplement: S1 File — Text A. Description of Lithics. Text B. Chrono-Stratigraphy of the 2013 Cultural Materials. Text C. Mineral, Petrographic and SEM Analyses of Tephra Sediments. Text D. Micromorphological Analysis of Archaeological Sediments. Text E. Magnetic Analysis of Archaeological Sediments. Text F. Pollen, Phytolith, and Starch Grain Analyses of Features. Text G. Optimal Luminescence Dating. Text H. Lithic Micro-Usewear Study. (DOCX) [file pone.0141923.s006.docx]

**Supporting Information**

**Texts A-H**

**Text A. Description of the Lithic Artifacts Recovered from Monte Verde (MV-I)** **and Chinchihuapi Sites (CH-I and CH-II) in 2013**

Michael B. Collins

Thirty-nine stone specimens are reported from the 2013 excavations at the Monte Verde I and Chinchihuapi I and II sites [1-4]. Of these, thirty conform to eight descriptive categories established previously and seven are described in three categories unique to this study. A majority of the current sample is of stones accessible in nearby geologic contexts whereas a minority are either of lithologies that do not occur locally or have distinctive forms that do not occur locally. Overall, the present sample is closely similar to those previously described except that this sample is dominated by knapping debris (flakes and shatter), accounting for 19 of the 39 specimens. Concomitantly, two pieces (a core and a chopper) exhibit flake scars from knapping (see S1 Table for data on each object and S2 Table for temporal placement of lithic groups by specimen frequencies.).

Group 3, **Chopper** (N=1; S6a Fig., S7i Fig.). This has a large flake scar that creates an acute edge across one end of the stone. There are two segments of this edge that exhibit small flake removals that appear to be from use as a tool.

Group 4, **Core** (N=1: Fig. 6h; S7c Fig.). A stream-rounded pebble of an unknown exotic raw material exhibits at one end three natural, weathered facets, two are small and one is a large cleavage plane oriented diagonally across the stone. Three percussion flake scars originate from the acute edge of the large facet, which was used as a striking platform, and extend down the sides of the stone. There is also slight excavation damage on the large platform facet that is visually fresh compared to all of the other facets and scars.

Group 5f, **Flakes** (N=11; Fig. 6b,d,e,f,i,k; S7b,d,e,f,g,h Fig.). Seven complete and four proximal flake fragments, nine of a local basalt and two of an unsourced exotic limestone (preliminary analyses suggest the source area may be western Argentina possibly near Neuquen or the Central Valley of Chile; George Dix, personal communication, 2015), generally have strong bulbs and radial ridges, suggesting uniform use of percussion for detachment. One is a primary flake, five are secondary, and five have fully non-cortical exteriors. Four platforms are crushed, two are cortical, and five are faceted. From the cortical surfaces present, all seem to derive from stream-rounded pieces of raw material.

Group 5s, **Shatter** (N=8; Fig. 6j). These 8 pieces, all of local basalt, were found together in a small cluster, evidently representing the place where a piece of stream-rolled basalt was knapped. Seven pieces are decidedly angular. Small remnants of a shattered platform remain on one piece; three lack clear flake attributes. Two exhibit stream-smoothed cortical exteriors and two lack cortex. Such debris is commonly produced in experimental knapping of basalt. Four of these are too small for meaningful measurements. The tight clustering of these fragments (within a ~25 cm^2^ area) with uniform, relatively fresh, high-impact fracture scars is not natural in this setting.

Group 8, **Edge-battered Stone** (N=1). This piece is split from a rounded pebble of local andesite with an oval, plano-convex overall configuration with acute edges around both ends and one site formed by the intersection of the convex exterior of the stone and the planar fracture facet. The remaining edge is heavily flaked and crushed from percussion blows struck from the convex surface, creating a faceted bevel onto the flat surface. This flaked edge is dulled, but it is not clear whether any of the dulling is from use.

Group 12, **Single Faceted Stones without Macroscopic Evidence for Use** (N=4). This group consists of split stream-rounded pebbles, one, of rhyolite, is wedge-shaped in longitudinal section and was evidently intentionally split by a single percussion blow. Three are plano-convex, of exotic quartzite and andesite lithologies, and seemingly formed in nature by cleavage along a natural plane in the stone. Acute edges around the peripheries show no clear macroscopic evidence for use.

Group 14, **Multifaceted Stone without Macroscopic Evidence for Use** (N=1). An angular piece of exotic quartz, roughly pyramidal in overall shape, has multiple facets, all evidently natural, weathered to different degrees. Corners and edges are all rounded, also to varying degrees. This would seem to be an unused stream pebble, a probable manuport.

Group 22, **Subspherical Stone** (N=1; S4c Fig.). This is a well-rounded pebble of basalt with a smoothed but pitted surface that seems natural. The index of sphericity, at 0.856, is within the subspherical range. Such stones comprised nearly 18% of one sample of Chinchihuapi Creek gravel from non-cultural contexts but in the context of the site was a manuport that could have served as a sling stone without modification or possibly be ground to a higher degree of sphericity (matte-finished spheroids). Collins [4] has previously described the macro- and micro-characteristics of possible sling stones at the Monte Verde site.

Group 23, **Rounded**, **Faceted Stones** (N=2; Fig. 6c). Angular, faceted stones of exotic basalt and andesite, with all surfaces and edges exhibiting stream wear and polish, lacking any apparent cultural facets or traces of use wear, but, once again, in the site it is likely a manuport.

**Round, Smooth Stones** (N=2). These are small, well-rounded stream pebbles of local andesite lithology lacking any evidence for human modification, but they are manuports. They have indices of sphericity of 0.699 and 0.692, well below the 0.800–0.899 and 0.900–0.999 indices, respectively, and surficial microscopic features for the subspherical and spherical stones proposed previously by Collins [4].

**Discoid Round, Flattish Smooth Stone** (N=1; Fig. 6g). With a discoidal shape and very smooth surfaces, this piece has the typical attributes of a beach worn pebble and is therefore considered to be a manuport derived from the Pacific shoreline, minimally 50 km or so to the west. There are no cultural modifications discernable on the stone, which is of an exotic lithology.

**Serpentine Pebble** (N=1; Fig. 7). A small, stream-rolled pebble of serpentine is deeply patinated with multiple bifacial flake scars on one end. There is crushing along the rather dull margins produced by these flakes. The flake removals detached the patinated surface, but the scars are not fresh. It is unclear why such a soft stone was flaked. This material is exotic to the Monte Verde area and known to come from the coastal cordillera.

**Grinding Stones** (N-3). Three grinding for plant food preparation were retrieved from 2013 test pits in the MV-II site. These are made of local granites and basalts and contain various phytolith, pollen and/or starch grains of economically useful plants as reported in a later section on the micro-botany study.

**Projectile Point** (N=1; Fig. 8). A Paijan-like projectile point made of rhyolite was recovered from an upper level of stratum MV-7 in CH-I.

**Basalt Wedge (N=1; Fig. 7)**. This small polygonal piece of basalt is a flake, multifaceted on its obverse face (7 facets) and on its reverse face (2 facets). Its edges are predominantly acute and are relatively sharp; one edge, opposite the platform, shows rounding indicative of use. One obverse facet is weathered cortex, 4 are moderately weathered, and 2 are minimally weathered. The reverse face consists of a large, single facet with bulb and radial shatter lines and a small fracture plane near one edge. The piece is small—only about 5 cm across—and of low mass (30.0g.; S2 Table). It is inferred that the human modification of this object includes the entire reverse face and all but the cortical facet and a small concavity adjacent to that cortical surface on the obverse. It seems to be a flake detached with considerable force from an already faceted core or similar object. The two major facets on the obverse also have pronounced radial shatter lines emanating from the same platform as those on the reverse and appear to have been detached simultaneously (i.e. by the same blow). There is dull sheen, emanating from the dulled edge, on the ridges between facets on the obverse. The force required to simultaneously detach these three flakes and produce their high energy attributes exceeds what seems possible given the low mass of the piece unless it were securely supported at the thin, opposite edge. The inference offered here is that this flake may have been struck with a sharp blow with a hard hammerstone while being used as a wedge in a large piece of wood or bone. Thus the necessary conditions would have been met for producing this constellation of attributes.


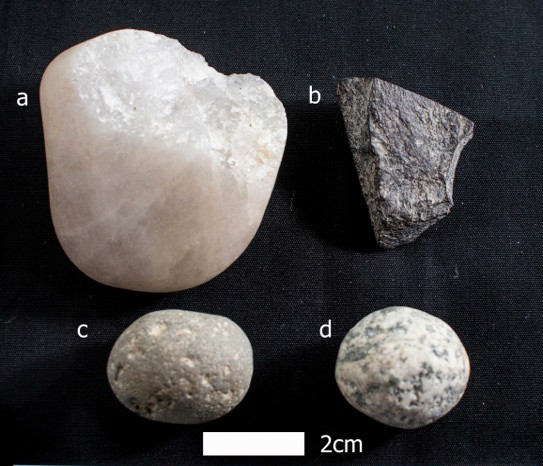


S6 Figure. Stone artifacts of the deeper, older level dated prior to ~25,000 cal BP in MV-(see S1 and S2 Tables): a. polished Group 3 pebble chopper of quartz probably derived from a beach of the Pacific Ocean (see S7i Fig.); one large flake has been removed culturally; b. Group 23 a spall of basalt with no cultural evidence; c and d. Group 22 possible sling stones with dull matte finishes.


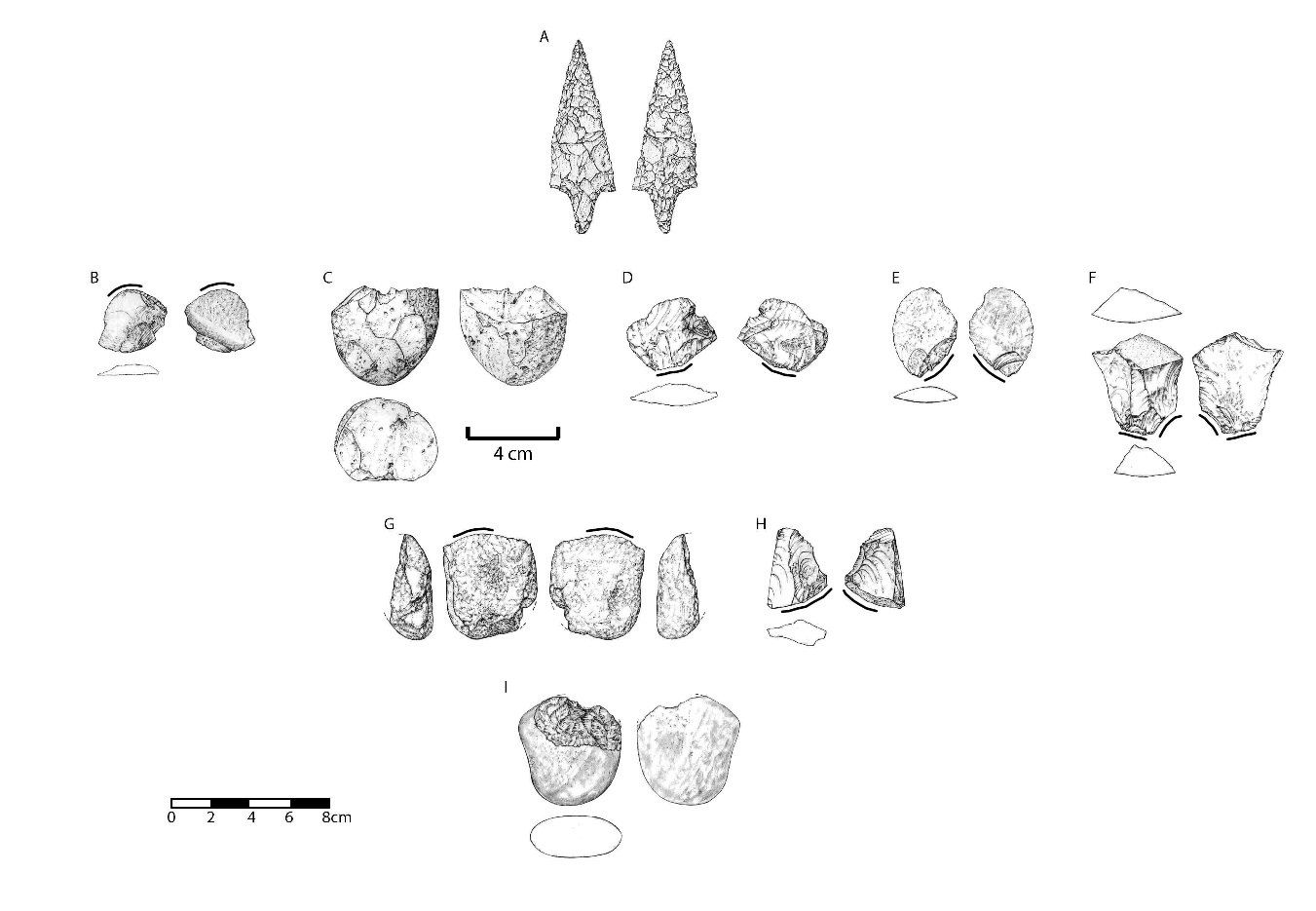


Figure S7. Selected drawings of stone tools from different chronological periods: a:

Paijan-like point dated around 10,600 cal BP; b-f: core and unifacial flakes dated between ~16,000 and 14,500 cal BP; g-h: unifacial flakes dated between ~18,000 and 16,000 cal BP; i: quartz chopper dated after 25,000 cal BP? Micro-usewear analysis was performed on selected edges (black lines) of various lithics. Note: Scale for specimen “c” is different from scale for others.

S1 Table. Descriptive data on 2013 lithics by Collin’s Group types: Measurements in ( ) are of broken pieces; Length of flakes taken parallel to axis of flaking; Width taken perpendicular to axis of flaking; *small angular shatter pieces not measured, weight reported as mean of the four largest pieces.

| **GROUP1** | **SPECIMEN (depth in m)#** | **^2** | **LITHOLOGY** | **LENGTH**  Mm | **WIDTH**  Mm | **THICKNESS**  Mm | **WEIGHT** g |
| --- | --- | --- | --- | --- | --- | --- | --- |
| 3^@^ | MV-1-55/1.75 | 18 | Quartz | 54.1 | 50.0 | 20.5 | 73.9 |
| 4 | MV-1-55/1.17 | 28 | Unknown | 44.0 | 52.2 | 46.0 | 132.8 |
| 5f^@^ | MV-1 82/1.43 | 3 | Basalt | (15.0) | 15.2 | 4.0 | 1.0 |
| 5f^@^ | MV-1-87/1.30 | 4 | Basalt | 20.3 | 18.8 | 3.2 | 1.1 |
| 5f^@^ | MV-1-55/1.47 | 5 | Basalt | (27.2) | 31.6 | 9.3 | 5.9 |
| 5f^@^ | MV-1-56/1.15 | 7 | Unsourced limestone | 36.0 | 44.0 | 8.3 | 11.8 |
| 5f^@^ | MV-1-56/1.45 | 9 | Unsourced limestone | 51.2 | 45.0 | 18.0 | 37.7 |
| 5f^@^ | CH-1/1.47 | 12 | Basalt | 30.0 | 36.8 | 4.0 | 4.7 |
| 5f^@^ | CH-1/1.47 | 17 | Basalt | 47.1 | 32.3 | 9.0 | 10.1 |
| 5f^@^ | MV-1-55/1.18 | 20 | Basalt | 16.0 | 23.6 | 8.1 | 1.8 |
| 5f^@^ | MV-1-55/1.18 | 21 | Basalt | 11.5 | 14.0 | 5.0 | 1.2 |
| 5f^@^ | MV-1-55/1.18 | 23 | Basalt | 15.0 | 10.0 | 6.7 | 1.6 |
| 5f^@^ | MV-1-55/1.18 | 27 | Basalt | 20.1 | 15.4 | 7.0 | 1.7 |
| 5s (N=4)^@^ | MV-1-55/1.15 | 19 | Basalt | * | * | * | 0.7 |
| 5s | MV-1-55/1.16 | 22 | Basalt | 23.0 | 19.5 | 8.4 | 2.5 |
| 5s | MV-1-55/1.18 | 24 | Basalt | 16.0 | 11.1 | 5.9 | 2.1 |
| 5s | MV-1-55/1.16 | 25 | Basalt | 19.0 | 11.4 | 4.0 | 2.2 |
| 5s | MV-1-55/1.16 | 26 | Basalt | 22.0 | 13.1 | 5.0 | 2.4 |
| 8^@^ | MV-1-57/1.32 | 11 | Andesite | 38.0 | 24.0 | 9.4 | 9.9 |
| 12^@^ | MV-1-55/1.14 | 6 | Rhyolite | 50.0 | 45.2 | 23.0 | 64.4 |
| 12^@^ | MV-1-57/1.42 | 15 | Quartzite | 39.0 | 31.2 | 12.7 | 73.3 |
| 12^@^ | MV-1-57/1.17 | 16 | Andesite | 48.2 | 39.0 | 24.6 | 33.8 |
| 12^@^ | MV-1-57/1.18 | 13 | Quartzite | 50.0 | 37.2 | 17.9 | 36.8 |
| 14 | MV-1-57/1.15 | 14 | Andesite | 41.0 | 38.0 | 35.0 | 17.9 |
| 22^@^ | MV-1-45/1.73 | 30 | Unknown | 28.0 | 23.0 | 20.5 | 17.6 |
| 23 | MV-1-57/1.36 | 2 | Andesite | 41.0 | 31.1 | 10.8 | 23.1 |
| 23 | MV-1-55/1.72 | 29 | Basalt | 34.4 | 31.0 | 10.4 | 12.2 |
| Round faceted | MV-1-17/1.72 | 1 | Rhyolite | 25.9 | 23.4 | 20.8 | 18.0 |
| Round smooth | MV-1-57/1.47 | 8 | Andesite | 28.1 | 23.0 | 20.0 | 19.2 |
| Discoid | MV-1-56/1.22 | 10 | Unknown | 50.0 | 43.4 | 13.3 | 44.5 |
| Flaked serpentine pebble | MV-1-17/1.49 | 31 | Serpentine | 48.4 | 30.1 | 20.2 | 15.9 |
| Wedge | MV-1-82/1.32 | 32 | Basalt | 51.0 | 50.0 | 23.0 | 30.3 |

# Depths vary slightly across the buried undulating levels of stratum MV-7.

^@^ Lithics subjected to micro-usewear.

Notes: ^1^groups from Collins 1997; ^^2^analytical serial number; groups “Round,” “Discoid,” “Flaked Serpentine Pebble,” and “Wedge,” refer to groups unique to this analysis. Measurements in ( ) are of broken pieces; Length of flakes taken parallel to axis of flaking, Width taken perpendicular to axis of flaking; *small angular shatter pieces not measured, weight reported as a mean of the four.

S2 Table. Temporal placement of lithic groups by specimen frequencies.

| GROUP | <25,000 cal BP? | 19,000 –  17,000 cal  BP | 16,000 –  15,000 cal BP | 15,000 –  14,500 cal  BP |
| --- | --- | --- | --- | --- |
| 3 | 1 |  |  |  |
| 4 |  |  |  | 1 |
| 5f |  | 5 | 1 | 5 |
| 5s |  |  |  | 8 |
| 8 |  |  | 1 |  |
| 12 |  | 1 |  | 3 |
| 14 |  |  |  | 1 |
| 22 | 1 |  |  |  |
| 23 | 1 |  | 1 |  |
| Round | 1 | 1 |  |  |
| Discoid |  | 1 |  |  |
| Serpentine |  | 1 |  |  |
| Wedge |  |  | 1 |  |
| Totals | 4 | 9 | 4 | 18 |

Note: Three grinding stones from MV-II and the projectile point from CH-I not included.

**Text B. Chrono-Stratigraphy of the 2013 Cultural Materials**

Tom D. Dillehay, Mario Pino and Andre Oliveira

Sawakuchi

Several issues require more discussion in regard to the chronology and stratigraphy of cultural materials within and across sites.

1) There is strong agreement between the 14C and OSL dates for cultural and non-cultural lenses in the upper to lower levels of stratum MV-7 in sites MV-I, CH-I and CH-II where the 2013 archaeological horizons were recovered and where prior work recorded possible pre-14,500 cal BP cultural materials in site MV-I. A later discussion of the OSL results reveals the ages for the dated strata and levels in Units 45(A) (see Text G), where the majority of the OSL samples were taken. As explained below, the depth and age of the 14C dated archaeological horizons do not always correlate precisely to the depth and age of the OSL assays at different levels in stratum MV-7 across sites. The OSL samples were taken at interval levels in a single, intact profile of Unit 45(A) that was minimally marked by shallow down-cutting from wash (i.e., small braided channels) or archaeological horizons.

Specifically, slight discrepancies between some 14C and OSL measures for similar levels in stratum MV-7 across sites are explained by the following conditions. First, the OSL dates were taken from a single column in Unit 45(A) where there was little apparent erosion from low-energy seasonal rainwater and snowmelt. In this regard, an attempt was made to date a relatively unwashed and non-cultural profile from top-to-bottom for comparison with the relative depths and ages of other excavated unit profiles, especially those with cultural horizons. Second, in some areas of the sandur plain there were 10 to 30 cm of linear sediment erosion that was produced by seasonal wash probably over several years within the low-energy braided stream system, which intermittently exposed slightly deeper and older surfaces to human use and archaeological deposition (see S2 Fig.). Third, once an old surface was exposed by wash, the thin oxidized, airborne tephra lenses were deposited occasionally across the braided system of the sandur plain (i.e., stratum MV-7) [5,6]. The lenses were preserved only on elevated spaces between the narrow drainage channels where people later camped and discarded cultural materials forming the archaeological horizons and where grass subsequently grew rapidly during the warm months, apparently helping to preserve the lenses and the overlying horizons. Some of the older OSL dated surfaces and the younger radiocarbon dated horizons could have similar vertical depths and lateral associations, but not be contemporaneous, with the result being the tephra lenses and newly discovered archaeological horizons resting on or embedded in older surfaces and the different OSL and ^14^C ages at similar depths in stratum MV-7 (see S2 Fig.).

2) The radiocarbon and OSL dates were derived from several different unit profiles and geologic sections across the multi-site study area. Seven spatially discrete features and their associated lithic and/or bone materials were dated by radiocarbon means (see Table 1). However, the inferred ages of five other features and their associated cultural materials were interpolated based on stratigraphic positions in undated unit columns. When combined, the direct dates and interpolated ages were used to reconstruct a relative interpretation of the local geological and archaeological history.

As noted above in discussing the stratigraphic correlations between the ^14^C and OSL dates, there are slight variations in the depths and ages of features and their corresponding sediment levels in excavation units across the site, which varies with paleo-topography and/or modern topography. Slight variations in the depth and thickness of strata in some areas are due to differential deposition and hiatus, meaning periods of no deposition, but not necessarily erosion, during the late Pleistocene period. Although horizontally separated across different site areas, the thickness, depth and sediment characteristics of each stratum (i.e., MV-1 to MV-4 and MV-7) in each excavation unit and across the sandur plain of the study area are similar (see Text E). Furthermore, the top and bottom levels of stratum MV-7 in all site areas range from ~38 to 45 cm to 1.35 to 1.50 m in depth, respectively, below the actual ground-surface of each excavated unit. (Excavated units in site MV-1 are located on the top of a flat knoll with less than 10 cm of vertical variation in the individual strata.)

Although a fixed datum was employed for all excavated areas during the 2013 season in order to connect the vertical depths and horizontal positions of all excavated strata and cultural materials, the precise depth of specific features and cultural artifacts presented in Tables 1 and 2 and in Figure 4 are based on centimeters and meters below the datum point at actual ground-surface in the northeast corner of each excavation unit. Thus, the radiocarbon assays have precise local validity with respect to the strata and cultural materials within each dated unit, and collectively are utilized across all site excavations to interpolate the chronology of the entire study area. Table 1 presents new and previous radiocarbon dates for sites MV-I and CH-I (including a previously unpublished date derived from a core in the Unit 56 area in 2007 [MV-I/VM1,T4]).

Lastly, we attempted to radiocarbon date specific features and interval strata levels (e.g., MV-4, upper to lower levels of MV-7) for each stratigraphic column in each excavation unit in each site but because so few features were recovered within and across sites and because no other datable organic material (e.g., peat balls, non-cultural carbon) was recovered, some units were not directly dated. However, given that both the new and prior radiocarbon and OSL dates (see Table 1 and Text G) reveal an intact chrono-stratigraphic sequence in nine different, relatively closely spaced excavated units within an area of approximately 30 by 30 m [2], we are confident that the interpolated ages for undated strata and features are correct. Regardless of slight differences in the depth of the archaeological horizons in the middle and lower levels of stratum MV-7, all cultural materials located below the surface or upper levels of this stratum date before 15,000 years ago as indicated by both radiocarbon and OSL dating (see Table 1 and Text G).

3) The bone samples radiocarbon dated by Beta Analytic Inc. were not subjected to ultra filtration. However, the PRI bone date (see Table 1) was treated by ultra filtration. In terms of treatment, the Beta Analytic bones were first tested for friability ("softness"). Very soft bone material is an indication of the potential absence of the collagen fraction (basal bone protein acting as a "reinforcing agent" within the crystalline apatite structure). The bones were washed in de-ionized water and the surface scraped free of the outer most layers. Dilute, cold HCl acid was repeatedly applied and replenished until the mineral fraction (bone apatite) was eliminated.  Since the bones were solid pieces, the outermost collagen was also scraped and discarded as an additional measure of pretreatment during the initial stages of demineralization. The collagen was dissected in solution and inspected for rootlets and other organic debris (readily visible), which were not observed.  Cold 1% sodium hydroxide (50/50 wt% NaOH) was then applied to remove secondary organic acids. Visual observation of the collagen in solution confirmed “good collagen status”.  After drying through vacuum desiccation, the d13C was measured as the final proxy prior to the dating (Beta Analytic Inc., personal communication, 2015).

Regardless of the treatment methods, both the bone and charcoal dates concur stratigraphically (i.e., depth and context) and chronologically, implying that the type of bone treatment probably had little, if any, effect on the processed ages presented in this study (see Fig. 5 and Table 1). Although only one ultra filtration-treated bone date was obtained, its age chronologically and stratigraphically correlated with other ^14^C dates.

4) As for the %C and C/N ratios, Beta Analytic did not measure either of those variables because they were not considered to be critical to the results. Both are highly distorted from original content during the physical pretreatments and the collagen extraction plus alkali.  Although they are potential indicators of mass preservation of collagen” within a bone, they are not necessarily indicative of the “quality of collagen within the bone (quality defined as being primary carbon, contamination free and providing an accurate age (Beta Analytic Inc., personal communication, 2015).

**Text C. Mineral, Petrographic, and SEM Ray Florescence Analyses of Tephra Samples from Stratum MV-7**

Mauricio Mella

The Monte Verde site is located in an area near three major volcanoes, Osorno, Calbuco and Yate. The Calbuco volcano is closest to the site, located 52 km to the southwest (S8 Fig.), with common deposits of ash fall from medium and large eruption [7-9]. In the present study, ash layers measuring 0.5 cm in thickness (no. LMMR-085) were sampled from the MV-7 stratum in excavation Unit 82. The mineralogy and texture of the samples were also examined.

The Calbuco volcano (2,003 masl) is located respectively 30 km and 32 km northeast of the city of Puerto Montt and east of the city of Puerto Varas in the Province of Llanquihue, Lake Region, south-central Chile. It is a strato-volcano located in the southern end of the central segment of the Southern Volcanic Zone of the Andes (ZVSC. 37-42 ° S39) and characterized by andesitic, lava (i.e., orthopyroxene-clinopyroxene-plagioclase), and, to a lesser extent, olivine deposits [7-10]..

**Methods and Results**

A more finely resolved understanding of local stratigraphic sequences is provided by samples of airborne tephra (volcanic ash) deposits (thickness of 0.5 cm) from stratum MV-7 at MV-I, which was studied by geologists of the National Service of Geology and Mining (SERNAGEOMIN) of Chile. Binocular microscope, electron microscope images and semi-quantitative analyses were used to determine the major elements and clastic mineral constituents. The morphology and chemistry of the constituents of the LMMR-085 ash also were examined. These studies were carried out in the Electron Microscopy Laboratory at the National Service of Geology and Mining (SERNAGEOMIN) of Chile. A ZEISS model EVO MA-10 Electron Microscope (SEM) with an EDS Oxford X-ACT detector for semi-quantitative analysis of major elements was used to obtain detailed images of secondary electrons (SE) and backscattered electrons (BSE). Using X-ray diffraction also did identification of mineral species of sample 085 LMMR. The sample was manually separated and sprayed under 200 mesh in an agate mortar. Other equipment used for the analysis was a Panalytical model X Pert PRO diffractometer. The interpretation program used was the High Score Plusse. Tephra grain size and composition (andesite) were consistent with a volcanic source at Calbuco volcano, located 35 km to the southeast of Monte Verde. Geochemical correlations (determined by sample means that overlap at 1σ for all analyzed element oxides for a pair of samples) suggest that the MV-7 stratum preserves at least five different eruptive events. Our tephro-stratigraphic results permitted us to correlate outcrops that could not be related in the field because of discontinuous exposure at Calbuco (S8 Fig.). The same tephra deposit is found at Calbuco, indicating that both localities had sample sediments (and artifacts and features) of a similar temporal range.

Petrographic images were obtained by using SEM secondary electron (SE) and backscattered electron (BSE). A Green Monter petrographic study showed very angular clasts (S9 a-e Fig.), indicating a low null transport saltation and traction. This study

identified orthopyroxene, plagioclase, ilmenite and traces of intensely altered pumice (S9 a-e Fig., S3 Table). The data provided by the X-ray fluorescence showed compositions of quartz, sodium anorthite, potassium feldspar, amphibole and clinopyroxene. The semi-quantitative chemical study obtained by SEM also showed orthopyroxene, ilmenite-magnetite and plagioclase composition comparable in part with minerals from the Calbuco volcano [7-10].

S3 Table. Semi-quantitative analysis of glass, orthopyroxenos and plagioclasts of the LMMR---­‐-­‐-­‐085 sample.


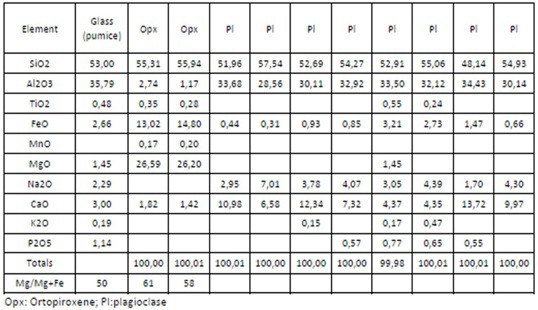


*X-Ray Flouresence Analysis, Laboratorio Químico del Servicio Nacional de*

*Geología y Minería, Informe de Rayos X N° 24/14*

The sample was removed manually and pulverized under 200 mesh in mortar agate. The equipment used for the analysis is a Panalytical diffractometer model X Pert PRO. The interpretation program is the High Score Plus. The qualitative mineralogical composition is as follows:

LMMR – 085: Quartz, anorthite sodium, potassium feldspar, sodium edenita, augite. The corresponding diffractogram is presented below.

Anchor Scan Parameters

Dataset Name: LMMR085

File name: C:\X'Pert Data\LMMR085.xrdml

Measurement Date / Time: 14---­‐-­‐-­‐04---­‐-­‐-­‐2014 11:52:04

Operator: Laboratory

Raw Data Origin: XRD measurement (*.XRDML) Scan Axis: Gonio

Start Position [°2Th.]: 5,0100

End Position [°2Th.]: 64,9900

Step Size [°2Th.]: 0,0200

Scan Step Time [s]: 0,5000

Scan Type: Continuous

Offset [°2Th.]: 0,0000

Divergence Slit Type: Fixed

Divergence Slit Size [°]: 0,4785

Specimen Length [mm]: 10,00

Receiving Slit Size [mm]: 0,1000

Measurement Temperature [°C]: 25,00

Anode Material: Cu

K---­‐-­‐-­‐Alpha1 [Å]: 1,54060

K---­‐-­‐-­‐Alpha2 [Å]: 1,54443

K---­‐-­‐-­‐Beta [Å]: 1,39225

K---­‐-­‐-­‐A2 / K---­‐-­‐-­‐A1 Ratio: 0,50000

Generator Settings: 30 mA, 40 kV Diffractometer Type:

0000000000030722

Diffractometer Number: 0

Goniometer Radius [mm]: 240,00

Dist. Focus---­‐-­‐-­‐Diverg. Slit [mm]: 91,00

Incident Beam Monochromator: No

Spinning: No


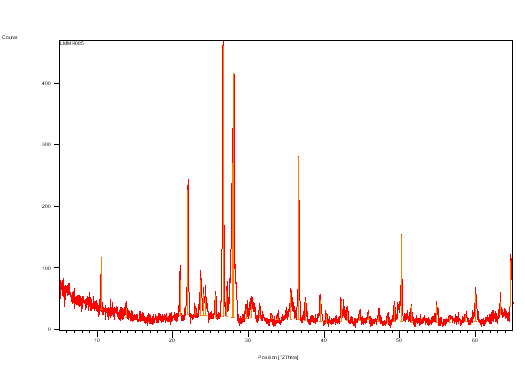


*Pattern List*

Visible Ref.Code Score Compound Name Displ.[°2Th] Scale Fac. Chem. Formula

| * | 01-085-0798 | 38 | Quartz | 0,000 | 0,960 | Si O2 |
| --- | --- | --- | --- | --- | --- | --- |
| * | 01-076-0829 | 17 | Potass.,sodium t.. | 0,000 | 0,282 | K0.94 Na0.06 Al0.9,.. |
| * | 00-023-0664 | 10 | Edenite, sodian, syn | 0,000 | 0,122 | (Ca, Na) 3Mg5 |
| * | 00-024-0203 | 7 | Augite | 0,000 | 0,094 | Ca (Mg, Fe) Si2,.. |
| * | 00-020-0528 | 35 | Anorthite, sodian,.. | 0,000 | 0,520 | (Ca, Na) (Al,... |

**Discussion**

The lavas and ash deposit falls of the Calbuco volcano are mostly characterized by clinopyroxene, orthopyroxene, plagioclase, magnetite and amphibole [9]. As stated by Lopez-Escobar et al. [9]: "Plagioclase, clinoyroxene, orthopyroxene and minor amphibole are the main phenocryst phases in andesite lavas from volcano Calbuco. Most plagioclase is abundant with phenocristals ranging from AN60 to AN90; clinopyroxene and othopyroxene have relative homogeneous Mg-numbers (MgO / MgO + FeO) from

0.50 to 0.59. Amphibole phenocristal is not abundant, but edenitic occurs, and

tschermakitic Mg-hornblendes are associated with phenocristals and xenocristals lavas." Taking this into consideration, the data suggest that the likely source of ash fall at Monte Verde is from the nearby Calbuco volcano. This interpretation is based on the following characteristics:

Very sharp crystals indicative of low to no transport; the presence of minerals such as orthopyroxene, amphibole, clinopyroxene and plagioclase that are typical of the Calbuco deposits (S9 a-b Fig.); the presence of euhedral orthopyroxene crystals with Mg/Mg + Fe (0.57-0.61), which again are most similar to the phenocrysts of the Calbuco volcano lavas [7], and the shard and pumice textures in altered clasts (S9 Fig.).

The geochemistry of glasses also is similar to those reported in the Calbuco volcano rocks [7]. One feature that stands out in the samples is the low proportion of juvenile clasts (pumice). Considering the various sedimentary structures of the sands and fine gravels overlying and underlying the ash falls in the low energy, subaquatic depositional environment of the sandur plain of the Monte Verde area, it appears that the lighter pumice in the lower troughs of the braided drainage system was transported downstream, leaving the crystals in situ. (This is the same interpretation of the study of the sedimentary composition of the stratum MV-7). This implies that human occupation occurred after the lighter pumice in the channel troughs was washed away. Finally, it should be noted that the water and oxidation states of Fe were not measured by SEM analysis, therefore it is not possible to make precise comparisons of the quantitative chemical data.


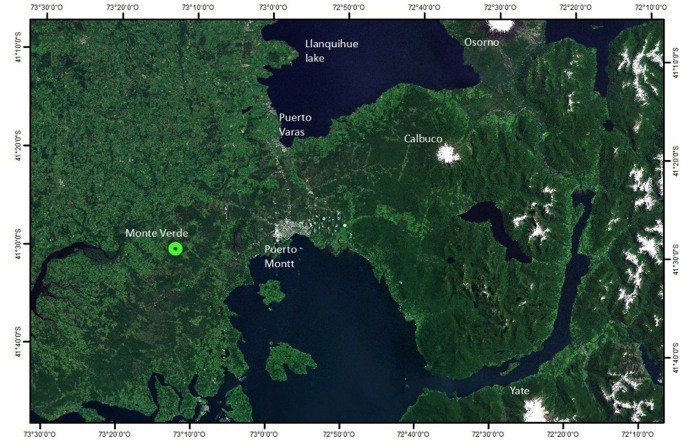


Figure S8. Monte Verde site location and its relation to the Osorno, Calbuco, and Yate volcanoes.


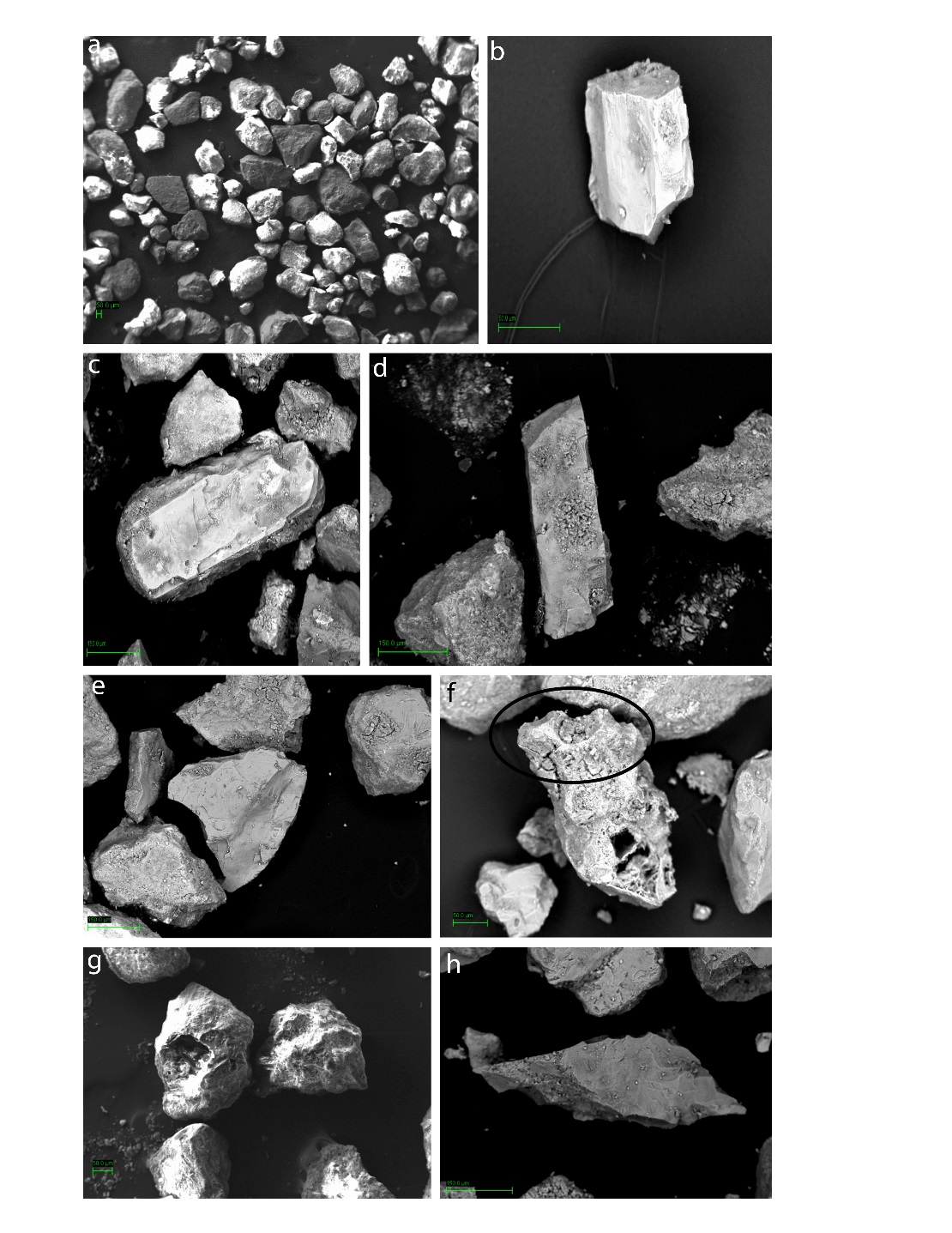


S9 Figure. LMMR Overview of the 085-sample with electrons: a: general view of the LMMR–085 sample showing electrons; b-c: hedenita euhedral orthopyroxene crystals. Mineral characteristic of andesitic magnas in the region of the Calbuco volcano. The sharp edges show low transport; d-e: euhedral and angular crystals of plagioclase labradorite showing low transport; f-g: altered pumice: usually its porous texture is obliterated by side fills. The characteristic morphology of an oval chip "sherd" is detailed; h: very angular quartz clasts.

**Text D. Micro-morphological Analyses of Sediments**

Ximena S. Villagran

For the purpose of assessing further the intactness and sediment characteristics of the archaeological deposits, a total of four undisturbed blocks for micro-morphological analysis were collected from the stratigraphy of the MV-I and MV-II sites. Three samples were collected from location Unit 45(A) in MV-I (S10a Fig.) and one sample from the Chinchihuapi Creek bank profile of site MV-II (S10b Fig.).

The undisturbed blocks were oven-dried for one week at 50º C. After completely dried, all blocks were impregnated with a mixture of polyester resin (Viscovoss N5 S, 700 ml), styrene (300 ml) and hardener (MKEP, 5-7 ml). Samples were put under a vacuum (200 mbar) for one hour and left to dry at room temperature for two weeks. After complete hardening of the resin, the samples were put in the oven at 50º C for 48 hours. The impregnated blocks were cut into 1 cm wide slabs and polished on one surface with an MPS-RS Vacuum polisher. The polished surface was glued to a 6 x 9 cm glass slide with a mixture of Araldite (50/50, 10 ml) and hardener (3,5 ml). After drying, the slabs were machine polished to 30 µm thin sections for observation under optical microscope in plane polarized light (PPL), cross-polarized light (XPL) and green fluorescent light. The analysis was done following the guidelines of Stoops [11]. Samples were prepared and analyzed at the Institute for Archaeological Sciences, Universität Tübingen, Germany.

**Results**

*Profile MV-I, Unit 45(A)*

Three samples were collected from this profile: (1) A, at the contact between strata MV-1 and MV-2; (2) B, at the contact between strata MV-2 and MV-3; and (3) and C, located in the middle of stratum MV-7 (S10a Fig.).

Sample A: (1) Microfacies (mF) of reddish soil (mF1, corresponding to stratigraphic stratum MV-1); (2) overlying yellowish clay microfacies (mF2, corresponding to stratigraphic stratum MV-2).

mF1: Red oxisol fragment with frequent clay and iron coatings and impregnations (S11a,b Fig.). The sharp contact with underlying mF2 and the presence of oxisol aggregates in mF1 suggest redeposited material and later reworking from the topsoil to the underlying sediment, possibly by biological action given the presence of mF2 material inside channel voids.

mF2: Yellowish aggregated and compact clay (S11a-c Fig.). Coarse fraction (frequency of 5%) made of quartz grains, feldspars, rock fragments and volcanic glass with one isolated charcoal fragment. Fissures in the micromass possibly caused during drying of the sample (S11d Fig.). Frequent iron nodules, both formed in situ and as inherited pedo-features. There also are common iron infillings, coatings and hypo-coatings (S11d Fig.). The sample gives the general appearance of clayey fluvial deposit.

Sample B: Yellowish aggregated clay microfacies (mF2, corresponding to stratum MV-2) over sandy clay (mF3, corresponding to stratum MV-3).

mF2: Same yellow clay seen in mF2 from sample A with aggregated and compact microstructure­­. Coarse fraction (frequency of 10%) made of quartz, feldspars and rock fragments. Frequent iron coatings, and compound coatings made of two or more generations of superposed coatings (S11e Fig.). The rare clay coatings are capped by iron coatings. At the contact with underlying mF3 there is a pebble of volcanic rock with signs of *in situ* weathering and visible formation of allophane. This allophane is light brown and limpid in PPL with low birefringence in XPL.

mF3: Sandy clay made of volcanic minerals and rock fragments cemented with allophane. Coarse fraction (frequency of 70%) made of quartz, feldspars, basalt, volcanic glass and other volcanic minerals and rock fragments. The allophane is light brown/beige almost transparent and limpid in PPL with undifferentiated b-fabric (XPL) (S11f Fig.). Clay and iron oxides are also present in the fine fraction. The allophane is not transported but formed in situ after weathering of volcanic rocks by groundwater.

Sample C: Sandy microfacies with allophane coatings (mF7), corresponding to stratum MV-7 (S10a Fig.). This sample had a groundmass similar to mF3 from sample B but without clay and iron oxides in the fine fraction. Coarse fraction (frequency of 70%) made of quartz, feldspars and volcanic minerals and rocks (S11g Fig.), coated and cemented by light beige, almost transparent (PPL) allophane with undifferentiated b-fabric (S11h Fig.). It gives the appearance of fluvial gravel.

*Profile from site MV-II, North Side of the Chinchihuapi Creek*

One sample was collected from this location: Sample D. The block was taken from the top of stratum MV-7 in an attempt to define the contact between this unit and the overlying stratum MV-6 (S10b Fig.).

Sample D: Gravel layer with iron coatings (mF6) corresponding to stratum MV-6, over a sandy layer of volcanic rocks (mF7) corresponding to stratum MV-7 (S11i-j Fig.).

mF6: Sandy gravel microfacies with poorly selected coarse fraction (frequency of 70%) made of quartz grains, feldspars and rock fragments. Iron coatings are around coarse fraction components (S11i Fig.).

mF7: Sandy microfacies with coarse fraction (frequency of ~90%) made of quartz grains, feldspars and volcanic minerals and rock fragments (S11g,h,j Fig.). It has no allophane coatings.

**Conclusions**

All samples come from natural deposits with no visible anthropogenic input or alteration (only one charcoal fragment was present in sample A). Bioturbation is visible in stratum MV-2 (mF2), mixing material from the topsoil (stratum MV-1) to the yellowish clay layer. In the underlying strata MV-3 to MV-7 bioturbation is almost nonexistent, especially in strata MV-6 and MV-7, where the archaeological levels were found.

Strong pedogenesis is seen in the yellowish stratum MV-2. Evidence of translocation and mixing (as described by Mücher [12]) characterizes stratum MV-2, such as: the aggregated and stacked appearance of the matrix; the frequent anorthic iron nodules (inherited pedofeatures); the inclusions of reddish soil rich in iron (oxisol, mainly on the top of the slide); and fragments of a Bt horizon (soil fragments with clay coatings).

Stratum MV-2 shows several generations of clay illuviation and iron formation, either caused by groundwater movement or by the natural accumulation of water favored by the geometry of the deposit and its fine grain-size. There is an erosive sharp contact between the yellowish clay (stratum MV-2) and the underlying volcanic material (strata MV-3 and MV-7), indicating two different depositional events. The volcanic material (strata MV-4 and MV-7) appears as alluvial sands with gravel.

The volcanic strata MV-3 and MV-7 show pedogenesis in the form of allophane formation. Allophane is a non-crystalline or poorly crystalline Si-Al component produced during initial stages of weathering of volcanic material under humid climates [13-15]. In strata MV-3 and MV-7, allophane appears cementing the volcanic rock fragments and mineral grains, whose chitonic microstructure is consistent with initial stages of soil formation [16].

Stratum MV-7 is visible in the profiles of MV-I-45(A) and of the MV-II site (north creek bank) with the only difference that allophane appears in the sample from the profile in MV-I-45(A). This could be due to the fact that stratum MV-7 comprises a sequence of several episodes of deposition of volcanic material, which can happen during different eruption episodes or even within a single eruption cycle. Allophane formation in the volcanic material sampled in MV-I-45(A) denotes more pedogenesis. Other possibilities to explain the differential pedogenesis are more proximity to the surface of stratum MV-7 in profile MV-I-45(A), which favored pedogenesis in this location, and frequent water saturation of stratum MV-7 in site MV-II due to its proximity to the Chinchihuapi Creek water table, which prevented pedogenesis in this location.

**
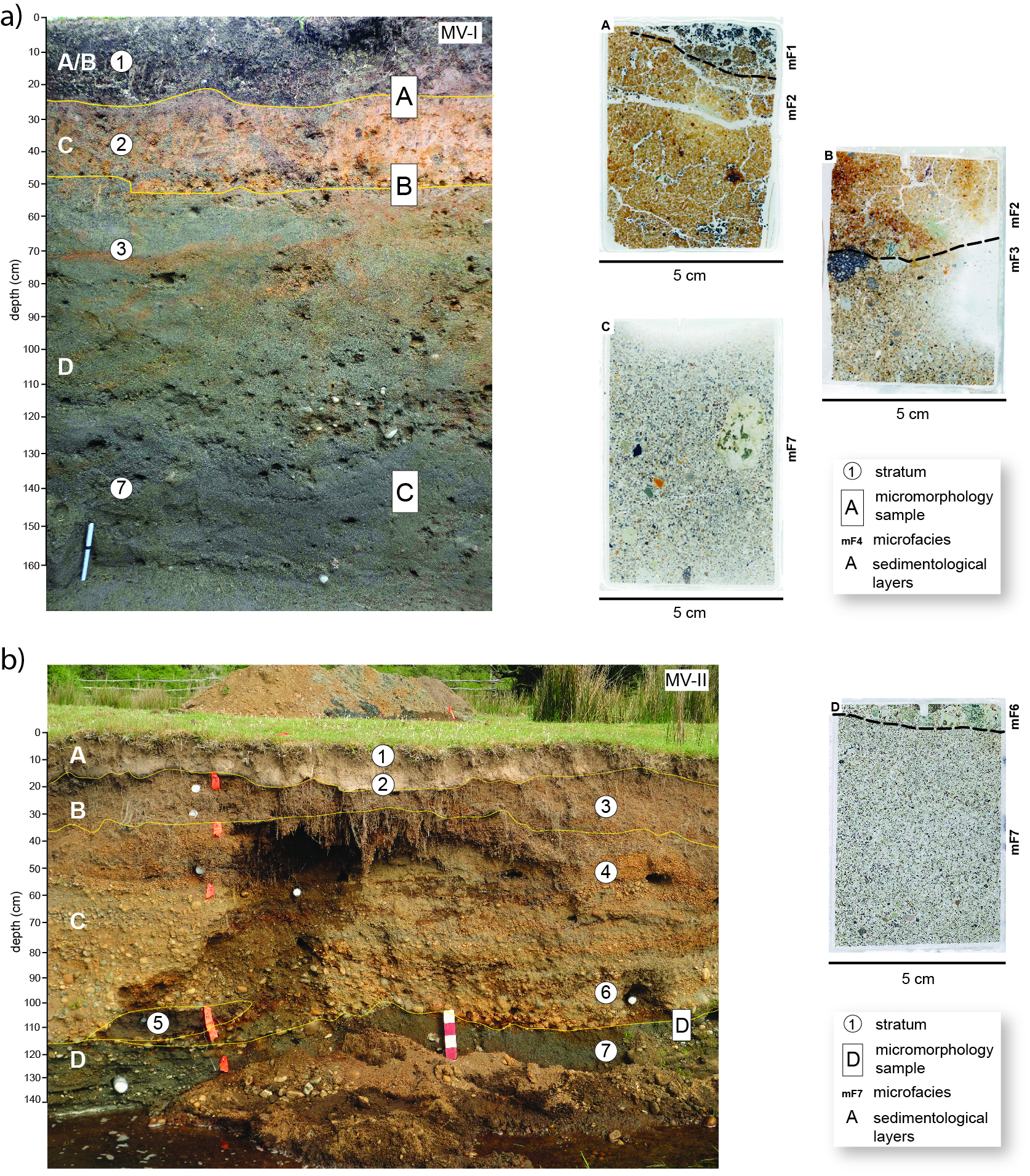
**

Figure S10a-b. Profiles of MV-I (a), Unit 45(A), and MV-II sites (b), with location of micromorphological samples and scanned thin sections with microfacies (mF) identification.


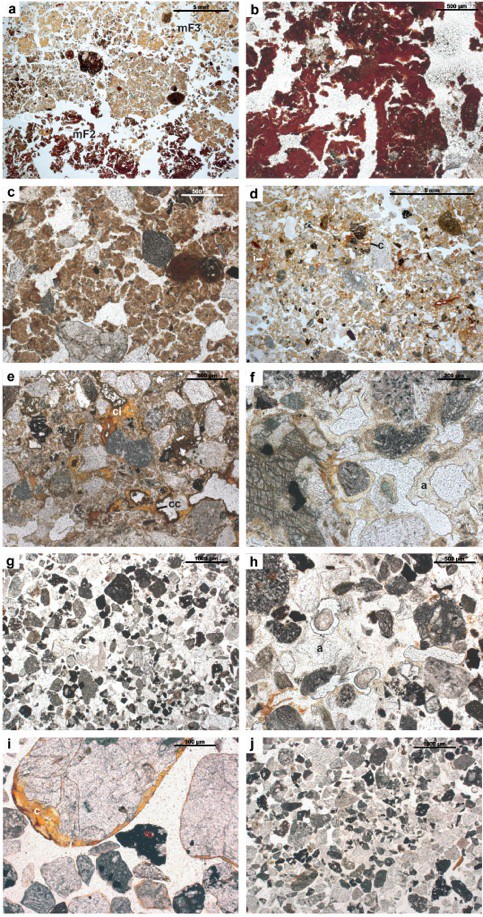


S11 Figure. Photomicrographs from samples A, B and C from profile MV-I-45(A) and sample D from profile MV-II (PPL): a) contact between mF1 (reddish soil) and mF2 (yellowish clay) in sample A; b) groundmass of reddish soil aggregate with iron impregnations in mF1, sample A; c) yellowish clay with fissures in mF2, sample A; d) groundmass of mF2, sample B, with iron coatings (c) and iron infillings (i); e) mF2 from sample B with compound iron infillings (ci) and compound coatings (cc); f) allophane coatings (a) in mF3, sample B; g) groundmass of mF7, sample C, made of volcanic rocks and minerals; h) allophane (a) in mF7, sample C; i) iron coatings (c) in gravel mF6 overlying mF7 in sample D; j) groundmass of mF7, sample D, made of volcanic rocks and minerals.

**Text E. Magnetic Analysis of the Archaeological Sediments**

Gelvam A. Hartmann

Sediment samples were collected in two different stratigraphic profiles from the MV-I and MV-II sites. In MV-I, Unit 45(A) profile, samples were collected at each 5 cm level of depth, comprising 30 samples to a maximum depth of 1.8 m. An additional 7 samples were collected from a MV-II profile on the north side of Chinchihuapi Creek; they correspond to the same samples used for 14C and OSL dating purposes. For the magnetic mineralogy studies, each sediment sample was prepared in an 8 cm3 cubic plastic box.

**Methods**

All magnetic measurements were performed at the Paleomagnetic Laboratory of the Institute of Astronomy, Geophysics and Atmospheric Sciences, University of São Paulo (IAG/USP). The low-field magnetic susceptibility of each discrete sample was measured using a Kappabridge MFK1-FA (AGICO Ltd) system with an excitation frequency

of 976 Hz and an applied field of 200 A/m. Natural remnant magnetization (NRM) and

isothermal remnant magnetization (IRM) measurements were performed using a JR-6A Spinner Magnetometer (AGICO Ltd). The IRM acquisition curves were obtained using a Pulse Magnetizer MPMP 10 (Magnetic Measurements) with maximum DC fields of

~2600 mT and correspond to the saturation of IRM (SIRM). The S-Ratio of each

sample was determined using a ratio of IRM magnetizations, i.e., the ratio between magnetizations acquired in the maximum +2600 mT DC field (SIRM) and a-300 mT DC backfield (IRM-300 mT). The S-Ratio was computed as S = |IRM-300 mT/ SIRM |. S- Ratio indicates the relative contribution of high-coercivity minerals (e.g. hematite, goethite) to low-coercivity minerals (e.g., magnetite [17-18].

**Results**

MV-I Site**:** Results of mass-normalized magnetic susceptibility show a rapid increase from the top down to the 55 cm depth (S12a Fig.). Between 55 and 160 cm depth, the magnetic susceptibility varies between ~6x10-6 and ~2x10-5 m3/kg, which indicates undisturbed soils. The archaeological horizons of the 1977 to 1985 and 2013 field seasons were recovered from depths ranging between 95 and 170 cm. SIRM magnetic results vary between ~2.5 A/m and ~239 A/m, with a gradual increase from 0 to 55 cm in depth. For the 55-160 cm depth, the SIRM results vary less than the values observed for the upper levels. S-Ratio values also show a gradual variation for the interval of 0-55 cm depth; in contrast, the values for 55-160 cm depth vary less than that observed for the 0-55 cm depth. The 0-55 cm levels correspond primarily to strata MV-1 to MV-4, which are culturally sterile (MV-5 and MV-6 correspond to only the Chinchihuapi Creek basin.)

All three magnetic parameters present a similar pattern for 0-55 cm depth. The magnetic mineralogy observed at depths of 0-55 cm suggests the presence of hematite and/or goethite and a posterior increase for magnetite. Magnetic parameters for the 55-160 cm depth indicate similar patterns. Magnetic analyses indicate the presence of magnetite and no rapid changes could be associated with discrete sediment deposition. In addition, the absence of strong variations in magnetic parameters indicates no vertical mixing of sediments between 55 and 160 cm, which correspond to the 95-170 cm depths of the excavated MV-I archaeological horizons.

MV-II site: Results of MV-II are shown in S12b Figure. Magnetic susceptibility varies between ~1x10-6 (~50 cm) and ~6x10-6 m3 /kg (~140 cm). Rapid variations in magnetic susceptibility are observed between 20-50 cm depth. From 60-140 cm depth, magnetic susceptibility present similar values with the maximum value at 140 cm depth. SIRM vary from ~12 (~50 cm) to 145 A/m (~140 cm). A strong difference is observed for the SIRM at 140 cm depth when compared with data for the upper levels. This result could indicate a different magnetic mineralogy at this depth. S-Ratio values suggest an increase in magnetite content with depth, with a maximum value observed at 140 cm depth.

As observed for the MV-I profile, the three magnetic parameters presented in MV-II reveal similar patterns in depth. Strong variations are observed between 20-50 cm in depth, indicating the presence of hematite and/or goethite with low magnetite contribution. Between 60 and 110 cm in depth, the magnetite contributions are similar, suggesting undisturbed soils. The MV-II archaeological materials dated to

~14,500 cal BP are located between the 98 and 102 cm levels, which from a taphonomic archaeological perspective also indicate intact deposits, with no bioturbation or other disturbance. In contrast, magnetic data at 140 cm depth are different in comparison with the upper levels, which indicate a higher magnetite contribution. This strong variation observed in all magnetic parameters suggests a different pedological composition at this depth. In addition, these results are similar to those obtained for depths of 55-160 cm observed in the MV-I profile, which indicate that the same stratigraphic levels can be correlated between profiles of the MV-I and MV-II sites.

S12 Figure: a. Magnetic data from profile MV-I, Unit 45(A); b: Magnetic data from the

bank profile of Chinchihuapi Creek in site MV-II.

**Text F. Pollen, Phytolith and Starch Grain Analyses of Samples from MV-I and MV-II**

Linda Scott Cummings

Pollen, starch grain and phytolith sediment samples were collected from two burned features recovered from the MV-I site. In addition, the sediments recovered from three grinding stones recently excavated in the MV-II site were examined for these materials.

**Methods**

A chemical extraction technique based on flotation is the standard preparation technique used at the PaleoResearch Institute for recovering pollen grains from sediments. This particular process was developed for extracting pollen from soils where the preservation has been less than ideal and the pollen density is lower than in peat. It is important to recognize that it is not the repetition of specific and individual steps in the laboratory but rather mastery of the concepts of extraction and how the desired result is best achieved, given different sediment matrices, that results in successful recovery of pollen for analysis. Our normal pollen and starch extraction methods were altered slightly for these samples. Hydrochloric acid (10%) was used to remove calcium carbonates present in the sediments. No reaction was observed with the HCl, indicating the sediments were not alkaline. The samples were screened through 250-micron mesh, then rinsed until neutral by adding water, allowing the samples to stand for 2 hours, and pouring off the supernatant. Due to the relatively small quantities that were available for each sample (between 10 and 17.5 cc for samples 1-4), we used centrifugation to remove clays prior to heavy liquid separation. The samples then were freeze-dried. Sodium polytungstate (SPT), with a density of 1.8 g/ml, was used for the flotation process. The samples were mixed with SPT and centrifuged at 1,500 rpm for 10 minutes to separate organic from inorganic remains. The supernatant containing pollen and organic remains was decanted. Sodium polytungstate again was added to the inorganic fraction to repeat the separation process. The supernatant was decanted into the same tube as the supernatant from the first separation. This supernatant then was centrifuged at 1,500 rpm for 10 minutes to allow any remaining silica to be separated from the organics. Following this, the supernatant was decanted into a 50-ml conical tube and diluted with reverse osmosis de-ionized (RODI) water. These samples were centrifuged at 3,000 rpm to concentrate the organic fraction in the bottom of the tube. This pollen-rich organic fraction was rinsed, then all samples received a short (20–30 minute) treatment in hot hydrofluoric acid to remove remaining inorganic particles. The samples were acetylated for 3–5 minutes to remove extraneous organic matter.

A light microscope was used to count pollen at a magnification of 400x. Pollen preservation in these samples was generally very good. Comparative reference material collected at the Intermountain Herbarium at Utah State University and the University of

Colorado Herbarium and published pollen literature on the southern cone of South America [19-20] was used to identify the pollen to the family, genus, and species level, where possible. Pollen aggregates were recorded during identification of the pollen. Aggregates are clumps of a single type of pollen and may be interpreted to represent either pollen dispersal over short distances or the introduction of portions of the plant represented into an archaeological setting. The aggregates were included in the pollen counts as single grains, as is customary. An “A” next to the pollen frequency on the percentage pollen diagram notes the presence of aggregates. A plus sign (+) on the pollen diagram indicates that the pollen type was observed outside the regular count while scanning the remainder of the microscope slide. The percentage pollen diagram was produced using Tilia 2.0 and TGView 2.0.2. Total pollen concentrations were calculated in Tilia using the quantity of sample processed in cubic centimeters (cc), the quantity of exotics (spores) added to the sample, the quantity of exotics counted, and the total pollen counted and was expressed as pollen per cc of sediment.

“Indeterminate” pollen was rare in these samples and includes pollen grains that were folded, mutilated, or otherwise distorted beyond recognition. These grains were included in the total pollen count since they are part of the pollen record. The microscopic charcoal frequency registers the relationship between pollen and charcoal. Although any starches present are preserved and recovered using our extraction process, none were observed while counting these samples.

*Phytoliths*

Extraction of phytoliths from these sediments also was based on heavy liquid flotation.

Quantities between 5 and 7.5 cc of sediment were placed in 600 ml beakers to which dilute HCl was added, then the samples were rinsed until neutral. The small samples were centrifuged (repeatedly) at high speed for short durations (15-20 seconds) to remove clay particles prior to heavy liquid separation. Next the samples were freeze- dried under vacuum. The dried silts and sands then were mixed with sodium polytungstate (density 2.1) and centrifuged to separate the phytoliths, which will float, from the other silica, which will not. After recovering the phytoliths, the phytolith-rich samples were rinsed with reverse osmosis deionized (RODI) water, then with alcohol to remove the water. After several alcohol rinses, the samples were mounted in immersion oil for counting with a light microscope at a magnification of 400x. A phytolith diagram was produced using Tilia and TGView 2.0.2.

*Phytolith Review*

Phytoliths are silica bodies produced by plants when soluble silica in the ground water is absorbed by the roots and carried up to the plant via the vascular system. Evaporation and metabolism of this water result in precipitation of the silica in and around the cellular walls. Opal phytoliths, which are distinct and decay-resistant plant remains, are deposited in the soil as the plant or plant parts die and break down. They are, however, subject to mechanical breakage, erosion, and deterioration in high pH soils. Phytoliths usually are introduced directly into the soils in which the plants decay. Transportation of phytoliths occurs primarily by animal consumption, gathering of plants by humans, or by erosion or transportation of the soil by wind, water, or ice. Phytoliths produced in roots/tubers will deteriorate at the level of those roots/tubers and will not be represented on the growing surface. Therefore, recovery of phytoliths representing roots/tubers from stratigraphic sediments does not necessarily represent vegetation coeval with that represented by phytoliths produced in leaves or other above-ground vegetative parts.

The three major types of grass short-cell phytoliths include festucoid, chloridoid, and

panicoid. Smooth elongate cylindrical phytoliths are of no aid in interpreting either

paleoenvironmental conditions or the subsistence record, because they are common in grasses. Phytoliths tabulated to represent "total phytoliths" include the grass short-cells,

buliform, trichome, elongate, and dicot forms. Frequencies for all other bodies recovered are calculated by dividing the number of each type recovered by the "total phytoliths".

The festucoid class of phytoliths is ascribed primarily to the subfamily Pooideae and occurs most abundantly in cool, moist climates. However, Brown [21] notes that festucoid (mostly rondel-type) phytoliths are produced in small quantity by nearly all grasses. Therefore, while they are typical phytoliths produced by the subfamily Pooideae, they are not exclusive to this subfamily. Chloridoid phytoliths (short saddles) are found primarily in the subfamily Chloridoideae, a warm-season grass that grows in arid to semi-arid areas and require less available soil moisture. Chloridoid grasses are the most abundant in the American Southwest [22] and other areas with hot, dry summers. Bilobates and polylobates (lobates) are produced mainly by panicoid grasses, although a few of the festucoid grasses also produce these forms. Panicoid phytoliths occur in warm-season or tall grasses that frequently thrive in humid conditions. Twiss [23] also notes that some members of the subfamily Chloridoideae produce both bilobate (panicoid) and festucoid phytoliths. “According to Gould and Shaw [22] more than 97% of the native US grass species (1,026 or 1,053) are divided equally among three subfamilies Pooideae, Chloridoideae, and Panicoideae” [23]. Buliform phytoliths are produced by grasses in response to wet conditions and are to be expected in wet habitats of floodplains and other places. They represent cells that control leaf rolling in response to drought. Trichomes represent silicified hairs, which may occur on the stems, leaves, and the glumes or bran surrounding grass seeds.

A rapidly growing body of phytolith studies, many of which use three-dimensional typological descriptions, allow a much finer resolution of taxa identification, often to the subfamily and genus level. This is especially true with the grass family, where diagnostic forms are now accepted for many additional subfamilies and genera such as:

Bambusoideae (bamboo), Erhartoideae (rice), tribe Oryzeae (Oryza, Zizania),

Aristidoideae (threeawn, needlegrass), Arundinoideae (reeds), and *Zea mays* (maize) [24]. Determining local and regional levels of diagnostic morphotypes can also greatly improve taxa identification.

*Other Siliceous Microfossils*

Diatoms and sponge spicules also were noted. Pennate diatoms are cosmopolitan, occurring in many sediments, and they indicate at least some soil moisture. Sponge spicules represent fresh water sponges. Diatoms are single-celled algae with a siliceous cell wall. They grow in a wide range of aerophilous habitats, including on wet plants and rocks, in damp soils, marshes, wetlands, mudflats, and all types of standing and flowing aquatic habitats. Their silica cells often are preserved in sedimentary deposits. Because individual taxa have specific requirements and preferences with respect to water chemistry, hydrologic conditions and substrate characteristics, the presence (and subsequent identification to the species level) of diatoms in paleoenvironmental context can provide information about the nature of the local environment. These data, coupled with input about local geology, hydrology, soil characteristics, pollen, and phytoliths, provide evidence of the paleoenvironmental setting. In the context of phytolith samples, diatoms are noted, but not identified beyond the split of “pennate” and “centric” forms. Centric diatoms often indicate wet conditions, while at least some of the pennate diatoms are cosmopolitan, occurring nearly everywhere. Both diatoms and sponge spicules can be transported with sediment. As an illustration, recovery of sponge spicules in upland soils is noted to accompany loess deposits derived from floodplains in Illinois [25].

**Discussion**

The pollen record was dominated by Nothofagus (southern beech trees) in samples 1 and 3 (S13a Fig.). Similar domination of the pollen record was noted by Heusser [26] for samples from the base of stratum MV-5 in the MV-II site, which dates to ~14,500 cal BP.

Sample 1, representing grinding stone no. 1 from the MV-II site, dated ~14,500 cal BP, displays a small quantity of *Jubea*-type pollen, suggesting wind transport of pollen from this member of the palm family. *Podocarpus* pollen also was present in a small quantity, representing an evergreen conifer. *Gevuina* pollen was observed, representing *avellano chileno* (chestnut), an evergreen tree that bears edible nuts that can be eaten raw or cooked by boiling in water or being toasted. Typically, these nuts are high in monounsaturated oils (49% oil) and contain about 12% protein and approximately 24% carbohydrates [27]. The shells also contain tannin. Small quantities of Brassicaceae, *Cleome*, and *Plantago* pollen might represent native plants whose seeds were ground using this grinding stone. Collecting seeds often also recovers pollen from the plants, as the plants usually continue to pollinate as the seeds develop and ripen. The moderate frequency of Poaceae pollen, accompanied by a single pollen aggregate, might represent grinding grass seeds. Large Poaceae pollen, tentatively identified as *Megalachne*-type, is approximately 50–60 microns in diameter and exhibits an annulated pore. *Megalachne* is a native grass of South America. Small quantities of *Astelia*-type, *Erodium*, *Polygonum* and *Sphaeralcea* pollen also were observed. Total pollen concentration was very high at almost 89,000 pollen per cc of the sediment, suggesting that this sample contained airborne pollen in addition to pollen representing use of the grinding stone.

The phytolith record for Sample 1 was dominated by buliform and rectangular buliform

phytoliths (S13b Fig.) representing grasses. A small quantity of spiny rectangular

buliforms also was noted. Small quantities of grass short cells were recovered. Forms representing festucoid or cool season grasses included rondels, pyramidal rondel, thin

rondel, Stipa-type bilobate, and trapezoid sinuate long.

A few short cells typical of chloridoid or short grasses were observed. Elongate cylindrical phytoliths, which are produced in many grasses, and a few trichomes, also produced in many grasses, were present. A few dicot forms, best described as angular and bulky, were observed in this sample. This phytolith record indicates the presence of grasses, quite possibly from the local sediments, rather than from the grinding surface.

Sample 2, representing grinding stone no. 2 from the MV-II site, did not display *Nothofagus* pollen. Instead, this grinding stone sample is a mixture of many pollen taxa, each of which is present as 20% or less of the total. *Podocarpus*, High-spine Asteraceae, Liguliflorae, Brassicaceae, *Plantago*, and Poaceae are the major pollen taxa. Of this list, it is possible that Brassicaceae, *Plantago*, and Poaceae pollen are present in part or in total as a result of grinding their seeds. In addition, a small quantity of large Poaceae pollen was observed, suggesting grinding the same grass seed (possibly *Megalachne*) mentioned for Sample 1. Small quantities of Myrtaceae pollen that was most similar to the genus *Ugni*, an evergreen shrub native to southern Chile, were noted in this sample and also in Sample 4. *Ugni* produces edible fruit, which might have been ground or processed on this grinding stone. At a minimum, the pollen signature indicates availability of this fruit. In addition, small quantities of *Gevuina*, *Lomatia*, *Artemisia*, Low-spine Asteraceae, Cheno- am, Cyperaceae, *Equisetum*-type, *Polygonum*, *Rumex*, and *Typha* pollen represent a collection of plants that grew locally, many of which were edible. Recovery of Cyperaceae, *Equisetum*, *Polygonum*, and *Typha* pollen suggests proximity to a wetland. It is possible this grinding stone was used to grind seeds and perhaps nuts from a variety of plants that included nuts from *Gevuina* and *Lomatia* and seeds from Cheno-ams, Cyperaceae, *Polygonum*, *Rumex* and *Typha*. Several spores representing ferns were noted in this sample. Total pollen concentration of more than 28,000 pollen per cc of sediment removed from the surface of this grinding stone suggests the presence of pollen derived from the surrounding sediments through natural deposition, including included wind transport.

The phytolith record in Sample 2 was very similar to that in Sample 1. Once again,

buliform and buliform rectangular phytoliths dominated the record. Festucoid or cool season grass short cells include rondel, rondel keeled, rondel pyramidal, and trapezoid sinuate long and short forms. Elongate forms, representing grasses, were present, but not particularly abundant, as were trichomes. Only a few dicot angular and blocky forms were observed. This record appears to be very generic and probably represents the local environment. No direct evidence of grinding was observed.

The phytolith record for Sample 3, representing an ash/charcoal stain (Unit 82, Feature 5), was unlike any other signature from this study. Many phytolith morphotypes were observed, although none were clearly dominant. Grass phytoliths that do not provide information concerning the type of grass present include buliforms, buliform rectangular, elongates, and trichomes. Grass short cells attributed to festucoid or cool season grasses include rondels, oval rondels, keeled rondels, pyramidal rondels, thin rondels, *Stipa*-type bilobate, and trapeziform sinuate long and short forms. Chloridoid forms, representing short grasses, were observed. Diatoms, spherasters and sponge spicules, representing organisms associated with water, also were present, but not abundant.

Sample 4 was collected from a burned feature (Unit 57, Feature 10) dated to ~18,578-

18,304 cal BP. This pollen signature was dominated by Liguliflorae pollen, suggesting a weedy field. Small quantities of Myrtaceae, *Nothofagus*, *Podocarpus*, Apiaceae, *Artemisia*, High-spine Asteraceae, Brassicaceae, Cyperaceae, *Plantago*, Poaceae, *Megalachne* grass, *Polygonum*, *Rumex*, and *Typha* *angustifolia*-type pollen document trees, shrubs, and herbaceous plants growing in the area. Fern spores were observed, but were not particularly abundant. Total pollen concentration was moderately high at nearly 36,000 pollen per cc of sediment.

The phytolith record from this feature exhibits larger quantities of buliforms and elongate phytoliths than were observed in the other burned feature at MV-I, but smaller quantities than were noted in the grinding stone samples from that MV-II. Small quantities of rondels, oval rondels, keeled rondels, pyramidal rondels, *Stipa*-type bilobates, trapeziform sinuate long, and trapeziform sinuate short forms represent festucoid or cool season grasses that probably grew in the area. It is interesting to note that the quantities of these short cells are not significantly different than those noted in the other thermal feature. Thin rondels are missing from this sample. A few dicot angular forms were observed, and diatoms were restricted to the ubiquitous pennate form, which is not necessarily strongly associated with water.

Sample 5, a very tiny quantity of sediment recovered from grinding stone no. 3 at MV-II, yielded no pollen. The phytolith record, however, is similar to the signatures obtained from the thermal feature at Monte Verde I. Buliform and buliform rectangular forms were abundant, as were elongates, all of which represent grasses. Small quantities of rondels, oval rondels, keeled rondels, pyramidal rondels, thin rondels, *Stipa*-type bilobates, and trapeziform sinuate long and short forms represent festucoid or cool season grasses. Chloridoid forms, representing short grasses, are present. Only a few bilobate forms were noted, which might represent tall grasses or might have been produced in cool season grasses. Dicot bulky and parallelepiped forms indicate that the phytolith record documented at least a few non-grass plants. This sample also yielded a rodent hair fragment and a starch typical of many types of grass seeds, but diagnostic of none. Pennate diatoms were more abundant than phytoliths in this sample, probably representing the sediment conditions in which this grinding stone was buried. Alternatively, it is possible that something collected from an environment that supported these organisms was ground using this grinding stone.

**Summary and Conclusions**

Pollen, phytolith, and starch analysis of five samples submitted from sites MV-I and MV-II yielded evidence of the local environment during the time period represented. The samples included sediments recovered from the surface of three grinding stones and fill from two thermal features representing occupation approximately ~16,000-14,500 cal BP. The local setting represented in the samples includes *Nothofagus* and *Podocarpus* trees, as well as a few *Jubea*, Myrtaceae, *Gevuina*, and *Lomatia* trees. Plants in the sunflower family appear to have been common, with those in the chicory tribe most common locally. It is likely that a plant in the Apiaceae family grew as part of the weedy plant complex, probably in moist ground. Brassicaceae pollen was noted in the sample from two grinding stone samples examined from MV-II. It appears to have been part of the native landscape and likely was ground using two of the grinding stones. Cyperaceae pollen, representing sedges, was observed in one grinding stone from MV-II and one burned feature from MV-I. This distribution indicates the presence of sedges on the landscape spanning many thousands of years. It is likely they were processed using grinding stones and possibly cooked in the thermal feature. It is interesting to note that no Cyperaceae phytoliths were observed in any of these samples. *Typha* pollen was noted in Sample 2, representing one of the grinding stones from MV-II. The large quantity observed in the grinding stone samples could easily represent grinding cattail seeds. Other evidence for the presence or local growth of wetland plants includes recovery of *Equisetum*-type pollen from the grinding stone that yielded the cattail pollen.

Recovery of *Polygonum* pollen from two grinding stone samples suggests processing a member of this genus during the occupation of MV-II. Likewise, recovery of *Rumex* pollen from one grinding stone sample suggests grinding of an edible portion of the plant during the 14,500 cal BP occupation at MV-II. *Rumex* or dock leaves and stems may have a bitter taste. Leaves and stems of all species are best harvested and processed when young. Often the leaves and stems are boiled.

Diatoms, spherasters, and/or sponge spicules were observed in samples from a thermal feature in MV-I and especially one of the grinding stones from MV-II. The two sediment samples yielded small quantities of diatoms and the occasional spheraster and sponge spicule, which suggests that the sediments were not overly wet. Total pollen concentration in samples representing two grinding stones at MV-II and one thermal feature, was high to very high, ranging from approximately 28,700 to 187,700 pollen per cc of sediment. In the four samples that yielded pollen, the exception being the tiny sample representing a grinding stone from MV-II, the pollen was in an excellent state of preservation, indicating that should future analysis be undertaken. Results can be expected to be similarly productive. The absence of pollen in Sample 5 might be related to the fact that this was the smallest sample submitted, or it might reflect post-depositional conditions that were significantly different.


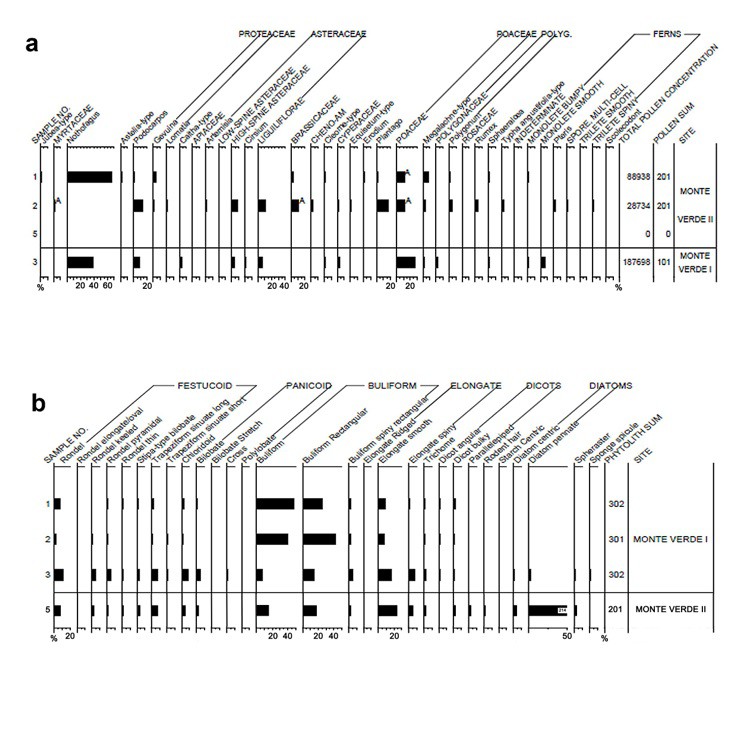


S13 Figure. Pollen and phytolith profiles from sediment samples from the MV-I and MV-II sites.

**Text G. Luminescence Dating of Sediments**

Andre Oliveira

Sawakuchi

Quartz and K-rich feldspar grains were used for luminescence dating [28] of the Monte Verde sediments. Only five samples presented quartz concentrates with amounts suitable for OSL dating. K-rich feldspar concentrates were acquired for all studied samples. Infrared Stimulated Luminescence (IRSL) and post-IRIR signals of K-rich feldspar concentrates measured using a protocol with high temperature preheat [29-30] were tested for equivalent dose estimation. The post-IRIR signal showed significant residual doses compared to the natural doses range and it was unsuitable for dating of the target sediments. However, the first IR stimulation at 50oC (IR50) in the post-IRIR protocol allowed determination of reliable equivalent doses as indicated by dose recovery tests. Thus, quartz OSL ages and K-rich feldspar IR50 ages derived from the high temperature post-IRIR protocol were combined to define the chronology of sediment deposition of the Monte Verde site. K-rich feldspar IR50 ages corrected for athermal fading and quartz OSL ages are compatible with 14C ages of correlated stratigraphic units (see Table 1 and Text B). The following sections present detailed descriptions of the methods used for equivalent dose and dose rate determination, the obtained equivalent doses, dose rates and ages and a discussion of age uncertainties.

**Methods**

*Sample Preparation*

Samples were collected in aluminum or opaque PVC tubes for luminescence measurements. Most of the samples were coarse-grained sands with gravel or muddy sands. Sample preparation included wet sieving to separate the 180-250 µm grain size fraction, which was submitted for chemical treatments (H2O2 and HCl 10%) to eliminate organic matter and carbonates, followed by heavy liquid separation using lithium metatungstate (LMT) solution at densities of 2.85 and 2.62 g/cm3. Grains with densities between 2.85 and 2.62 g/cm3 and lower than 2.62 g/cm3 were studied under the microscope to evaluate their mineralogical composition. Both density fractions showed grains of quartz, feldspar and a significant amount of lithic fragments composed of feldspar, quartz, mica and volcanic materials. The quartz concentrates (2.62-2.85 g/cm3) were etched for 40 min with HF 40% to improve quartz concentration and eliminate the outer layer of grains damaged by alpha particles. The fraction with a density lower than 2.62 g/cm3 was dominated by feldspar, despite the presence of lithic fragments and quartz. This fraction was etched for 40 min with HF 10% to reduce the amount of unstable lithic fragments and eliminate the outer layers of feldspar grains damaged by alpha particles. Quartz and feldspar concentrates were sieved again after HF etching. X-ray diffraction analyses of aliquots of samples MV-II-D6 and MV-I-45(A)-1 were performed after HF etching, confirming the predominance of feldspar in the fraction with density lower than 2.62 g/cm3, but also indicating the presence of quartz and mica. The study of samples under the microscope indicated the persistence of lithic fragments composed of feldspar and quartz. The heavy liquid separation using LMT at density of 2.58 g/cm3 was not performed due to the low content of grains in most of the samples and because this procedure was unsuitable for isolating K-rich feldspar grains in the studied sediments. Despite the possible presence of grains of albite (2.60-2.65 g/cm3) in the fraction lighter than 2.62 g/cm3, the prepared aliquots are considered concentrates of K-rich feldspar grains.

*Luminescence Measurements*

Multigrain aliquots were mounted in steel cups for measurements in two Risø TL/OSL DA-20 readers (Risø National Laboratory) equipped with IR diodes (875nm) and blue LEDs (470nm) for stimulation and built-in 90Sr/90Y beta sources delivering, respectively, 0.084 and 0.119 Gy/s to the samples. The aliquots comprised a few hundred of grains (100-300 grains). Grains were spread within the cups to ensure a monolayer. Luminescence measurements of feldspar grains were performed using infrared (IR) stimulation and the Schott BG39/Corning 7-59 filters pack for signal detection in the blue-violet region. Hoya U-340 filter (290-370nm) was used for signal detection of quartz aliquots. IR stimulation indicated aliquots with a high natural signal and an IRSL decay curve with relatively slow decay, which is typical of feldspar (S14a Fig.). It was not possible to isolate pure grains of K-feldspar, thus, we assumed an IRSL signal dominated by K-rich feldspar, considering its brighter character and higher light emission in the blue wavelength region [31-33]. Single-Aliquot Regenerative (SAR) dose protocol [28] based on the post-IRIR at 290oC (pIRIR290) signal [29,30] was tested for equivalent dose measurements in K-rich feldspar concentrates. The signal derived from the first IR stimulation at 50oC in the pIRIR290 protocol was also tested for equivalent dose estimation. The pIRIR290 should be a bleachable K-feldspar signal without anomalous fading [29-30, 34-35]. A dose recovery test was carried out to evaluate the Monte Verde quartz and K-rich feldspar concentrates as radiation dosimeters. The dose recovery test was performed in 9 aliquots of quartz and 9 aliquots of K-rich feldspar of samples MV-II-D1 and MV-I-45-2, using the protocols presented in S4 Table. For the dose recovery test, the quartz aliquots were bleached under a solar simulator for 4 hours before irradiation with known beta doses of 11.9 Gy and 35.7 Gy. Preheat temperatures from 200oC to 260oC were tested for equivalent dose estimation using quartz. The aliquots of K-rich feldspar were bleached with IR stimulation for 200s at 50oC and 200s at 290oC before irradiation with a known beta dose of 16.8 Gy. Quartz OSL signal and background were calculated respectively through the integral of the first 1.5s and last 10s of the OSL decay curve. For K-rich feldspar aliquots, the IR50 signal and background were calculated respectively by integrating the first 3.2s and last 20s of the IRSL decay curve. Considering that the dose recovery used artificial bleached aliquots of K-rich feldspar, its results only evaluated the capacity of the protocol in correct changes of luminescence sensitivity and in estimating a known radiation dose. Feldspar aliquots were not bleached under the sunlight for the dose recovery test. Thus, an experiment with exposure of feldspar aliquots under a solar simulator during increasing times was performed to evaluate the time for depletion of the natural IR50 and pIRIR290 signals. The bleaching test comprised the measurement of the natural signal depletion in aliquots exposed to a solar simulator lamp during periods of 2, 4, 8, 24 and 48 hours.

Only aliquots with a recycling ratio between 0.9 and 1.1 and recuperation less than 5% were used for equivalent dose calculation. The equivalent dose was determined by linear fitting of the dose-response function for quartz aliquots in the range from 1 to 10 Gy. The equivalent doses of quartz aliquots with higher doses and feldspar aliquots were determined using an exponential fitting of the dose-response function. Equivalent dose distributions were presented in radial plots. For each sample, equivalent dose and its error for age calculation were determined through the Central Age Model [36] using the Luminescence Analyst software (version 4.12). Feldspar IR50 ages were corrected for signal fading [37-38]. The g values (% of signal decay/decade) for fading correction of ages estimated using the IR50 signal were measured for samples MV-I-45-1, MV-II-D1, MV-II-D6 and MV-II-D7 using the procedures proposed in Auclair [38]. These samples are considered representative of the studied samples. Measurements of fading rate used three aliquots per sample. Fading rate was evaluated through the decay of a corrected signal measured for a dose of 12 Gy and a test dose of 6 Gy. Delay times for evaluation of signal decay were 1, 2, 3, 6, 12 and 18 hours. Preheat was applied immediately after irradiation. IR50 corrected signals were normalized by a first corrected signal measured immediately after irradiation and preheat. Delay times were normalized by a critical time (t*) of 2 days.

*Dose Rate Determination*

Samples for gamma ray spectrometry and radiation dose rate determination were collected at the same positions where each luminescence sample was taken. Samples were weighed and dried to measure the water content. Gamma spectrometry was achieved after 28 days of samples packed in sealed containers for radon equilibrium. Gamma spectrometry was performed with a high purity germanium detector (HPGe) mounted in an ultralow background shield. The HPGe used has an energy resolution of 2.1 KeV and a relative efficiency of 55% (Canberra Industries). Gamma-ray spectrums were acquired during 24 hours. The activities of 4 0 K and of 23 8 U and 23 2 Th daughter nuclides were calculated through an efficiency calibration based on ISOCS (In Situ Object Counting System) for the used HPGe detector. Terrestrial radiation dose rates were calculated by using concentrations of 4 0 K, 23 8 U and 23 2 Th and conversion factors [39]. Huntley and Baril [40] report potassium contents from 6.5 to 16.9% in K-feldspar. The average value of 12.5±0.5% suggested by Huntley and Baril [40] was used for calculation of internal dose rate of the K-rich feldspar concentrates extracted from the Monte Verde sediments. The water saturation (water mass to dry sample mass in weight percent) of each sample during the time of collection was considered for dose rate calculation. However, an absolute uncertainty in water percentage (±10%) was used due to the proximity of the samples to the water table and to difficulties in evaluating changes in water saturation through time. The cosmic radiation dose rate was determined through the sample’s latitude, longitude, altitude, burial depth and density [41]. The total error of the dose rate and ages were calculated according the Gaussian law of error propagation.

**Results**

*Bleaching Experiment and Dose Recovery Test*

The S14 Fig. shows the luminescence decay and dose response curves for quartz and K-rich feldspar concentrates from the Monde Verde sediments. K-rich feldspar present high luminescence sensitivity that favors equivalent dose measurements, especially in mountain or glacial sedimentary environments where recently eroded quartz grains commonly have poor luminescence characteristics [33,42]. However, the longer bleaching time of feldspar IR50 and pIRIR290 signals compared to quartz OSL signal can generate significant residual doses and age overestimation of Holocene and late Pleistocene sediments [30]. Thus, a bleaching test is recommended for evaluation of K-rich feldspar from each geological setting. The decay of the natural IR50 and pIRIR290 signals measured in aliquots (sample MV-I-45(A)-2) with increasing bleaching times is showed in S15 Fig. The experiment indicates that the pIRIR290 signal requires a relatively long time to complete bleaching, presenting a very high residual signal (40% of the initial natural signal) after 24 hours of light exposure. This signal corresponds to equivalent doses of tens of Gy in the Monte Verde samples. Thus, residual pIRIR290 signals would generate an age overestimation of tens of thousands years for dose rates of 1 to 2 Gy/ka. Complete bleaching of pIRIR290 signal is unsuitable in glaciofluvial sedimentary environments, which are commonly characterized by short-time exposure to sunlight due to fast erosion and deposition events. A relatively fast bleaching was observed for the feldspar IR50 signal (S15 Fig.). This signal decays to 20% of the initial natural signal in 4-8 hours of light exposure. After 48 hours of light exposition, the remaining signal is around 10-15% of the initial signal. According to the bleaching test, the feldspar IR50 signal is more suitable than the pIRIR290 signal for dating of feldspar grains from the Monte Verde sediments.

Reliable dose recovery results for quartz aliquots were achieved with a preheat temperature of 200oC. Calculated to given dose ratios were 0.92±0.05 (9 aliquots with overdispersion of 15%) and 0.92±0.04 (4 aliquots with overdispersion of 5%) for given doses of 35.7 Gy and 11.9 Gy, respectively. In the dose recovery test of K-rich feldspar, the calculated to given dose ratio was 0.93±0.06 for the IR50 signal. The IR50 signal of K-rich feldspar concentrates from sample MV-I-45-2 recovered reliable equivalent doses, with overdispersion of 10.4% (5 aliquots accepted among 9 measured aliquots). The pIRIR290 signal presented a calculated to given dose ratio of 0.95±0.02, with overdispersion of 4.3% (6 aliquots accepted among 9 measured aliquots). The dose recovery test indicates that the SAR protocols used in this study are suitable to correct changes in luminescence sensitivity and to recover a known dose using the OSL signal of quartz and the IR50 and pIRIR290 signals of K-rich feldspar from the Monte Verde sediments. However, the high residual doses detected for the pIRIR290 signal hindered its use for dating of the Holocene and late Pleistocene sediments from the MV-II site. Thus, the luminescence chronology of the MV-II site was based on the combination between quartz OSL and K-rich feldspar IR50 ages.

*Fading Rate*

The S16 Fig. shows the variation of corrected IR50 signals measured for increasing delay times between irradiation (12 Gy) and IR stimulation. The analyzed samples presented fading rates from 0.2 to 1.8%/decade (g values). The signal increase observed in sample MV-II-D6 can be attributed to changes in sensitivity that occurred during the SAR cycles for fading rate measurement. However, the variations are very small, suggesting relative stability for the IR50 signal of this sample. An average fading rate of 1.12±0.17%/decade was used for correction of IR50 ages. These g values are below average g values reported in the literature for the IR50 signal [37-38]. However, similar g values for the IR50 signal were also reported for K-feldspar from other regions [29, 34, 37].

*Equivalent Doses, Dose Rates and Ages*

Luminescence signals of quartz and K-rich feldspar aliquots grow with radiation doses (S14 Fig.). Equivalent dose distributions of K-rich feldspar aliquots have overdispersion ranging from 10% to 37% (S17 Fig. and S6 Table). Quartz aliquots presented equivalent dose distributions with higher overdispersion, ranging from 35 to 84% (S6 Table). Many quartz aliquots were rejected due to feldspar contamination or low sensitivity and poor dose response curves, especially for samples from stratigraphic Unit D. According to Arnold and Roberts [43] equivalent dose distributions of well-bleached sediments can present overdispersion up to 35%. The equivalent dose distributions of site sediments are well described by a single dose peak. The equivalent dose of each sample was represented by a central dose value. Dose rates varied from 0.52±0.03 to 1.14±0.08 Gy/ka and from 1.35±0.07 to 1.88±0.09 Gy/ka, respectively for quartz and K-rich feldspar grains. Internal dose rate represents between 39 and 55% of the total dose rate of K-rich feldspar grains. The S5 Table summarizes the dose rate data for the studied sediments.

The feldspar IR50 ages corrected by a g value of 1.12±0.17%/decade are in agreement with most quartz OSL ages from the river bank profile and with 14C ages presented in this study and in previous studies [1-2, 5, 44-45]. The S6 Table shows the ages obtained for quartz and K-rich feldspar concentrates from the Monte Verde sediments. Sample locations and ages are indicated in the profiles of S18 Fig. According to luminescence ages of quartz and K-rich feldspar concentrates, up to four stratigraphic units (A-D) are recognized in the Monte Verde site. These units are better represented in the creek bank profile of the MV-II site (S18 Fig.). Unconformities separate Units B from C and Units C from D. Sediment composition, magnetic and microstratigraphy data support the unconformities indicated by luminescence ages. The unconformity separating Units C and D was also identified in the profile MV-I-45(A) (S18 Fig.). In this site, Unit C is younger than 14,118±1,291 years (sample MV-I-45(A)-1) and Unit D is older than 34,524±2,973 years (sample MV-I-45(A)-2). The base of Unit C overlying MV-II is younger than 13,793±906 years and formed after the MV-II site. This age agrees with the 14C dates of MV-II habitation layer that rests on the buried surface of stratum MV-7 (Unit D) and covered and sealed by stratum MV-5, the peat layer that underlies Unit C. Both the radiocarbon and OSL dates indicate that any cultural materials buried below the upper levels of stratum MV-7 date prior to 15,000 years ago.

**Discussion**

*Stratigraphy and Sediment Deposition*

Stratigraphic Units A and B (S18 Fig.) are dominated by fine sand with sparse granules. Units C and D are composed of poor sorted sand with gravel (granules to pebbles). Despite their similar texture, Units C and D exhibit contrasting composition. Unit D is more immature, with high content of labile clasts of volcanic rocks. Unit D is also more compacted, suggesting a higher degree of pedogenesis compared to the overlaying units. The magnetic and microstratigraphy data support the interpretation of higher degree of pedogenesis for Unit D and a major unconformity separating Units D and C.

A short time hiatus could occur between deposition of Units B and C (profile in MV-II). Luminescence ages from the top and base of the bed units are overlapped by errors, indicating abrupt episodes of sediment accumulation, possibly related with high precipitation events. The higher compositional maturity and multiple events of deposition for the interval comprising Units A, B and C point to a wetter period and the Chinchihuapi Creek with a more frequent water flow and vegetated floodplain able to promote soil development. This is in agreement with the sedimentological, magnetic and microstratigraphical data, which respectively indicate that sediments from Units B and C (post-LGM) were generated under wetter climate compared to the sediments of Unit D (MIS-2).

*Luminescence Age Uncertainties*

Despite the moderate to low sensitivity of quartz from Monte Verde sediments, reliable quartz OSL ages were acquired for five sediments samples from the riverbank MV-II profile. Quartz OSL ages are in agreement with feldspar IR50 ages obtained in the same sediment samples of profile MV-II, validating the luminescence chronology of profile MV-I, which was exclusively based on feldspar IR50 ages. Despite the agreement between quartz and feldspar ages, possible uncertainties regarding the feldspar IR50 ages are discussed. The characteristics of the studied samples precluded the preparation of pure aliquots of K-feldspar grains. Thus, sodium feldspar could eventually contributed to the IRSL signal used for determination of equivalent dose. In this case, an age underestimation would be expected due to the lower potassium content. Considering a value of 10% for the potassium content in K-feldspar (instead of 12.5±0.5% used as an average standard value), the total dose rate would be around 8-10% lower than the values used for ages calculation. However, most feldspar ages calculated using a potassium content of 8-10% would be within the errors of the presented IR50 ages.

The average fading rate of 1.12±0.17%/decade measured for the K-rich feldspar extracts from the Monte Verde area is relatively low compared to average values from the literature, but already observed in other settings [29,34,37]. The use of high temperature preheat (320oC for 60s) could access an IRSL signal measured at 50oC with relatively higher stability and lower athermal fading. This is suggested in Buylaert [29], which show a decrease in fading rate from 4.13±0.10 to 2.4±0.4%/decade due to an increase in the preheat temperature from 150 to 250oC (60s). The Monte Verde feldspar grains show an IR50 signal corresponding to equivalent doses of few Grays after 48 hours of exposition to a solar simulator. Considering that incomplete bleaching is a common characteristic of fluvial deposited sediments, residual signals could generate some age overestimation. However, most of the dated samples have equivalent dose distributions with low to moderate overdispersion, suggesting that incomplete bleaching is not a major problem.

All studied sediment samples are a few centimeters to a few meters from the water table. Thus, variation of water saturation and changes in the external dose rate through time are expected. The sedimentary Unit D would be more influenced by changes in dose rate through time, considering it experienced a shift from drier to a wetter climate. This climate shift would increase water saturation through time and could provoke an underestimation of the dose rate based on present water saturation.

The uncertainties regarding K-rich feldspar internal dose rate, fading rate measurement, incomplete bleaching and dose rate variation due to changes in water saturation are limitations of the luminescence dating methods so far. Despite the discussed uncertainties, the integration of quartz OSL, feldspar IR50 and 14C ages allow us to constrain a reliable chronology for sediment deposition and the associated archaeological horizons in the Monte Verde area.


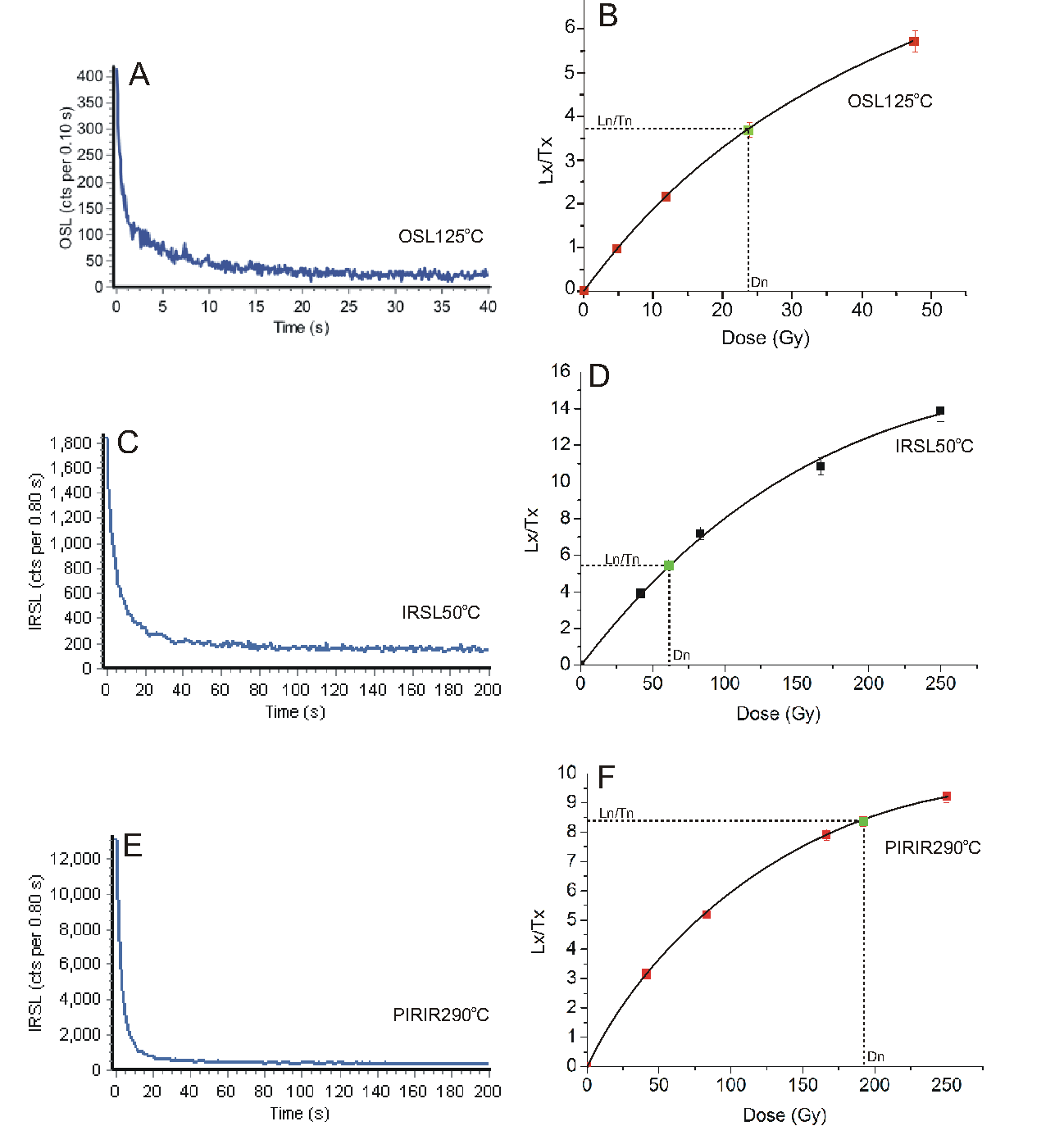


S14 Figure: Shine-down and dose response curves of quartz aliquot from sample MV-II-D7 (A and B). Equivalent dose was 22.81±1.80 Gy. Shine-down and dose response curves of the IR_50_ (C and D) and pIRIR_290_ (E and F) signals measured in K-rich feldspar aliquot from sample MV-I-45(A)-2. Equivalent doses for this aliquot were 60.38±3.91 Gy (IR_50_) and 192.01±11.42 Gy (pIRIR_290_). The green square indicate natural signal (Ln/Tn) and equivalent dose (Dn).


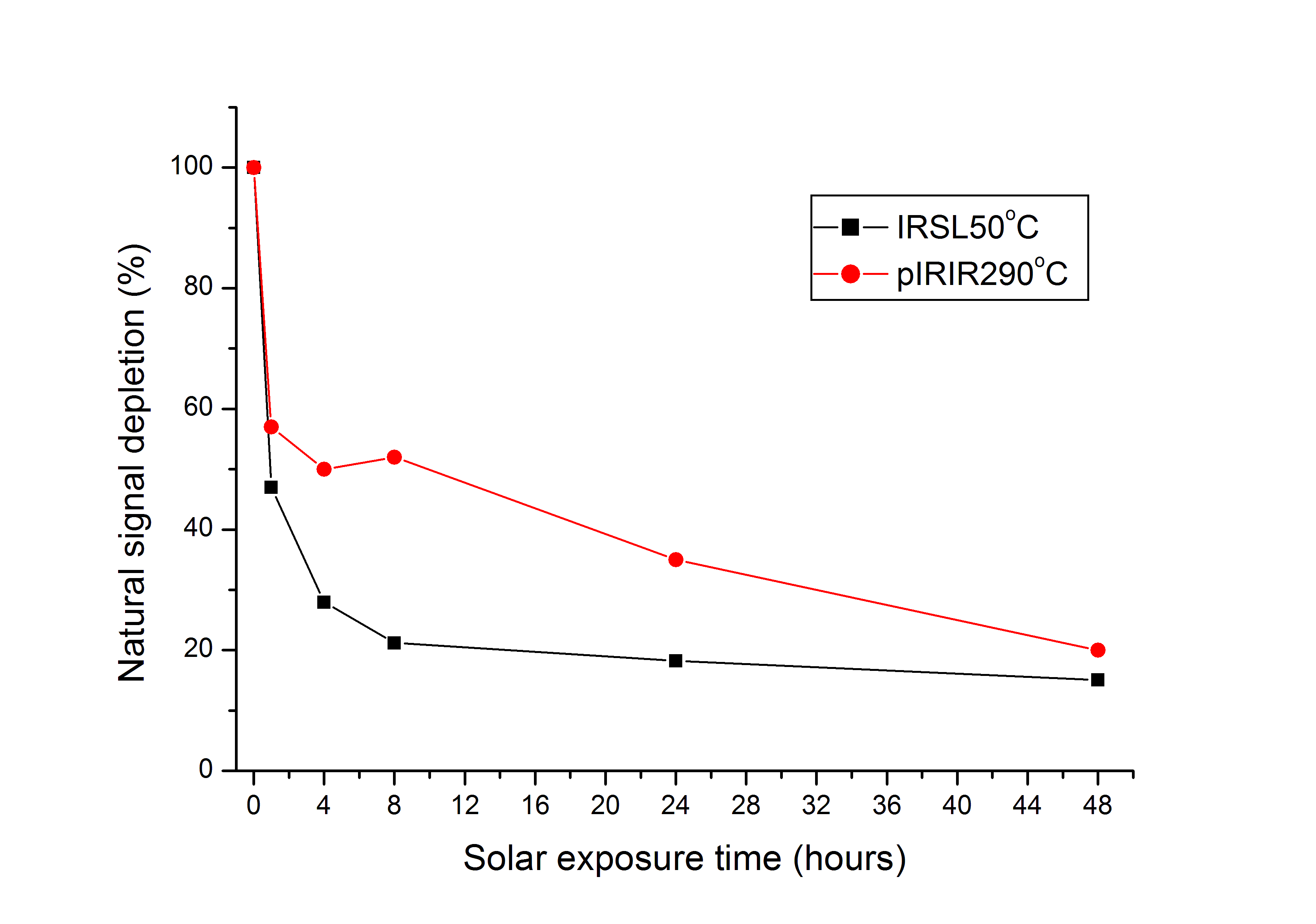


S15 Figure: Decay of the natural IR_50_ and pIRIR_290_ signals (corrected by a test dose signal) of aliquots (sample MV-I-45(A)-2) exposed to increasing bleaching times. The signal decay is represented as a percentage of the natural signal. The natural IR_50_ and pIRIR_290_ signals respectively correspond to equivalent doses of approximately 58 Gy and 221 Gy.


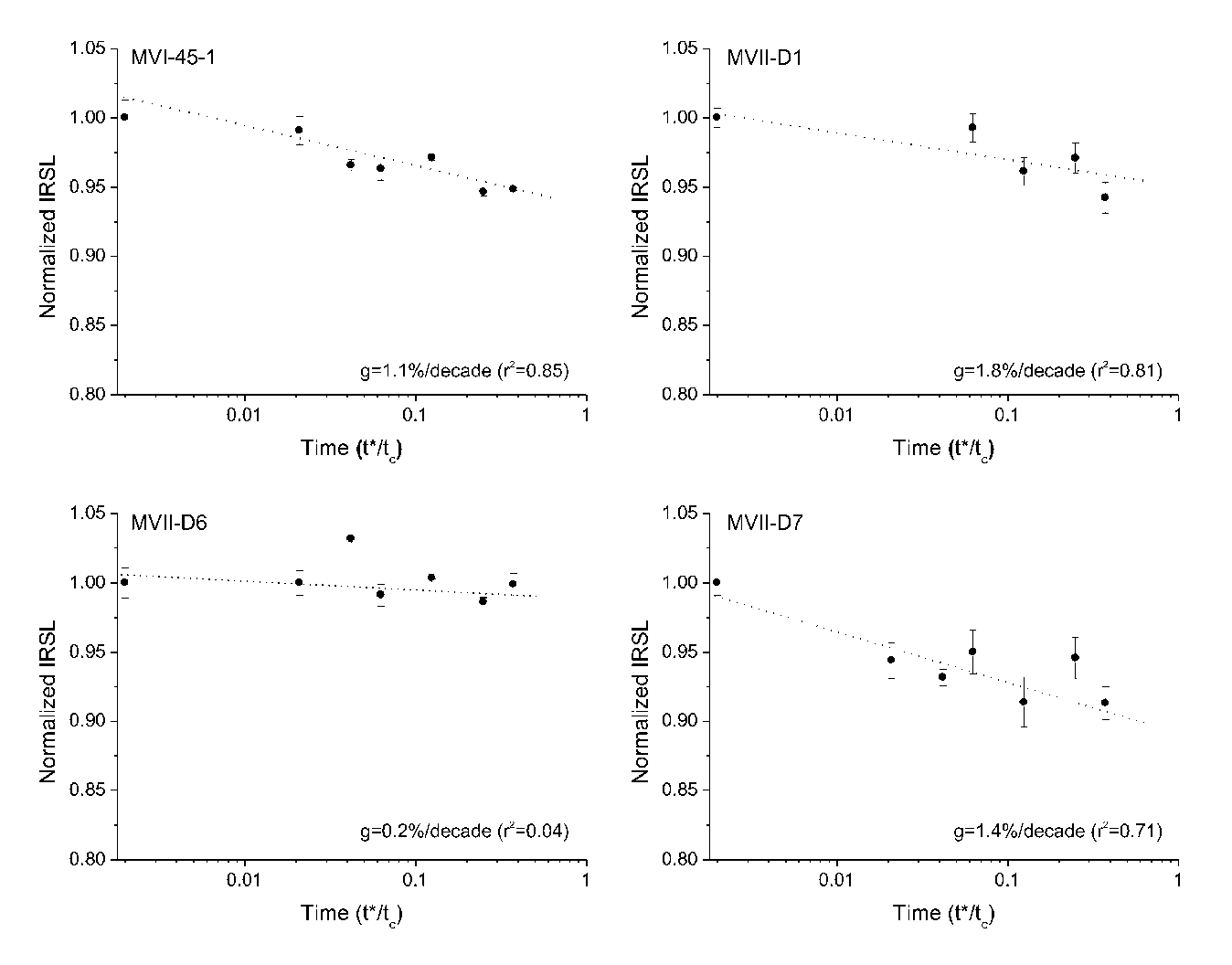


S16 Figure: Athermal decay of the corrected IR_50_ signal of samples MV-I-45(A)-1, MV-II-D1, MV-II-D6 and MV-II-D7. Each point corresponds to the average of three aliquots. Aliquots were irradiated with a beta dose of 12 Gy. Signals are normalized to the signal measured without delay time between irradiation and infrared stimulation. Delay times were normalized to a critical time of 2 days (t*).


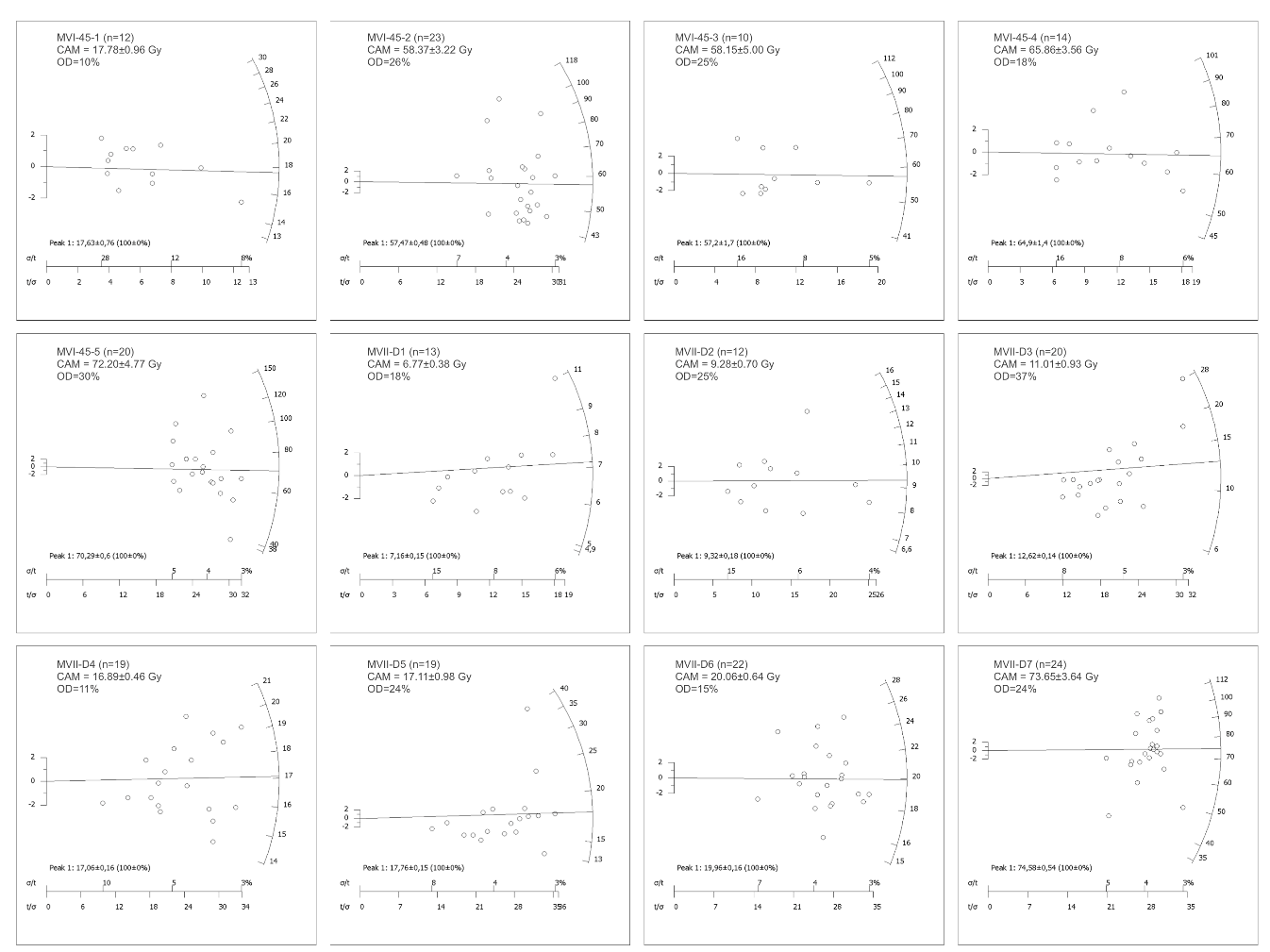


S17 Figure: Radial plots of equivalent dose distributions measured in K-rich feldspar using the IR_50_ signal. Equivalent doses are not corrected for signal fading.


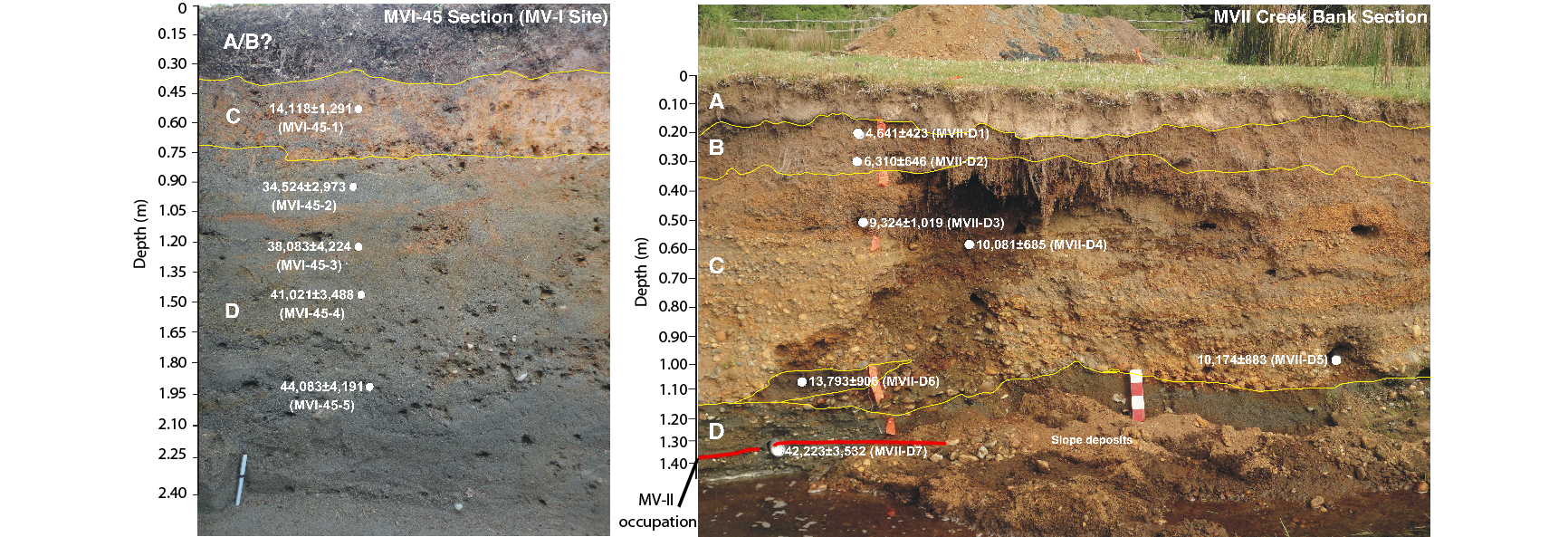


S18 Figure: Luminescence ages of K-rich feldspar grains in the MV-I-45(A) profile and in the river bank profile of the MV-II site. A, B, C and D indicate the proposed stratigraphic division. Two unconformities are recognized in the riverbank profile, separating three stratigraphic units. The longer time interval unconformity covers the Last Glacial Maximum (LGM) period and separate Unit C (younger than 13,793±906 years) from Unit D (older than 34,524+/-2,973 years). The orangish sediments at bottom of profile in the MV-II site are slumped deposits from Unit C.

S4 Table: SAR protocols used for equivalent dose determination in quartz and K-rich feldspar aliquots. Equivalent doses of K-rich feldspar aliquots were determined through the first IRSL stimulation at 50^o^C (IR_50_) corrected by the test dose signal. D0 corresponds to the natural radiation dose. D1, D2, D3 and D4 were used to build the dose-response curve. D5 (0 Gy) was used to calculate recuperation. D6=D1 was employed to calculate recycling ratio. For quartz aliquots, IRSL at 50^o^C was performed before blue stimulation to measure the signal of D_7_. The recycling ratio between the corrected signals of D_6_ and D_7_ was used to check feldspar contamination in quartz aliquots.

| **Step** | **Procedure (Quartz)** | **Procedure (K-rich feldspar)** |
| --- | --- | --- |
| 1. | Dose (D_0_, D_1_, D_2_, D_3_, D_4_, D_5_, D_6_=D_7_=D_1_) | Dose (D_0_, D_1_, D_2_, D_3_, D_4_, D_5_, D_6_=D_1_) |
| 2. | Preheat (200^o^C for 10s) | Preheat (320^o^C for 60s) |
| 3. | OSL (Blue LEDs), 40s at 125^o^C | IRSL, 200s at 50^o^C |
| 4. | Test dose | IRSL, 200s at 290^o^C |
| 5. | Cut heat (160^o^C) | Test dose |
| 6. | OSL, 40s at 125^o^C | Preheat (320^o^C for 60s) |
| 7. | Blue bleach for 100s at 280^o^C | IRSL, 200s at 50^o^C |
| 8. | Return to step 1 | IRSL, 200s at 290^o^C |
| 9. |  | IRSL, 200s at 325^o^C |
| 10. |  | Return to step 1 |

S5 Table: Radionuclides concentrations and dose rate data for quartz and K-rich feldspar grains.

| **Profile** | **Sample** | **Depth (m)** | **Water saturation (%)** | **U (ppm)** | **Th (ppm)** | **K (%)** | **External gamma dose rate (Gy/ka)** | **External beta dose rate (Gy/ka)** | **Internal dose rate (Gy/ka)** | **Cosmic dose rate (Gy/ka)** | **Total dose rate for K-rich feldspar (Gy/ka)** | **Total dose rate for quartz (Gy/ka)** |
| --- | --- | --- | --- | --- | --- | --- | --- | --- | --- | --- | --- | --- |
| MVI-45 | MVI-45-1 | 0.37 | 71±10 | 0.58±0.01 | 3.24±0.01 | 0.40±0.02 | 0.17±0.01 | 0.24±0.02 | 0.74±0.05 | 0.20±0.03 | 1.35±0.07 | * |
|  | MVI-45-2 | 0.63 | 22±10 | 0.77±0.01 | 5.09±0.01 | 0.57±0.02 | 0.37±0.03 | 0.51±0.05 | 0.74±0.05 | 0.19±0.03 | 1.82±0.09 | * |
|  | MVI-45-3 | 0.82 | 31±10 | 0.58±0.01 | 2.85±0.01 | 0.63±0.02 | 0.26±0.02 | 0.45±0.04 | 0.74±0.05 | 0.19±0.03 | 1.65±0.08 | * |
|  | MVI-45-4 | 0.97 | 22±10 | 0.60±0.01 | 2.87±0.01 | 0.66±0.03 | 0.29±0.03 | 0.51±0.05 | 0.74±0.05 | 0.19±0.02 | 1.73±0.08 | * |
|  | MVI-45-5 | 1.28 | 16±10 | 0.65±0.01 | 3.33±0.01 | 0.61±0.03 | 0.32±0.03 | 0.52±0.06 | 0.74±0.05 | 0.18±0.02 | 1.77±0.09 | * |
| River bank | MVII-D1 | 0.20 | 13±10 | 0.42±0.01 | 1.88±0.01 | 0.47±0.01 | 0.22±0.02 | 0.39±0.04 | 0.74±0.05 | 0.20±0.05 | 1.55±0.09 | 0.81±0.07 |
|  | MVII-D2 | 0.30 | 17±10 | 0.49±0.01 | 2.58±0.01 | 0.46±0.01 | 0.24±0.02 | 0.39±0.04 | 0.74±0.05 | 0.20±0.04 | 1.57±0.08 | * |
|  | MVII-D3 | 0.50 | 37±10 | 0.31±0.01 | 1.58±0.01 | 0.29±0.01 | 0.13±0.01 | 0.20±0.02 | 0.74±0.05 | 0.19±0.02 | 1.26±0.06 | 0.52±0.03 |
|  | MVII-D4 | 0.60 | 11±10 | 0.64±0.01 | 3.33±0.01 | 0.59±0.02 | 0.33±0.03 | 0.53±0.06 | 0.74±0.05 | 0.19±0.02 | 1.79±0.09 | * |
|  | MVII-D5 | 1.00 | 15±10 | 0.65±0.01 | 3.15±0.01 | 0.66±0.03 | 0.33±0.03 | 0.55±0.06 | 0.74±0.05 | 0.18±0.02 | 1.80±0.09 | 1.06±0.07 |
|  | MVII-D6 | 1.10 | 41±10 | 0.61±0.01 | 3.10±0.01 | 0.59±0.03 | 0.24±0.02 | 0.39±0.04 | 0.74±0.05 | 0.18±0.01 | 1.56±0.07 | 0.82±0.04 |
|  | MVII-D7 | 1.40 | 13±10 | 0.67±0.01 | 3.08±0.01 | 0.74±0.03 | 0.35±0.03 | 0.62±0.07 | 0.74±0.05 | 0.17±0.01 | 1.88±0.10 | 1.14±0.08 |

Water saturation calculated as the percentage of water weight in relation to dry sample weight.

S6 Table. Equivalent doses data, quartz OSL ages and IR_50_ K-rich feldspar ages (non-corrected and corrected for fading) determined for the Monte Verde sediments. Dose rate data are in Table S5. The ^14^C ages were determined for charcoal and algae fragments [44, 45] within beds correlated with the base of stratigraphic Units C and top of stratigraphic Units D (see profiles in S18 Fig.).

| **Profile** | **Units** | **Sample** | **Depth (m)** | **K-rich feldspars** | | | | | **Quartz** | | | | **^14^C ages**  **(cal years BP)** |
| --- | --- | --- | --- | --- | --- | --- | --- | --- | --- | --- | --- | --- | --- |
|  |  |  |  | **De (Gy)** | **Number of aliquots** | **OD (%)** | **Non-corrected IR_50_ ages (years)** | **Corrected IR_50_ ages (years)**  **g_2days_ = 1.12±0.17%/decade** | **De (Gy)** | **Number of aliquots** | **OD (%)** | **Ages (years)** |  |
| MVI-45 | C | MVI-45-1 | 0.37 | 17.78±0.96 | 12 | 10 | 13,161±1,048 | 14,118±1,291 | - | - | - | - | - |
|  | D | MVI-45-2 | 0.63 | 58.37±3.22 | 23 | 26 | 32,124±2,365 | 34,524±2,973 | - | - | - | - | 27,276-28,405^❖^ 36,233-38,758^❖^ >33,020^❖^ |
|  |  | MVI-45-3 | 0.82 | 58.15±5.00 | 10 | 25 | 35,350±3,457 | 38,083±4,224 | - | - | - | - |  |
|  |  | MVI-45-4 | 0.97 | 65.86±3.56 | 14 | 18 | 38,047±2,752 | 41,021±3,488 | - | - | - | - |  |
|  |  | MVI-45-5 | 1.28 | 72.20±4.77 | 20 | 30 | 40,864±3,357 | 44,083±4,191 | - | - | - | - |  |
| River bank | B | MVII-D1 | 0.20 | 6.77±0.38 | 13 | 18 | 4,354±349 | 4,641±423 | 3.71±0.86 | 10 | 72 | 4,555±1,227 | - |
|  |  | MVII-D2 | 0.30 | 9.28±0.70 | 12 | 25 | 5,907±538 | 6,310±646 | - | - | - | - | - |
|  | C | MVII-D3 | 0.50 | 11.01±0.93 | 20 | 37 | 8,717±850 | 9,324±1,019 | 4.99±1.53 | 8 | 84 | 9,531±2,979 | - |
|  |  | MVII-D4 | 0.60 | 16.89±0.46 | 19 | 11 | 9,420±535 | 10,081±685 | - | - | - | - | - |
|  |  | MVII-D5 | 1.00 | 17.11±0.98 | 19 | 24 | 9,506±716 | 10,174±883 | 11.00±1.79 | 11 | 52 | 10,369±1,825 | - |
|  |  | MVII-D6 | 1.10 | 20.06±0.64 | 22 | 15 | 12,859±698 | 13,793±906 | 7.50±0.73 | 14 | 36 | 9,147±1,016 | 13,000^+^  14,190-13,990*; 14,600-14,200* |
|  | D | MVII-D7 | 1.40 | 73.65±3.64 | 24 | 24 | 39,155±2778 | 42,223±3,532 | 35.00±3.52 | 11 | 29 | 30,666±3,755 | >33,000^+^ |

+ ^14^C ages from Dillehay and Collins (1988).

* ^14^C ages from Dillehay et al. (2008).

^14^C ages from this study.

**Text H: Preliminary Micro Use-wear Study of Selected Stone Tools**

Tom D. Dillehay

Microscopic analysis was conducted by using both bright and dark field illumination. Artifacts were not cleaned prior to analysis in order to avoid loss of residues. Once it was determined that there were no residues on the edges of tools, they were cleaned for further analysis. Identification of any residues was based on observation of diagnostic anatomical features.

High magnification microscopy (100-500x) formed the core of the use-wear study, although initial macroscopic and low magnification (10-50x) inspection was undertaken to discern gross use-wear and residue deposits. All facets of each artifact were examined in incident (from above) white light using a Leica electronic microscope fitted with l0x magnification eyepieces, with 5x, l0x, and 20x objective lenses, and with 50x to 1000x long working distance (LWD) objective lenses. This allowed a maximum resolution on the artifact's surface of 1~m, or one thousandth of a millimeter. The microscope is also fitted with bright and dark field illumination, rotatable polarizing filters, and a moveable (X-Y) stage. The majority of scanning was conducted using the 50x and 100x magnifications, with higher magnifications used whenever necessary to allow for closer inspection of possible archaeological features. Transects were used to completely cover the artifact surface, not just suspected working edges, as residues may accumulate away from those areas of an artifact directly in contact with a worked material.

The same analytical protocol previously used for the MV-II lithic was employed here, including comparison of the edges of tools to experimental stones of basalt, rhyolite, andesite, quartzite, quartz, tonalite and others form the Monte Verde area [2]. In total, twenty-two of the stones were examined, with sixteen producing recognizable use-wear. These results are summarized below and shown for four specimens in S19 Figure.

Specimen 1, (S7g Fig.): Aspect 1 reveals a slight dull to semi-bright polish and diagonal to perpendicular striations on the distal end that suggest scraping a relatively hard object (arrows). Aspect 2 is located along a sharp edge near the lateral edge. It shows diagonal striations and semi-bright polish and grain loss and rounding of the edge, which suggest penetration upon impact into a target, possibly fresh hide or meat (S19a Fig.).

Specimen 2 (S7b Fig.): Aspect 1 reveals slight polish suggestive of wood or hard plant polish. Aspect 2 along the edge shows localized semi-flat areas of bright to semi-bright polish and a few minor nicks and scratches. This evidence suggests one prolonged use episode on a target or no use with the damage due to other taphonomic variables (S19b Fig.).

Specimen 3 (S7e Fig.): This flake shows spotty bright polish and minor scratches along a lateral edge. One long aspect reveals discontinuous but extensive semi-bright to bright polish and subparallel nicks and diagonal and semi-parallel striations along the edges (arrows). These suggest use as a cutting/slicing tool perhaps more than a scraping function. There also are several scratches, nicks and polish spots that are indeterminate in function (S19c Fig.).

Specimen 4 (S7c Fig.): This is a flake fragment with dull, pitted polish, narrow and wide striations along the distal area indicative of scraping and possibly gouging (Aspect 1, arrows), the latter suggested by grain loss along the edge. Other nicks and polish spots could be seen along the edges at 100-200x (Aspect 2), but they are so minor that they could be attributed to any cultural or natural cause (S19d Fig.).

**Discussion**

In this brief and preliminary study, percussion struck flakes were the most plentiful tool category in the analyzed samples. In addition to the four flakes, eighteen others from various contexts at sites MV-I and CH-1 were also examined in a preliminary fashion. Although not reported here, the results were similar to those described above, suggesting that the collection studied is fairly represented of the use-wear history on stone tools in these sites. Although the sample size is limited in this preliminary study, eleven of the percussion struck flakes analyzed possessed use-wear. Many flakes seemed to have been used expediently; there is very little retouch and resharpening evident on the stones. A few flakes suggest multiple tasks. The remaining artifacts did not or were indeterminate. Several worked edges had clear evidence of use, as shown in the S19 Fig., but the material worked could not be determined with any degree of confidence. Several tools also may have been recycled or the tasks may be related to a single complex activity.

A few tools had multiple edges used for several tasks, such as possible meat cutting, plant working and hide manufacture. Unfortunately, the relationship between these different kinds of tasks cannot be determined. Furthermore, based on this preliminary study, it is speculative to infer the importance of any specific use activity over another, because of the small sample size and the proportion of tools whose function could not always be confidently determined to the type of material worked.

**S19 Fig**. Micro Use-wear (arrows) on the edges of four worked stone tools.


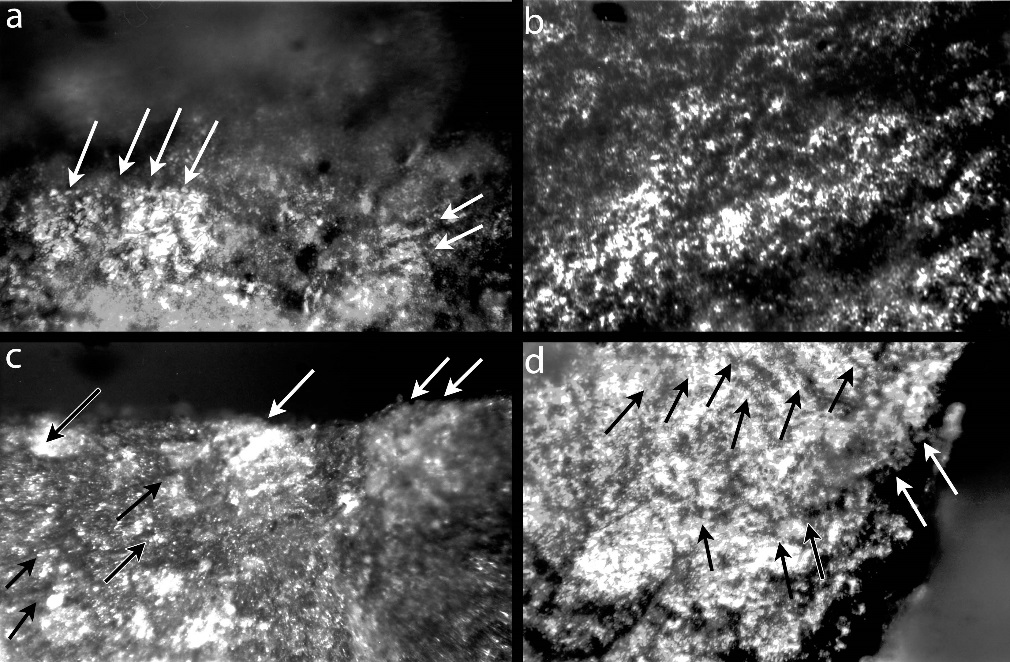


**References**

1. Dillehay TD. Monte Verde: A Late Pleistocene Settlement in Chile. Volume I: The Paleo‑environment and Site Context. Washington, D.C.: Smithsonian Institution Press; 1989.

1. Dillehay TD. Monte Verde: A Late Pleistocene Settlement in Chile. Volume II: The Archaeological Context. Washington, D.C.: Smithsonian Institution Press; 1997.

1. Dillehay TD, Collins MB. Early cultural evidence from Monte Verde in Chile. Nature 1998; 332: 150-152.

1. Collins MB. The Lithics from Monte Verde, A Descriptive-Morphological Analysis. In: Dillehay TD, editor. Monte Verde: A Late Pleistocene Settlement in Chile. Volume II: The Archaeological Context. Washington, D.C.: Smithsonian Institution Press; 1997. pp 383-506.

1. Pino M. Geology. In: Dillehay TD, editor. Monte Verde, Vol. I: Palaeoenvironment and site context. Washington, D.C.: Smithsonian Institution Press; 1989. pp. 89-132.
2. Denton GH, Lowell TV, Heusser CJ, Schlüchter C, Andersen BG, Heusser LE, et al. Geomorphology, Stratigraphy, and Radiocarbon Chronology of Llanquihue Drift in the Area of the Southern Lake District, Seno Reloncavi, and Isla Grande de Chiloé, Chile. Geografiska Annaler. 1999; 81(2): 167-229.
3. Sellés D, Moreno H. Geología del volcán Calbuco, Región de Los Lagos (map). Servicio Nacional de Geología y Minería, Carta Geológica de Chile, Serie Geología Básica; 2011. 130: 38-43.

1. Watt S, Pyle DM, Naranjo JA, Rosqvist G, Mella M, Mather TA, et al. Holocene tephrochronology of the Hualaihue region (Andean southern volcanic zone, ∼42° S), southern Chile. Quat. Internat. 2011; 246: 324-343.

1. López-Escobar L, Parada MA, Hickey-Vargas R, Frey FA, Kempton PD, Moreno H. Calbuco Volcano and minor eruptive centers distributed along the Liquiñe-Ofqui Fault Zone, Chile (41°-42°S): contrasting origin of andesitic and basaltic magma in the Southern Volcanic Zone of the Andes. Contrib. Mineralogy Petr. 1995; 119: 345-361.

1. Hickey-Vargas R, Abdollahi MJ, Parada MA, López-Escobar L, Frey FA. Crustal xenoliths from Calbuco Volcano, Andean Southern Volcanic Zone: implications for crustal composition and magma-crust interaction. Contri. Mineralogy Petr. 1995; 119: 331-344.

11. Stoops G. Guidelines for Analysis and Description of Soil and Regolith Thin Sections. Madison, WI: Soil Science Society of America; 2003

1. Mücher H, van Steijn H, Kwaad, F (2010). Interpretation of micromorphological features of soils and regoliths (eds Stoops, G., Marcelino, V., Mees, F.) 37-48 (Elsevier).

1. Stoops G. Micromorphology of soils derived from volcanic ash in Europe  : a review and synthesis. Eur. J. Soil Sci. 2007; 58: 356–377.

1. Arnalds, O. Andosols. In: Chesworth W, editor. Encyclopedia of Soil Science. Dordrecht, Netherlands: Springer; 2008. pp. 39-46.

1. Gérard M, Caquineau S, Pinheiro J, Stoops G. Weathering and allophane neoformation in soils developed on volcanic ash in the Azores. Eur. J. Soil Sci. 2007; 58: 496-515.

1. Sedov S, Stoops G, Shoba S. In: Stoops G, Marcelino V, Mees F, editors. Interpretation of

micromorphological features of soils and regoliths. Amsterdam: Elsevier; 2010. pp 275-304.

1. Evans ME, Heller F. Environmental Magnetism: Principles and Applications of Enviromagnetics. Boston: Academic; 2003.

1. Liu Q, Roberts AP, Larrasoaña JC, Banerjee SK, Guyodo Y, Tauxe L. Environmental Magnetism: Principles and Applications. Rev. Geophys. 2012; 50, RG4002. doi:10.1029/2012RG000393.

1. Heusser CJ. Pollen and Spores of Chile, Modern Types of the Pteridophyta, Gymnospermae, and Angiospermae. Tuscon: University of Arizona Press; 1971.

1. Markgraf V, D'antoni HL. Pollen Flora of Argentina, Modern Spore and Pollen Types of

Pteridophyta, Gymnospermae, and Angiospermae Tuscon: University of Arizona Press; 1978.

1. Brown DA. Prospects and Limits of Phytolith Key for Grasses in the Central Unitsed States. J Archeol Sci. 1994; 11: 345-368.

22. Gould FN, Shaw RB. Grass Systematics. College Station: Texas A&M University Press; 1983.

23. Twiss PC. Grass-opal phytoliths as climatic indicators of the Great Plains Pleistocene. In: Johnson WC, editor. Quaternary Environments of Kansas. Lawrence, KS: University of Kansas Press; 1987. pp. 179-188.

24. Piperno DR. Phytoliths: A Comprehensive Guide for Archaeologists and Paleoecologists Lanham, MD: Alta Mira Press; 2006.

25. Jones RL, Beavers AH. Sponge Spicules in Illinois Soils. Soil Science Proceed. 1963; 27: 438-440.

26. Heusser CJ. Pollen Analysis. In: Dillehay TD, editor. Monte Verde, Volume I: Palaeoenvironment and site context. Washington, D.C.: Smithsonian Institution Press; 1989. pp. 183-99.

27. Facciola S. Cornucopia: A Source Book of Edible Plants. Vista, CA: Kampong Publications; 1990.

28. Murray AS, Wintle AG. The single aliquot regeneration dose protocol: potential for improvements in reliability. Radiat. Meas. 2003; 37: 377-381.

29. Buylaert J-P, Thiel C, Murray AS, Vandenberghe DAG, Yi S, Lu H. IRSL and post-IR IRSL residual doses recorded in modern dust samples from the Chinese Loess Plateau. Geochron. 2011; 38: 432-440.

30. Buylaert J-P, Jain M, Murray AS, Thomsen KJ, Thiel C, Sohbati R. A robust feldspar luminescence dating method for Middle and Late Pleistocene sediments. Boreas. 2012; 41: 435-451.

31. Spooner NA. The anomalous fading of infrared-stimulated luminescence from feldspars. Radiat. Meas. 1994; 23: 625-32.

32. Krbetschek MR, Götze J, Dietrich A, Trautmann T. Spectral information from minerals relevant for luminescence dating. Radiat. Meas. 1997; 27: 695-748.

33. Rhodes EJ. Optically Stimulated Luminescence Dating of Sediments over the Past 200,000 Years. Annu. Rev. Earth. Pl. Sc. 2011; 39: 461-88.

34. Thomsen KJ, Murray AS, Jain M, Botter-Jensen L. Laboratory fading rates of various luminescence signals from feldspar-rich extracts. Radiat. Meas. 2008; 43: 1474-86.

35. Buylaert JP, Murray AS, Thomsen KJ, Jain M. Testing the potential of an elevated temperature IRSL signal from K-feldspar. Radiat. Meas. 2009; 44: 560-65.

36. Galbraith RF, Roberts RG, Laslett GM, Yoshida H, Olley JM. Optical dating of single and multiple grains of quartz from Jinmium rock shelter, northern Australia: Part I. Experimental design and statistical models. Archaeometry. 1999; 41: 339-64.

37. Huntley DJ, Lamothe M. Ubiquity of anomalous fading in K-feldspars and the measurement and correction for it in optical dating. Canadian J. Earth Sci. 2001; 38:1093-1106.

38. Auclair M, Lamothe M, Huot S. Measurements of anomalous fading for feldspar IRSL using SAR. Radiat. Meas. 2003; 37: 487-92.

39. Adamiec G, Aitken MJ. Dose-rate conversion factors: new data. Ancient TL. 1998; 16: 37–50.

40. Huntley DJ, Barril MR. The K content of the K-feldspars being measured in optical dating or in thermoluminescence dating. Ancient TL. 1997; 15: 11-13.

41. Prescott JR, Stephan LG. The contribution of cosmic radiation to the environmental dose for thermoluminescence dating: latitude, altitude and depth dependencies. J. Council Europe PACT. 1992; 6: 17-25.

42. Preusser F, Ramseyer K, Schlüchter C. Characterization of low OSL intensity quartz from the New Zealand Alps. Radiat. Meas. 2006; 41: 871-77.

43. Arnold LJ, Roberts RG. Stochastic modeling of multi-grain equivalent dose (De) distributions: implications for OSL dating of sediment mixtures. Quat. Geochron. 2009; 4: 204-30.

44. Dillehay TD, Ramírez C, Pino M, Collins MB, Rossen J, Pino-Navarro JD, et al. Monte Verde: Seaweed, Food, Medicine, and the Peopling of the Americas. Science. 2008; 320: 784-6.

45. Dillehay TD, Pino M. Chronology and Stratigraphy. In: Dillehay TD, editor. Monte Verde. Vol. I: Palaeoenvironment and site context. Washington, D.C.: Smithsonian Institution Press; 1989. pp. 133-46.
